# Vitamins C, E, and β-Carotene and Risk of Type 2 Diabetes: A Systematic Review and Meta-Analysis

**DOI:** 10.1016/j.advnut.2024.100211

**Published:** 2024-03-15

**Authors:** Anna-Maria Lampousi, Therese Lundberg, Josefin E Löfvenborg, Sofia Carlsson

**Affiliations:** 1 Institute of Environmental Medicine, Karolinska Institutet, Stockholm, Sweden; 2 Department of Neurobiology, Care Sciences and Society, Karolinska Institutet, Huddinge, Sweden; 3 Department of Risk and Benefit Assessment, Swedish Food Agency, Uppsala, Sweden

**Keywords:** type 2 diabetes, insulin resistance, vitamin C, vitamin E, β-carotene, antioxidants, systematic review, meta-analysis

## Abstract

A systematic review and meta-analysis was conducted to assess the relationship between the common dietary antioxidants vitamin C, vitamin E, and β-carotene and type 2 diabetes (T2D) and related traits. MEDLINE, Embase, and the Cochrane Library were searched for relevant publications up until May 2023. Studies were eligible if they had a cohort, case–control, or randomized controlled trial (RCT) design and examined dietary intake, supplementation, or circulating levels of these antioxidants as exposure, and insulin resistance, β-cell function, or T2D incidence as outcomes. Summary relative risks (RR) or mean differences (MD) with 95% confidence intervals (CI) were estimated using random-effects models. The certainty of the evidence was assessed with the Grading of Recommendations, Assessment, Development and Evaluations framework. Among 6190 screened records, 25 prospective observational studies and 15 RCTs were eligible. Inverse associations were found between dietary and circulating antioxidants and T2D (observational studies). The lowest risk was seen at intakes of 70 mg/d of vitamin C (RR: 0.76; CI: 0.61, 0.95), 12 mg/d of vitamin E (RR: 0.72; CI: 0.61, 0.86), and 4 mg/d of β-carotene (RR: 0.78; CI: 0.65, 0.94). Supplementation with vitamin E (RR: 1.01; CI: 0.93, 1.10) or β-carotene (RR: 0.98; CI: 0.90, 1.07) did not have a protective effect on T2D (RCTs), and data on vitamin C supplementation was limited. Regarding insulin resistance, higher dietary vitamin C (RR: 0.85; CI: 0.74, 0.98) and vitamin E supplementation (MD: –0.35; CI: –0.65, –0.06) were associated with a reduced risk. The certainty of evidence was high for the associations between T2D and dietary vitamin E and β-carotene, and low to moderate for other associations. In conclusion, moderate intakes of vitamins C, E, and β-carotene may lower risk of T2D by reducing insulin resistance. Lack of protection with supplementation in RCTs suggests that adequate rather than high intakes may play a role in T2D prevention. This systematic review and meta-analysis was registered in PROSPERO with registration number CRD42022343482.

## Graphical abstract

**Figure:**
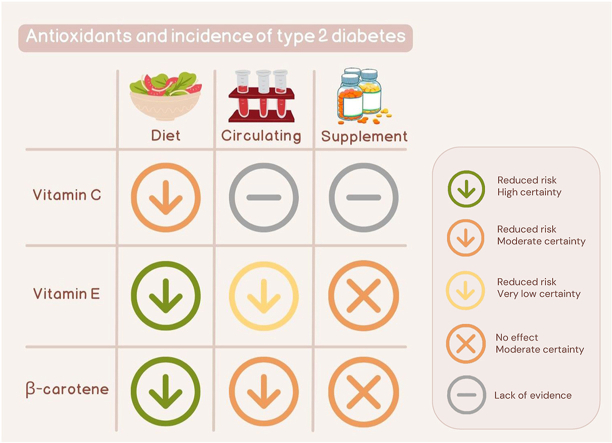

**Statements of Significance**

This systematic review and meta-analysis is the first to examine the dose–response relationships of vitamin C, vitamin E, and β-carotene with type 2 diabetes risk, incorporating both observational and interventional studies and assessing smoking as an effect modifier. It also evaluates the outcomes of insulin resistance and β-cell function, providing a new insight into the underlying mechanisms.

## Introduction

Type 2 diabetes (T2D) is a serious health issue that affects over 10% of the world’s population and involves a combination of insulin resistance and β-cell dysfunction [1]. Lifestyle interventions that induce weight loss, such as diet and exercise, are effective in preventing T2D in high-risk individuals [2,3]. However, dietary interventions may exert additional benefits for T2D prevention beyond reducing body weight. Evidence from prospective studies suggests that adherence to healthy dietary patterns, such as the Mediterranean or Dietary Approach to Stop Hypertension diets, may reduce risk of T2D [4,5]. A common characteristic of these diets is their emphasis on plant-based foods, such as fruits, vegetables, and plant-based oils, which are excellent sources of antioxidants, particularly vitamin C, vitamin E, and β-carotene.

Antioxidants are compounds that protect the cells from oxidative stress, which occurs when the body’s production of free radicals exceeds its ability to neutralize them and is implicated in the pathogenesis of T2D [6]. Specifically, oxidative stress has been shown to increase insulin resistance through mechanisms such as inhibition of insulin signaling and promotion of inflammatory processes [7]. In accordance with these findings, a high dietary antioxidant capacity has been inversely associated with insulin resistance and T2D [8,9]. Nevertheless, each antioxidant has a unique biological function and the specific effects, mechanisms, and optimal doses of different antioxidants remain to be established [10]. Previous meta-analyses have suggested a protective effect of vitamin E and β-carotene, but not vitamin C, on T2D [11,12]. However, these analyses did not include data from randomized controlled trials (RCTs) or assess potential mechanisms such as effects on insulin resistance or β-cell function, and there are limited data on dose–response relationships. Furthermore, inverse associations with these antioxidants have mainly been observed among nonsmokers [13,14], but the potential influence of smoking as an effect modifier has not been considered in previous meta-analyses.

The aim of this systematic review and dose–response meta-analysis is to elucidate the role of vitamin C, vitamin E, and β-carotene in the prevention of T2D. This involves synthesizing and evaluating the totality of evidence from observational studies and RCTs that examined the association of these antioxidants with T2D incidence, insulin resistance, or β-cell function. This study also examines if smoking has any impact on these associations. By providing a comprehensive understanding of these relationships, this study aims to inform dietary recommendations for the prevention of T2D.

## Methods

This study was reported following the PRISMA guidelines [15]. A protocol for this study was registered in PROSPERO with registration number CRD42022343482.

### Search strategy and selection criteria

The literature search was performed in MEDLINE (Ovid), Embase, and the Cochrane Library, from inception until July 2022 and updated in May 2023 by librarians at Karolinska Institutet. The complete search strategy is shown in Supplemental Tables 1–3. Eligible studies were those that used a cohort, case–cohort, case–control, nested case–control, or RCT design to investigate the association between dietary intakes, circulating levels, or supplementation of vitamin C, vitamin E, and β-carotene and T2D incidence or insulin resistance/sensitivity and β-cell function in individuals who were diabetes-free at baseline. Conference/congress articles, editorials, interviews, letters, animal studies, and articles written in languages other than English were excluded. Studies in the reference lists of eligible articles and relevant systematic reviews and meta-analyses were also screened for eligibility. Two authors (AML and TL) independently screened the titles and abstracts of the identified articles and examined the full-text versions of the potentially eligible articles. Disagreements were resolved after consultation with a third author (SC).

### Data extraction

The following study information was extracted independently by 2 authors (AML and TL): first author’s name, journal, publication year, funding information, study design, data collection year, country, cohort name, number of participants, number of exposed/unexposed cases, number of exposed/unexposed noncases, sex, age at baseline, dietary intake or circulating levels of vitamin C, vitamin E, and β-carotene, micronutrient supplementation (type, frequency and yes/no), reference group, outcome, type of prevalent health conditions, smoking prevalence, exposure and outcome assessment methods, response rates, percentage of participants lost to follow-up, follow-up time, person-time, most adjusted measure of relative effect with 95% confidence intervals (CIs) for dichotomous (risk ratios/odds ratios) or time-to-event (hazard ratios) outcome data, and pre-post intervention mean change with SD for continuous outcome data, and factors adjusted for in the models. If estimates were only provided for subgroups, for example, men and women, they were pooled using fixed-effect models before being included in meta-analyses. When several articles used overlapping data, only the one with the largest sample size was included, and when multiple studies used the same data the most recent one was included.

### Risk of bias assessment

Two authors (AML and TL) independently used the Risk of Bias in Non-randomized Studies of Interventions (ROBINS-I) tool [16] and the revised tool for Risk of Bias in Randomized Trials (RoB 2) [17] to evaluate the risk of bias in individual studies. In ROBINS-I, the risk of 7 sources of bias in observational studies, including confounding, selection of participants into the study, classification of interventions, deviations from intended interventions, missing data, outcome measurement, and selection of reported results, is rated as low, moderate, serious, or critical. Studies adjusting for age, sex, BMI (kg/m^2^), and lifestyle factors, including smoking and physical activity, were rated as having a moderate risk of bias because of confounding. Not accounting for ≥1 of these factors resulted in a serious risk rating. Similarly, in RoB 2 the risk of 5 sources of bias in RCTs, including the randomization process, deviations from intended interventions, missing data, outcome measurement, and selection of reported result, is rated as low, some concerns, or high. The overall risk of bias in each study corresponds to the rating of the domain with the highest risk of bias.

### Statistical analysis

Summary relative risks (RRs) and 95% CIs for T2D were estimated in relation to high compared with low dietary intakes or circulating levels of vitamin C, vitamin E, and β-carotene (observational studies) and for supplementation with these antioxidants compared with placebo (RCTs) using a random-effects model. Accordingly, the weighted average of the natural logarithm of the RRs was obtained accounting for both within- and between-study variances, and the restricted maximum likelihood method was used for estimating the latter. Similarly, linear dose–response meta-analyses were performed to assess dietary intakes or circulating levels of vitamin C, vitamin E, and β-carotene as continuous variables. For studies that did not report such estimates, the method described by Greenland and Longnecker [18] was used to estimate study-specific slopes and 95% CIs from the natural logarithm of the RRs and 95% CIs across exposure categories. This analysis requires data on the number of cases, person-time, mean/median exposure level, and corresponding RRs and 95% CIs across ≥3 exposure categories. When the distribution of cases or person-time was not reported, they were estimated by dividing their total number by the number of exposure quintiles. If total person-time was not reported, it was estimated by multiplying the average follow-up time by the total number of study participants. When the exposure category was reported as a range, the midpoint was considered as the median. If the lowest exposure category was open-ended, the lowest value was assumed to be 0, and if the highest exposure level was open-ended, a width similar to the former category was assumed. All units were converted to mg/d for dietary intakes and μmol/L for circulating levels. For studies reporting circulating alpha-tocopherol in mg/dL or β-carotene in μg/dL, concentrations were multiplied by 23.22 and 0.01863, respectively [19]. Linear trends were estimated per 10 mg/d for dietary vitamin C, per 1 mg/d for dietary vitamin E or β-carotene, and per 1 SD increment for circulating levels. Nonlinear dose–response meta-analyses were additionally performed by fitting a random-effects restricted cubic spline model with 3 knots at the 10th, 50th, and 90th percentiles of exposure frequency [20].

Random-effects models were also used for obtaining summary RRs and 95% CIs of insulin resistance in relation to dietary vitamin C and vitamin E intakes (observational studies) and for estimating the mean difference (MD) with 95% CIs of the change in continuous estimates of insulin resistance from baseline between individuals using vitamin E supplements or not (RCTs). When SDs of the changes were not provided or there was not enough information available to calculate them, those were imputed according to methods described in the Cochrane Handbook [21].

Heterogeneity among studies was tested with Cochran’s *Q* test and quantified with the *I*^2^ statistic, with an *I*^2^ > 50% being indicative of substantial heterogeneity. Subgroup and meta-regression analyses were performed to detect possible sources of heterogeneity. Subgroups were formulated based on age, sex, geographic region, health condition, and risk of bias, whereas smoking prevalence was included in meta-regression analyses. Moreover, sensitivity analyses were performed by excluding studies that did not adjust for dietary co-exposures.

All statistical analyses were performed with Stata Statistical Software Release 16 (StataCorp). Results were statistically significant when the 95% CI did not include the null value, that is, 1 for summary RRs and 0 for MD.

### Certainty of evidence assessment

The Grading of Recommendations, Assessment, Development, and Evaluations (GRADE) framework was used to assess the certainty of evidence for each meta-analysis [22]. According to this framework, a high level of certainty is initially assigned to meta-analyses of RCTs and meta-analyses of observational studies that were assessed for bias using the ROBINS-I tool [23]. Subsequently, certainty of evidence may be downgraded if serious risk of bias, imprecision, inconsistency, indirectness, or publication bias are present, or upgraded if a large effect (RR <0.5 or RR >2) or dose–response gradient is present and if all plausible bias would attenuate an association [24]. Typically, domains marked as “serious” warrant a cautious approach to upgrading certainty levels. However, if ROBINS-I results in a moderate risk rating because of confounding, the risk of the bias domain in GRADE does not need to be downgraded if a strong effect or a dose–response gradient is present [23]. This is because large effect sizes and dose–response relationships can mitigate the influence of residual confounding. Finally, the certainty of evidence level is classified as “very low,” “low,” “moderate,” or “high.”

## Results

**FIGURE 1:**
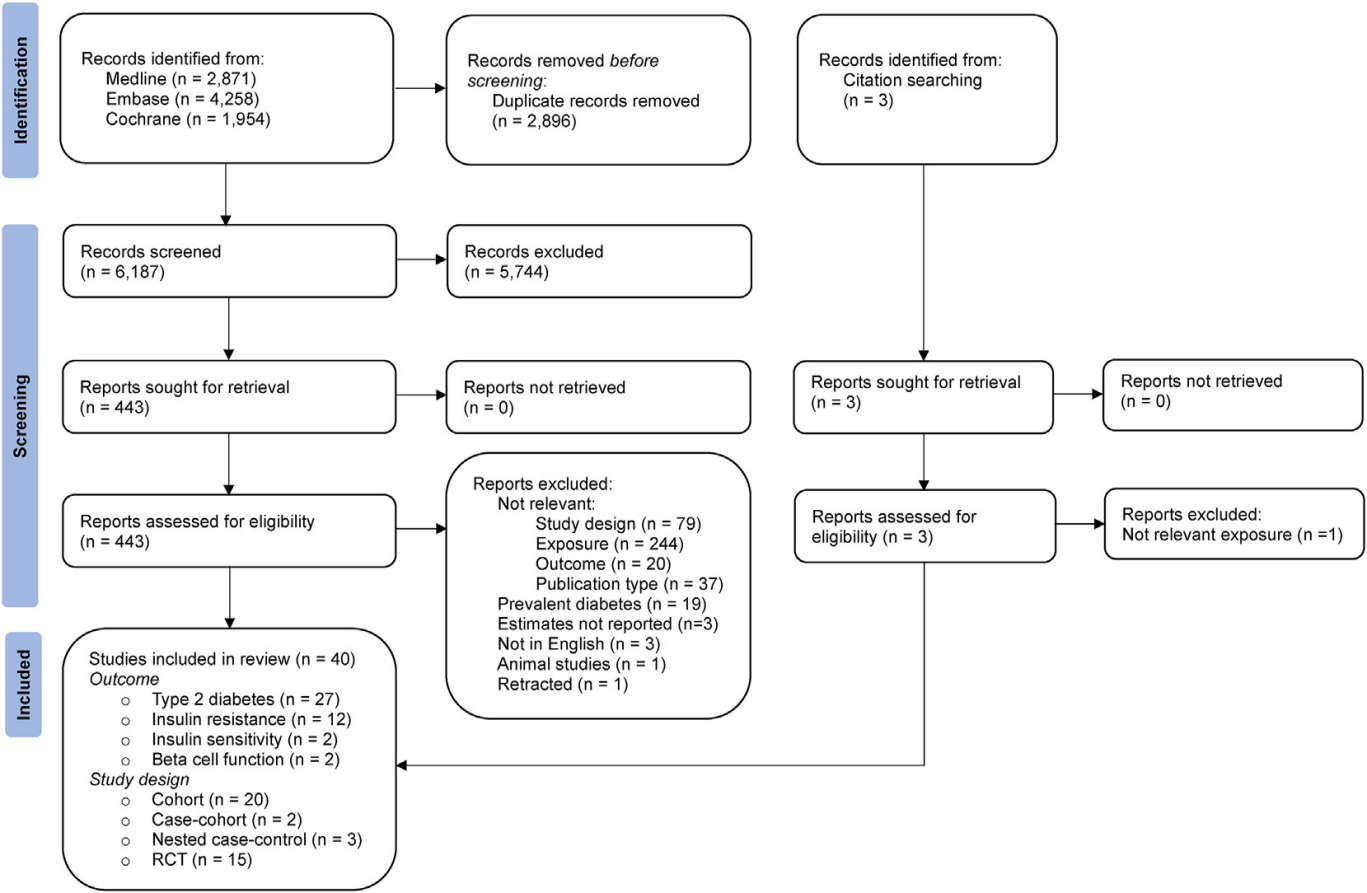
Flow diagram of study selection.

**FIGURE 2:**
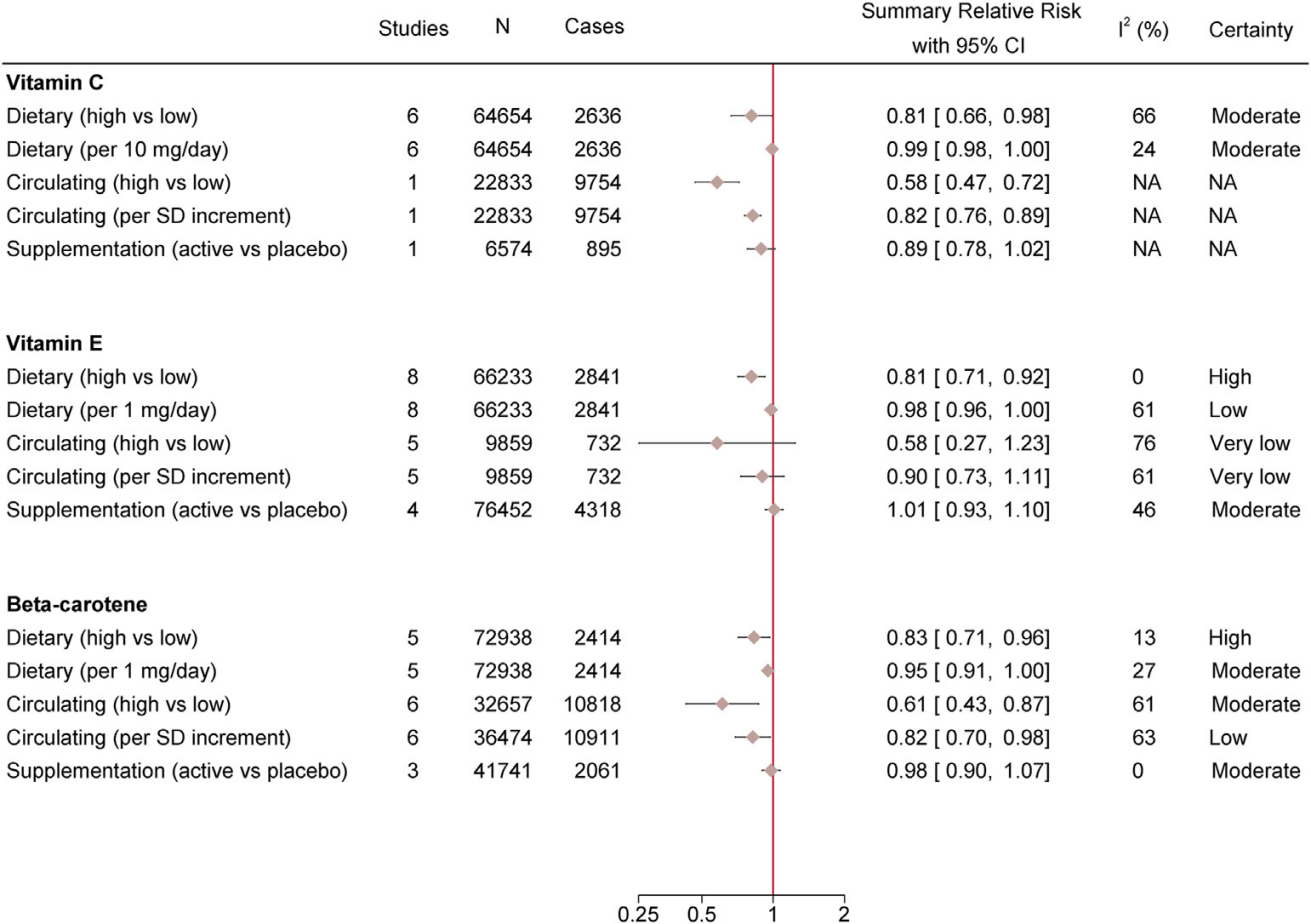
Summary relative risks with 95% CIs for the associations between dietary intakes (prospective observational studies), circulating levels (prospective observational studies), or supplementation (randomized controlled trials) of vitamin C, vitamin E, or β-carotene and incidence of type 2 diabetes. CI, confidence interval; NA, not applicable.

**FIGURE 3:**
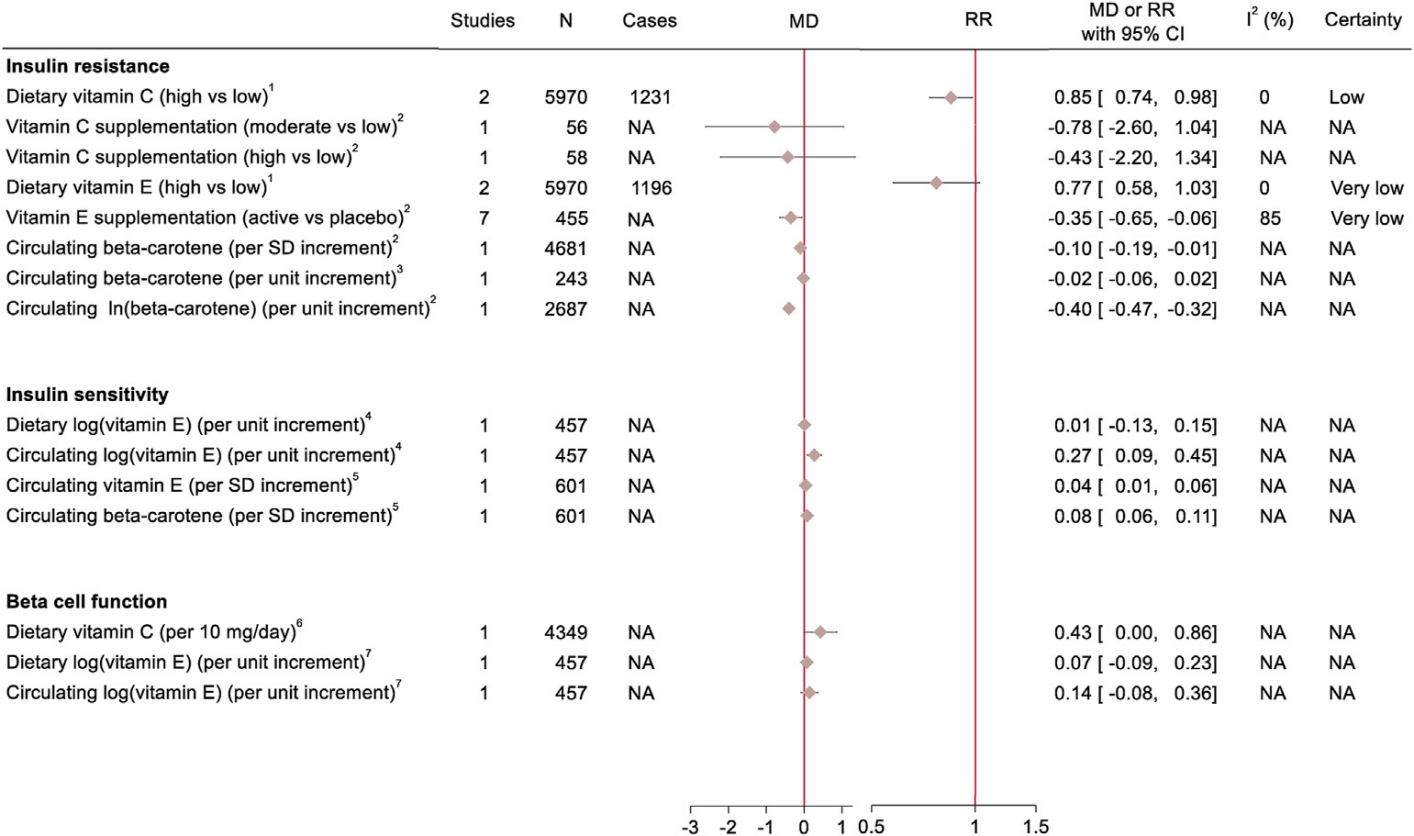
Mean differences or summary relative risks with 95% CIs for the associations between dietary intakes (prospective observational studies), circulating levels (prospective observational studies), or supplementation (randomized controlled trials) of vitamin C, vitamin E, or β-carotene and insulin resistance, insulin sensitivity, and β-cell function. ^1^≥75th percentile of the HOMA-IR, ^2^HOMA-IR, ^3^log(HOMA-IR), ^4^log(S_I_+1), ^5^HOMA-S, ^6^HOMA-B, and ^7^log(AIR+397). AIR, acute insulin response to glucose; CI, confidence interval; HOMA-B, homeostatic model assessment of β-cell function; HOMA-S, homeostatic model assessment of insulin sensitivity; MD, mean difference; NA, not applicable; RR, relative risk; S_I_, insulin sensitivity index.

After screening 6190 articles, 40 eligible studies with either cohort (*n* = 20), case–cohort (*n* = 2), nested case–control (*n* = 3), or RCT (*n* = 15) design were identified (Figure 1). Studies that were deemed to be ineligible after full-text screening, along with the reasons for their exclusion, are shown in Supplemental Table 4. The characteristics of all eligible studies are displayed in Supplemental Tables 5 and 6. Observational studies evaluated either dietary intakes or circulating levels of vitamin C, vitamin E, and β-carotene, whereas RCTs assessed supplementation with these antioxidants. Of the observational studies, 21 had a moderate and 4 had a serious risk of bias (Supplemental Figure 1), and of the RCTs, 13 had a low risk of bias and 2 had some concerns (Supplemental Figure 2). Most studies investigated T2D (*n* = 27) as the outcome followed by HOMA-IR (*n* = 12) [25], HOMA-S or insulin sensitivity index (*n* = 2) [26], and HOMA-B or insulin secretion estimated by acute insulin response to glucose (AIR) (*n* = 2) [27]. There was an equal number of studies conducted in Europe, Asia, and North America (*n* = 13) and a single study from New Zealand. Nine studies were not included in the meta-analysis because of overlapping data with other eligible studies (Supplemental References 6, 11, 12, and 26) or unique exposure/outcome definitions (Supplemental References 17, 28–30, and 38). Figure 2 shows the summary RRs and 95% CIs of T2D in relation to the antioxidants. For vitamin C, only dietary intakes could be meta-analyzed; however, to provide a complete picture, RRs from single studies of circulating levels and supplementation are shown in Figure 2. For insulin resistance, meta-analyses could be performed only in relation to dietary intakes of vitamin C and vitamin E and supplementation with vitamin E, whereas the outcomes of insulin sensitivity and β-cell function were not subjected to meta-analysis because of the lack of studies (Figure 3). The forest plot for each meta-analysis is illustrated in Supplemental Figures 3–17.

### Vitamin C

#### Type 2 diabetes

**FIGURE 4:**
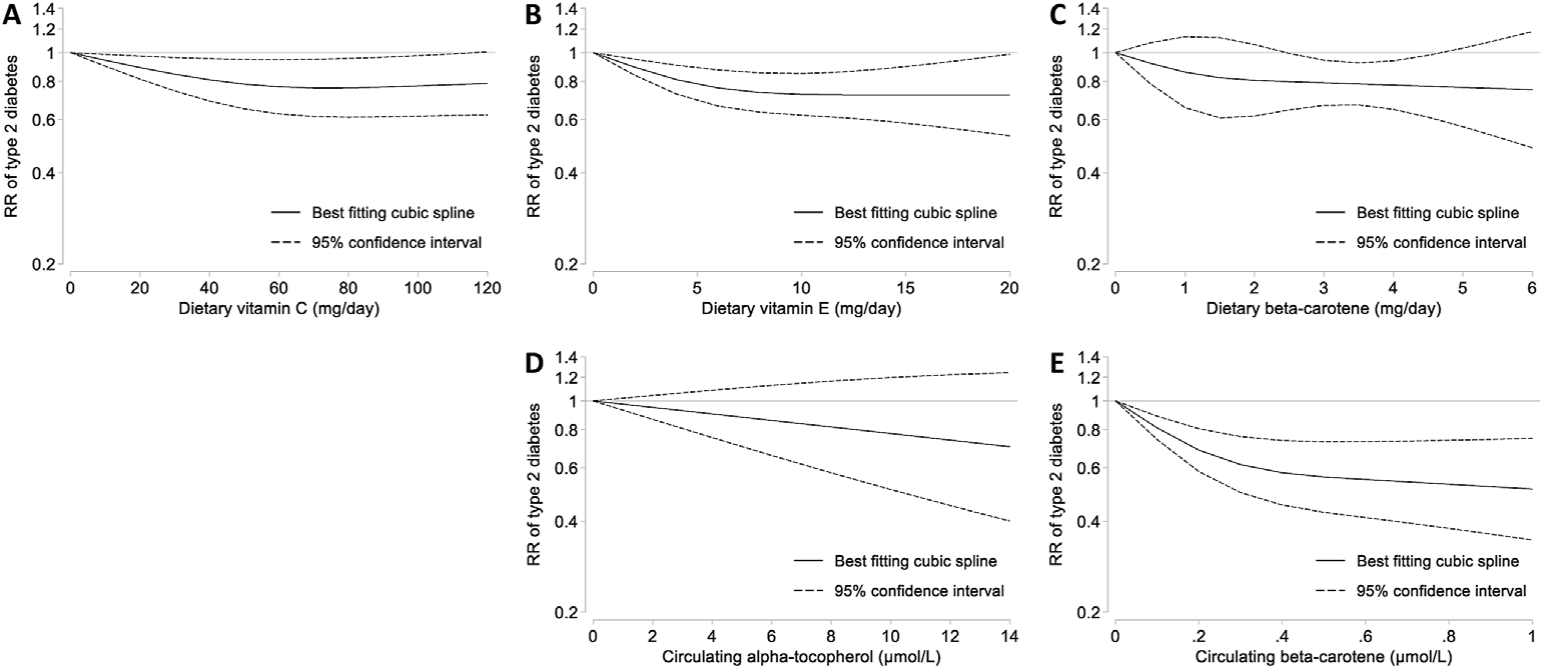
Nonlinear dose–response meta-analyses for the association between dietary intakes of (A) vitamin C (*P*_nonlinearity_ = 0.034), (B) vitamin E (*P*_nonlinearity_ < 0.001) and (C) β-carotene (*P*_nonlinearity_ = 0.014) or circulating levels of (D) alpha-tocopherol (*P*_nonlinearity_ = 0.402) and (E) β-carotene (*P*_nonlinearity_ < 0.001), and incidence of type 2 diabetes.

**FIGURE 5:**
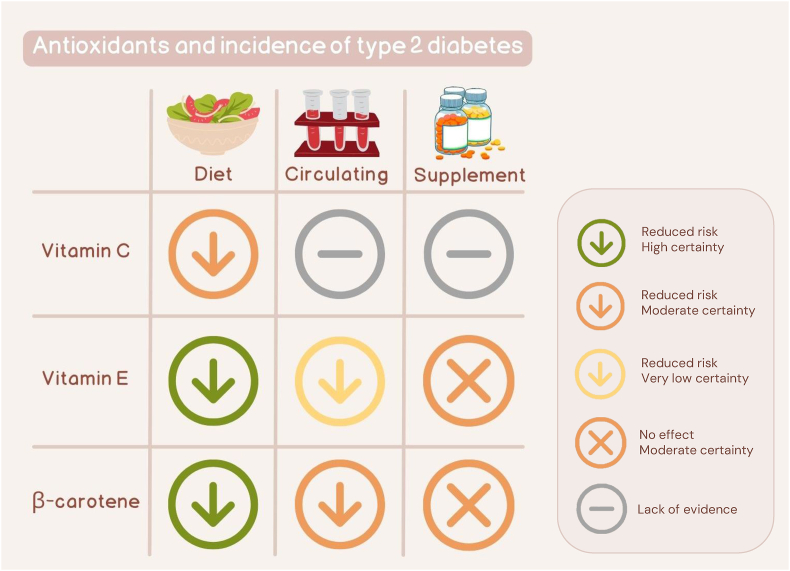
Certainty of evidence of the associations between dietary intakes (prospective observational studies), circulating levels (prospective observational studies), or supplementation (randomized controlled trials) of vitamin C, vitamin E, or β-carotene and incidence of type 2 diabetes, based on the GRADE framework. GRADE, Grading Of Recommendations, Assessment, Development, and Evaluations.

A reduced risk of T2D was found for high compared with low dietary vitamin C, with substantial heterogeneity between studies (RR: 0.81; 95% CI: 0.66, 0.98, *I*^2^ = 66%) (Figure 2 and Supplemental Figure 3). Meta-regression analysis revealed that 62% of this heterogeneity was explained by smoking prevalence; the inverse association tended to be stronger in studies with lower smoking prevalence (Supplemental Figure 18). Subgroup analyses were only possible by sex and geographic region, but the limited number of studies within subgroups precluded the assessment of these factors as effect modifiers (Supplemental Figures 19 and 20). In addition, the inverse association persisted after restricting the analyses to studies that had adjusted for dietary co-exposures (Supplemental Figure 21). Furthermore, there was evidence of a nonlinear dose–response relationship between dietary vitamin C and T2D; an intake of 70 mg/d was associated with a 24% lower risk of T2D (RR: 0.76; 95% CI: 0.61, 0.95), whereas a higher intake was not associated with a further risk reduction (Figure 4). Considering all these factors, the inverse association between dietary vitamin C and T2D was rated with moderate certainty (Figure 5 and Supplemental Table 7). Likewise, the European Prospective Investigation into Cancer and Nutrition-InterAct study showed an inverse association between circulating vitamin C and T2D both for high compared with low (RR: 0.58; 95% CI: 0.47, 0.72) and per 1 SD increment (RR: 0.82; 95% CI: 0.76, 0.89) in vitamin C levels [28]. Only 1 RCT investigated the effect of vitamin C supplementation compared with placebo on T2D incidence, which failed to identify a statistically significant effect (RR: 0.89; 95% CI: 0.78, 1.02) [29].

#### Insulin resistance/sensitivity and β-cell function

Dietary vitamin C was inversely associated with insulin resistance in a meta-analysis of 2 Chinese cohorts (RR: 0.85; 95% CI: 0.74, 0.98, *I*^2^ = 0%) (Figure 3 and Supplemental Figure 5). This association was rated with low certainty (Supplemental Table 7). Moreover, one of the cohorts found that each 10 mg/d increment of dietary vitamin C was associated with a 0.43 unit increase in β-cell function (95% CI: 0.00, 0.86) (Figure 3 and Supplemental Reference 27). A single RCT investigated the effect of vitamin C supplementation on insulin resistance, and the results were compatible with a reduced risk, although not statistically significant (Figure 3 and Supplemental Reference 30). No study investigated the association between circulating vitamin C and insulin resistance or β-cell function.

### Vitamin E

#### Type 2 diabetes

High compared with low dietary vitamin E intake was associated with a reduced risk of T2D, without any indication of heterogeneity across studies (RR: 0.81; 95% CI: 0.71, 0.92, *I*^2^ = 0%) (Figure 2 and Supplemental Figure 6), and that was consistent among studies that had adjusted for dietary co-exposures (Supplemental Figure 22). Certainty for this association was rated as high (Figure 5 and Supplemental Table 7). There was evidence of a nonlinear dose–response relationship; the risk of T2D decreased by 28% with an intake of 12 mg/d (RR: 0.72; 95% CI: 0.61, 0.86) and further increases in intake were not related to additional risk reduction (Figure 4). Results for circulating levels of vitamin E (alpha-tocopherol) were similar (FIGURE 2, FIGURE 4) but not statistically significant, and the heterogeneity was substantial (RR: 0.58; 95% CI: 0.27, 1.23, *I*^2^ = 76%). Consequently, this association was rated with very low certainty (Figure 5 and Supplemental Table 7). Smoking prevalence explained 84% of the between-study variance; vitamin E showed stronger inverse associations with T2D risk in studies with less smokers (Supplemental Figure 23). Heterogeneity persisted in analyses stratified by sex, geographic region, and risk of bias (Supplemental Figures 24–26). Finally, there was no support that vitamin E supplementation reduces the risk of T2D in our meta-analysis of RCTs (RR: 1.01; 95% CI: 0.93, 1.10, *I*^2^ = 46%) (Supplemental Figure 11). The certainty of this null finding was rated as moderate (Figure 5 and Supplemental Table 7).

#### Insulin resistance/sensitivity and β-cell function

No statistically significant association was observed between high compared with low dietary vitamin E and insulin resistance (RR: 0.77; 95% CI: 0.58, 1.03, *I*^2^ = 0%) in a meta-analysis of 2 Chinese cohorts (Figure 3 and Supplemental Figure 8), although certainty was very low (Supplementary Table 7). There was, however, a positive association between circulating vitamin E and insulin sensitivity in 2 prospective cohorts from the United States and Sweden (Figure 3 and Supplemental References 1 and 38), respectively, but these could not be meta-analyzed because of differences in the outcome assessment. Beneficial effects of vitamin E on insulin sensitivity were also supported by our meta-analysis of RCTs, which compared the effect of vitamin E supplementation compared with placebo or lifestyle intervention on HOMA-IR levels (MD: –0.35; 95% CI: –0.65, –0.06, *I*^2^ = 85%) (Figure 3 and Supplementary Figure 12). However, heterogeneity was high and could not be explained by stratified analyses (Figure 3 and Supplementary Figures 27–31), and meta-regression analysis including smoking prevalence was not possible. This resulted in rating the effects of vitamin E supplementation on HOMA-IR with very low certainty (Supplementary Table 7). Neither dietary nor circulating vitamin E was associated with β-cell function in a single cohort study (Figure 3 and Supplemental Reference 38).

### β-carotene

#### Type 2 diabetes

A reduced risk of T2D was observed for high compared with low dietary β-carotene, with minor heterogeneity across studies (RR: 0.83; 95% CI: 0.71, 0.96, *I*^2^ = 13%) (Figure 2 and Supplemental Figure 13) and high certainty of evidence (Figure 5 and Supplemental Table 7). There was evidence of a nonlinear dose–response relationship;an intake of 4 mg/d was associated with a 22% risk reduction (RR: 0.78; 95% CI: 0.65, 0.94) and no further reduction was evident above this amount (Figure 4). A nonlinear dose–response relationship was also apparent with circulating β-carotene levels; the risk decreased by 44% with levels of 0.5 μmol/L (RR: 0.56; 95% CI: 0.43, 0.73), and levels above this value were associated with moderate additional risk reduction (Figure 4). This relationship was reflected in both high compared with low analyses (RR: 0.61; 95% CI: 0.43, 0.87, *I*^2^ = 61%) (Figure 2 and Supplemental Figure 15) (moderate certainty) and per 1 SD increment (RR: 0.82; 95% CI: 0.70, 0.98, *I*^2^ = 63%) (Supplemental Figure 16) (low certainty), although heterogeneity was substantial and was not explained by sex, geographic region, risk of bias, or smoking prevalence (Supplemental Figures 32–35). Finally, our meta-analysis of RCTs did not suggest a protective effect of β-carotene supplementation on T2D risk (RR: 0.98; 95% CI: 0.90, 1.07, *I*^2^ = 0%) (Figure 2 and Supplemental Figure 17). This finding was of moderate certainty (Figure 5 and Supplemental Table 7).

#### Insulin resistance/sensitivity and β-cell function

There were 3 eligible cohort studies from the United States (*n* = 2) and China (*n* = 1) that examined the relationship between serum β-carotene and insulin resistance (Supplemental Table 5), which because of differences in the exposure/outcome scales were not meta-analyzed. Circulating β-carotene was inversely associated with insulin resistance in the 2 largest studies (Figure 3 and Supplemental References 7 and 29), whereas no statistically significant association was found in the smallest study (Figure 3 and Supplemental Reference 28). Furthermore, a single cohort from Sweden found a positive association between serum β-carotene and insulin sensitivity (Figure 3 and Supplemental Reference 1). Studies investigating the association between β-carotene intake and insulin resistance/sensitivity or β-cell function were not identified.

## Discussion

### Main findings

In our meta-analysis of 25 prospective observational studies, there was evidence of a reduced risk of T2D with higher dietary intakes of vitamin C, vitamin E, and β-carotene that were of moderate to high certainty. The associations appeared to be nonlinear and plateaued at moderate intakes. Results based on circulating levels of these antioxidants aligned with those based on self-reports but were of lower certainty. A protective effect of dietary vitamin C was further supported by inverse associations with insulin resistance, whereas data on β-cell function was scarce. In contrast, there was no support from the 15 included RCTs that supplementation with these antioxidants reduces the risk of T2D, apart from a protective effect of vitamin E supplementation on insulin resistance. These null results were of moderate certainty. A potential explanation for the discrepancy is that antioxidant intakes in the RCT participants were already at moderate levels and the addition of supplements did not add any benefit in terms of T2D prevention.

### Main findings in relation to previous studies

The inverse associations between dietary and circulating antioxidants are in line with previous meta-analyses of vitamin E and β-carotene [11,12], but not with a smaller meta-analysis of vitamin C [11]. This study adds to the body of evidence by showing that these relationships follow a nonlinear dose–response gradient. The lowest diabetes risk was observed at intakes close to the average requirement according to the recent Nordic Nutrition Recommendations and the recommended dietary allowance for vitamins C and E, which are 75 mg/d for women and 90 mg/d for men of vitamin C and 8–15 mg/d of vitamin E [30,31]. As an illustration, these values can be reached by consuming half a red pepper and half a cup of almonds, respectively. For β-carotene, no reference value has been established, but intakes of 3–6 mg/d have been linked to a lower risk of chronic diseases [30]. The findings imply that sufficient rather than high intakes may be important for diabetes prevention. Nevertheless, Mendelian randomization (MR) analyses have failed to confirm a causal relationship between genetically predicted circulating vitamin C, vitamin E, or β-carotene and T2D [[32], [33], [34]]. Although this may indeed indicate a lack of causal relationship, it is likely that the linear MR models failed to capture the hypothesized nonlinear effects of these antioxidants. This is further supported by our synthesis of RCTs, which showed that supplementation of these antioxidants may not confer an additional risk reduction in healthy individuals. Another possible explanation for these null findings is that antioxidants act synergistically in inhibiting oxidative stress [35,36], and thus may offer no clear benefits when received in isolation. Conversely, individuals who incorporate a variety of plant-based foods in their diet can obtain sufficient amounts of these antioxidants and may benefit from their synergistic effects. A novel finding was the lack of association between vitamin C or vitamin E and T2D in smokers. Specifically, inverse associations with vitamins C and E, but not β-carotene, were diluted in studies with higher smoking prevalence. This may be explained by the oxidative stress induced by smoking, which has been linked to reduced circulating vitamins C and E and could counteract their beneficial effects [37,38]. Accordingly, smokers may benefit from higher intakes of these antioxidants and are indeed recommended to consume an additional 35–40 mg/d of vitamin C [30,31], but such a recommendation does not exist for vitamin E.

Beneficial effects of vitamin C, vitamin E, and β-carotene in the prevention of T2D could relate to their unique, yet complementary, antioxidant properties. Vitamin C is a water-soluble vitamin mainly found in fruits and vegetables. Besides its ability to scavenge free radicals in the hydrophilic compartments of the body, vitamin C has been shown to regenerate vitamin E from its oxidized form [39]. On the other hand, vitamin E is a fat-soluble vitamin commonly found in nuts, seeds, and vegetable oils. Accordingly, it protects the lipid phases of the body, such as cell membranes and low-density lipoproteins, from lipid peroxidation [40]. Finally, β-carotene is a provitamin A carotenoid, that is, a fat-soluble pigment found in fruits and vegetables that is converted into vitamin A in the body. As an antioxidant, it is highly effective in preventing different types of free radicals from harming the lipid phases of the body [41]. Altogether, these antioxidants can limit the damaging effects of free radicals, which entail impaired β-cell function and insulin resistance, and can hypothetically prevent T2D. In line with this hypothesis, the findings of this study suggest inverse associations between these antioxidants, particularly vitamin E, and insulin resistance. Studies on β-cell function were scarce and associations were not detected in the individual studies, except for a positive association with dietary vitamin C [42]. Nevertheless, unlike these antioxidants, other antioxidants have been related to an increased risk of T2D when consumed in high amounts. Such an example is selenium, which is suggested to promote insulin resistance because of the excess production of selenoproteins that interfere with insulin signaling [43]. It is therefore crucial to understand the mechanisms and optimal thresholds of different antioxidants.

### Strengths and limitations

To the best of our knowledge, this is the first systematic review and meta-analysis that examines the dose–response relationships of vitamin C and vitamin E with T2D risk, that incorporates RCTs on vitamin C, vitamin E, and β-carotene supplementation, that assesses the outcomes of insulin resistance and β-cell function in relation to these antioxidants, and that investigates whether smoking modifies the association between these antioxidants and T2D incidence. An important strength is the broad literature search that was performed by librarians, which reduced the risk of missing relevant studies. Moreover, this study included a comprehensive risk of bias assessment in eligible studies and an evaluation of the certainty of evidence for all meta-analyses using recommended tools. Notably, only studies with a prospective design were identified and included in meta-analyses, although case–control studies were also eligible. This contributed to a reduced risk of selection and recall bias in most studies. Furthermore, the included studies were representative of different geographic regions, specifically Europe, North America, and Asia.

There are also some limitations that need to be considered in the interpretation of the results. Importantly, the number of studies included in the meta-analyses was small (*n* ≤ 8), which precluded the evaluation of publication bias, as it requires a minimum of 10 studies. Furthermore, subgroup analyses were either not feasible or had low statistical power because of the scarcity of studies. Another limitation is that several observational studies adjusted poorly for other dietary factors or supplementation. Diet is a composite exposure, and it is difficult to isolate the effects of single dietary components, particularly nutrients. For example, people who consume high amounts of vitamin C may also consume high amounts of fiber because both are abundant in fruits and vegetables. Nevertheless, results based on studies that accounted for dietary co-exposures were in line with the main findings. Self-reported diet is susceptible to measurement error. That is especially true for nutrients, which are calculated based on the reported consumption of the overall diet. Nevertheless, data on circulating antioxidants, which provide objective measures of antioxidant exposure, were included, and consistent associations with dietary factors were observed.

### Clinical implication and areas for further research

The findings of this study reinforce the general dietary recommendation of consuming a variety of fruits and vegetables and choosing unsaturated over saturated fats. The use of vitamin C, vitamin E, and β-carotene supplements is not encouraged for the prevention of T2D in healthy individuals with sufficient dietary intakes. Whether the threshold of effectiveness for these antioxidants differs between population subgroups, for example, smokers, those with conditions that limit absorption, or with genetic susceptibility to diabetes, remains to be answered in future studies. Moreover, future RCTs or observational studies that carefully adjust for dietary co-exposures are needed to elucidate if the observed associations reflect the effects of antioxidants or other dietary factors. Finally, there is clearly a need for further studies that examine the mechanisms behind these associations.

### Conclusion

Evidence of moderate to high certainty from observational studies suggests that consuming moderate amounts of vitamin C, vitamin E, and β-carotene may be sufficient to reduce the risk of T2D, with no additional benefit at higher consumption levels. The mechanism may involve improved insulin sensitivity. Evidence from RCTs of moderate certainty indicates that supplementation with these antioxidants does not reduce the risk of T2D, possibly because participants already had adequate intakes. Optimal doses may differ between population subgroups, such as smokers and nonsmokers, and thus require further investigation.

## Author contributions

The authors’ responsibilities were as follows – AML together with SC: conceived the study; AML, TL: independently performed the study selection, data extraction, and risk of bias assessment, and consulted SC in case of disagreement; AML: did the statistical analyses, certainty of evidence assessment, and wrote the first draft with input from SC, JEL, and TL, who also edited the manuscript; and all authors: contributed to the interpretation of the findings, and read and approved the final manuscript.

## Conflict of interest

The authors report no conflicts of interest.

## Funding

The study was supported by the Swedish Research Council for Health, Working Life and Welfare (FORTE, 2018-00337), Novo Nordisk Foundation (NNF19OC0057274), Swedish Research Council (2022-00811), and Swedish Diabetes Foundation (DIA2022-735). The supporting sources had no role in study design; collection, analysis, and interpretation of data; writing of the report; and did not impose any restrictions regarding the publication of the report.

## Data availability

The data described in the manuscript can be provided upon request.
