# Supplementary material for: Vitamins C, E, and β-Carotene and Risk of Type 2 Diabetes: A Systematic Review and Meta-Analysis
Source: Adv Nutr. 2024 Mar 15;15(5):100211. doi: 10.1016/j.advnut.2024.100211 (PMC11002795; doi:10.1016/j.advnut.2024.100211)
Supplement: Multimedia component 1 [file mmc1.pdf]

SUPPLEMENTARY MATERIAL

Vitamins C, E, and beta-carotene and risk of type 2 diabetes: a systematic review and meta-analysis

Authors:
Anna-Maria Lampousi, Therese Lundberg, Josefin E. Löfvenborg and Sofia Carlsson

Correspondence to:
Anna-Maria Lampousi
Institute of Environmental Medicine, Karolinska Institutet
Box 210, 171 77 Stockholm, Sweden
+46702575629
annamaria.lampousi@ki.se

Table of Contents

Supplementary Table 1. Search strategy for identification of relevant articles in Medline 4

Supplementary Table 2. Search strategy for identification of relevant articles in Embase 6

Supplementary Table 3. Search strategy for identification of relevant articles in Cochrane Library 8

Supplementary Table 4. Studies that were excluded after full text screening and reasons for exclusion 9

Supplementary Table 5. Characteristics of eligible studies on type 2 diabetes 10

Supplementary Table 6. Characteristics of eligible studies on insulin resistance, insulin sensitivity, and beta cell function 19

Supplementary Table 7. Certainty of evidence assessment using the GRADE system 22

Supplementary Figure 1. Risk of bias assessment of observational studies using ROBINS-I, generated with the robvis tool 25

Supplementary Figure 2. Risk of bias assessment of randomized controlled trials using RoB 2.0, generated with the robvis tool 26

Supplementary Figure 3. Summary of relative risk of type 2 diabetes for high vs low dietary vitamin C 27

Supplementary Figure 4. Summary of relative risk of type 2 diabetes per 10 mg/day increment in dietary vitamin C 28

Supplementary Figure 5. Summary of relative risk of insulin resistance for high vs low dietary vitamin C 29

Supplementary Figure 6. Summary of relative risk of type 2 diabetes for high vs low dietary vitamin E 30

Supplementary Figure 7. Summary of relative risk of type 2 diabetes per 1 mg/day increment in dietary vitamin E 31

Supplementary Figure 8. Summary of relative risk of insulin resistance for high vs low dietary vitamin E 32

Supplementary Figure 9. Summary of relative risk of type 2 diabetes for high vs low circulating vitamin E (alpha-tocopherol) 33

Supplementary Figure 10. Summary of relative risk of type 2 diabetes per 1 standard deviation increment in circulating vitamin E (alpha-tocopherol) 34

Supplementary Figure 11. Summary of relative risk of type 2 diabetes for vitamin E (alpha-tocopherol) supplementation vs placebo 35

Supplementary Figure 12. Summary of mean difference of the change in HOMA-IR from baseline between individuals using vitamin E supplements or placebo/lifestyle intervention 36

Supplementary Figure 13. Summary of relative risk of type 2 diabetes for high vs low dietary beta-carotene 37

Supplementary Figure 14. Summary of relative risk of type 2 diabetes per 1 mg/day increment in dietary beta-carotene 38

Supplementary Figure 15. Summary of relative risk of type 2 diabetes for high vs low circulating beta-carotene 39

Supplementary Figure 16. Summary of relative risk of type 2 diabetes per 1 standard deviation increment in circulating beta-carotene 40

Supplementary Figure 17. Summary of relative risk of type 2 diabetes for beta-carotene supplementation vs placebo 41

Supplementary Figure 18. Meta-regression for high vs low dietary vitamin C and type 2 diabetes with smoking prevalence within the cohort as effect modifier (p=0.081) 42

Supplementary Figure 19. Subgroup analysis for high vs low dietary vitamin C and type 2 diabetes based on sex 43

Supplementary Figure 20. Subgroup analysis for high vs low dietary vitamin C and type 2 diabetes based on geographic region 44

Supplementary Figure 21. Sensitivity analysis for high vs low dietary vitamin C and type 2 diabetes including only studies that had adjusted for dietary co-exposures CI, confidence interval; T2D, type 2 diabetes 45

Supplementary Figure 22. Sensitivity analysis for high vs low dietary vitamin E and type 2 diabetes including only studies that had adjusted for dietary co-exposures 46

Supplementary Figure 23. Meta-regression for high vs low circulating vitamin E (alpha-tocopherol) and type 2 diabetes with smoking prevalence within the cohort as effect modifier (p=0.007) 47

Supplementary Figure 24. Subgroup analysis for high vs low circulating vitamin E (alpha-tocopherol) and type 2 diabetes based on sex 48

Supplementary Figure 25. Subgroup analysis for high vs low circulating vitamin E (alpha-tocopherol) and type 2 diabetes based on geographic region 49

Supplementary Figure 26. Subgroup analysis for high vs low circulating vitamin E (alpha-tocopherol) and type 2 diabetes based on risk of bias 50

Supplementary Figure 27. Subgroup analysis for vitamin E supplementation vs placebo or lifestyle intervention and HOMA-IR based on age group 51

Supplementary Figure 28. Subgroup analysis for vitamin E supplementation vs placebo or lifestyle intervention and HOMA-IR based on sex 52

Supplementary Figure 29. Subgroup analysis for vitamin E supplementation vs placebo or lifestyle intervention and HOMA-IR based on risk of bias 53

Supplementary Figure 30. Subgroup analysis for vitamin E supplementation vs placebo or lifestyle intervention and HOMA-IR based on geographic region 54

Supplementary Figure 31. Subgroup analysis for vitamin E supplementation vs placebo or lifestyle intervention and HOMA-IR based on health condition 55

Supplementary Figure 32. Meta-regression for high vs low circulating beta-carotene and type 2 diabetes with smoking prevalence within the cohort as effect modifier (p=0.750) 56

Supplementary Figure 33. Subgroup analysis for high vs low circulating beta-carotene and type 2 diabetes based on sex 57

Supplementary Figure 34. Subgroup analysis for high vs low circulating beta-carotene and type 2 diabetes based on geographic region 58

Supplementary Figure 35. Subgroup analysis for high vs low circulating beta-carotene and type 2 diabetes based on risk of bias 59

Supplementary references 60

Supplementary Table 1. Search strategy for identification of relevant articles in Medline

Interface: Ovid MEDLINE(R) ALL<br>Date of Search: 23 May 2023<br>Number of hits: 2,871<br>Comment: In Ovid, two or more words are automatically searched as phrases; i.e. no quotation marks are needed	Field labels<br>• exp/ = exploded MeSH term<br>• / = non exploded MeSH term<br>• .ti,ab,kf. = title, abstract and author keywords<br>• adjx = within x words, regardless of order<br>• * = truncation of word for alternate endings

Database(s): **Ovid MEDLINE(R) ALL** 1946 to May 22, 2023
Search Strategy:

#	Searches	Results
1	Diabetes Mellitus, Type 2/	169495
2	Insulin Resistance/	67012
3	Insulin Secretion/	23794
4	(diabet* adj2 (maturity-onset or type II or type 2)).ti,ab,kf.	189692
5	(diabetes mellitus adj1 (adult-onset or ketosis-resistant or noninsulin-dependent or non insulin dependent or slow-onset or stable)).ti,ab,kf.	7945
6	NIDDM.ti,ab,kf.	6975
7	(insulin adj1 (resistan* or sensitiv*)).ti,ab,kf.	120010
8	(insulin secretion or insulin deficien* or insulin release).ti,ab,kf.	42191
9	((beta-cell adj2 funct*) or HOMA or hom?eostasis model assessment or hom?eostatic model assessment).ti,ab,kf.	35243
10	or/1-9	363108
11	exp Ascorbic Acid/	45312
12	beta Carotene/	8324
13	Carotenoids/	21192
14	exp Vitamin E/	34660
15	((ascorbic or dehydroascorbic) adj1 acid*).ti,ab,kf.	38258
16	((ferrous or magnesium or sodium) adj1 ascorb*).ti,ab,kf.	1244
17	(c-vitamin* or hybrin or magnorbin or vitamin* c).ti,ab,kf.	29311
18	(bellacarotin or betacarotene or beta-carotene or carotaben or max-caro or maxcaro or provatene or solatene or vetoron).ti,ab,kf.	16049
19	(carotenoid? or caroten tetraterpene* or carotene? or tetraterpene derivatives).ti,ab,kf.	39589
20	(alpha tocopherol or vitamin* E or E vitamin*).ti,ab,kf.	43579
21	((almond* or apricot? or asparagus or avocado* or blackcurrant? or broccoli or brussels or carrot? or cereal? or citrus or fruit? or mango? or nut or nuts or orange? or papaya? or peanut* or pepper? or peppers or potato? or pumpkin or seed or seeds or spinach or strawberry* or vegetable? or wheatgerm*) adj3 (ate or consum* or diet* or eat or eating or intake* or ingest*)).ti,ab,kf.	37270
22	or/11-21	187619
23	Antioxidants/	151132
24	Micronutrients/	7561
25	Nutrients/	6889
26	Vitamins/	36406
27	or/24-26	49751
28	23 and 27	2984
29	((antioxid* or anti-oxid*) adj2 (micronutri* or nutrition* or nutrient* or trace element? or vitamin*)).ti,ab,kf.	8112
30	22 or 28 or 29	190935
31	Diet/	185149
32	Dietary Supplements/	73464
33	Plasma/	24783

34	Serum/	11398
35	(ate or consum* or diet* or eat or eating or intake* or ingest).ti,ab,kf.	1396688
36	(nutr?ceutical* or supplement*).ti,ab,kf.	426411
37	(blood or plasma* or serum).ti,ab,kf.	3796291
38	or/31-37	5149914
39	administration dosage.fs.	1497731
40	blood.fs.	1841736
41	pharmacology.fs.	3358578
42	"therapeutic use".fs.	2495128
43	(administration or dosage).ti,ab,kf.	1089317
44	("mode of action" or "mechanism of action" or pharmacolog* or pharmacodynamic*).ti,ab,kf.	618791
45	"therapeutic use".ti,ab,kf.	72618
46	or/39-45	8245835
47	38 or 46	10996783
48	30 and 47	140438
49	10 and 48	3519
50	49 not (animals not humans).sh.	3053
51	limit 50 to (clinical conference or comment or congress or consensus development conference or consensus development conference, nih or editorial or letter)	47
52	50 not 51	3006
53	limit 52 to english language	2871

Supplementary Table 2. Search strategy for identification of relevant articles in Embase

Interface: embase.com<br>Date of Search: 23 May 2022<br>Number of hits: 4,258<br>Comment: Emtree is the controlled vocabulary in Embase	Field labels<br>• /exp = exploded Emtree term<br>• /de = non exploded Emtree term<br>• ti,ab,kw = title, abstract and author keywords<br>• NEAR/x = within x words, regardless of order<br>• * = truncation of word for alternate endings

No.	Query	Results
#1	'insulin resistance'/de	144295
#2	'insulin release'/de	53458
#3	'non insulin dependent diabetes mellitus'/de	329781
#4	(diabet* NEAR/2 ('maturity-onset' OR 'type ii' OR 'type 2')):ti,ab,kw	291074
#5	('diabetes mellitus' NEAR/1 ('adult-onset' OR 'ketosis-resistant' OR 'noninsulin-dependent' OR 'non insulin dependent' OR 'slow-onset' OR stable)):ti,ab,kw	9609
#6	niddm:ti,ab,kw	8300
#7	(insulin NEAR/1 (resistan* OR sensitiv*)):ti,ab,kw	174958
#8	'insulin secretion':ti,ab,kw OR 'insulin deficien*':ti,ab,kw OR 'insulin release*':ti,ab,kw	58497
#9	(('beta cell' NEAR/2 funct*):ti,ab,kw) OR homa:ti,ab,kw OR 'hom$eostasis model assessment':ti,ab,kw OR 'hom$eostatic model assessment':ti,ab,kw	47288
#10	#1 OR #2 OR #3 OR #4 OR #5 OR #6 OR #7 OR #8 OR #9	574441
#11	'ascorbic acid'/exp	116294
#12	'beta carotene'/de	22700
#13	'carotenoid'/de	28159
#14	'alpha tocopherol'/de	79017
#15	((ascorbic OR dehydroascorbic) NEAR/1 acid*):ti,ab,kw	45522
#16	((ferrous OR magnesium OR sodium) NEAR/1 ascorb*):ti,ab,kw	1441
#17	'c-vitamin*':ti,ab,kw OR hybrin:ti,ab,kw OR magnorbin:ti,ab,kw OR 'vitamin* c':ti,ab,kw	35386
#18	bellacarotin:ti,ab,kw OR 'β-caroten':ti,ab,kw OR betacarotene:ti,ab,kw OR 'beta-carotene':ti,ab,kw OR carotaben:ti,ab,kw OR 'max-caro':ti,ab,kw OR maxcaro:ti,ab,kw OR provatene:ti,ab,kw OR solatene:ti,ab,kw OR vetoron:ti,ab,kw	5076
#19	carotenoid$:ti,ab,kw OR 'caroten tetraterpene*':ti,ab,kw OR carotene$:ti,ab,kw OR 'tetraterpene derivatives':ti,ab,kw	43277
#20	'alpha tocopherol':ti,ab,kw OR 'vitamin* e':ti,ab,kw OR 'e-vitamin*':ti,ab,kw	42899
#21	((almond* OR apricot$ OR asparagus OR avocado* OR blackcurrant$ OR broccoli OR brussels OR carrot$ OR cereal$ OR citrus OR fruit$ OR mango$ OR nut OR nuts OR orange$ OR papaya$ OR peanut* OR pepper$ OR peppers OR potato$ OR pumpkin OR seed OR seeds OR spinach OR strawberry* OR vegetable$ OR wheatgerm*) NEAR/3 (ate OR consum* OR diet* OR eat OR eating OR intake* OR ingest*)):ti,ab,kw	46605
#22	#11 OR #12 OR #13 OR #14 OR #15 OR #16 OR #17 OR #18 OR #19 OR #20 OR #21	274528
#23	'antioxidant'/de	180896
#24	'nutrient'/de	47983
#25	'trace element'/de	42309
#26	'vitamin'/de	51004
#27	#24 OR #25 OR #26	133791
#28	#23 AND #27	7202
#29	((antioxid* OR 'anti-oxid*') NEAR/2 (micronutri* OR nutrition* OR nutrient* OR 'trace element*' OR vitamin*)):ti,ab,kw	10237
#30	#22 OR #28 OR #29	280002
#31	'blood level'/de	121600

#32	'diet'/de	265604
#33	'dietary supplement'/de	22375
#34	'plasma'/de	168373
#35	'serum'/de	198704
#36	ate:ti,ab,kw OR consum*:ti,ab,kw OR diet*:ti,ab,kw OR eat*:ti,ab,kw OR eating:ti,ab,kw OR intake*:ti,ab,kw OR ingest*:ti,ab,kw	1886745
#37	nutr$ceutical*:ti,ab,kw OR supplement*:ti,ab,kw	539170
#38	blood:ti,ab,kw OR plasma*:ti,ab,kw OR serum:ti,ab,kw	5122137
#39	#31 OR #32 OR #33 OR #34 OR #35 OR #36 OR #37 OR #38	6908541
#40	administration:ti,ab,kw OR dosage:ti,ab,kw	1448277
#41	'mode of action':ti,ab,kw OR 'mechanism of action':ti,ab,kw OR pharmacolog*:ti,ab,kw OR pharmacodynamic*:ti,ab,kw	797427
#42	#40 OR #41	2145067
#43	#39 OR #42	8352224
#44	#30 AND #43	150826
#45	#10 AND #44	6340
#46	#45 NOT ([animals]/lim NOT [humans]/lim)	5588
#47	#46 AND ('conference abstract'/it OR 'conference paper'/it OR 'conference review'/it OR 'editorial'/it OR 'letter'/it OR 'note'/it)	1086
#48	#46 NOT #47	4502
#49	#46 NOT #47 AND [english]/lim	4258

Supplementary Table 3. Search strategy for identification of relevant articles in Cochrane Library

Interface: Wiley<br>Date of Search: 23 May 2023<br>Number of hits: 1,954	Field labels<br>• ti,ab,kw = title, abstract and author keywords<br>• NEAR/x = within x words, regardless of order<br>• * = truncation of word for alternate endings

ID	Search	Hits
#1	[mh ^"Diabetes Mellitus, Type 2"]	22982
#2	[mh ^"Insulin Resistance"]	6717
#3	[mh ^"Insulin Secretion"]	757
#4	(diabet* NEAR/2 ("maturity-onset" OR "type II" OR "type 2")):ti,ab,kw	57025
#5	("diabetes mellitus" NEAR/1 ("adult-onset" OR "ketosis-resistant" OR "noninsulin-dependent" OR "non insulin dependent" OR "slow-onset" OR stable)):ti,ab,kw	80259
#6	NIDDM:ti,ab,kw	1124
#7	(insulin NEAR/1 (resistan* OR sensitiv*)):ti,ab,kw	19085
#8	("insulin secretion" OR (insulin NEXT deficien*) or "insulin release"):ti,ab,kw	3878
#9	((("beta cell" OR "β-cell") NEAR/2 funct*) OR HOMA OR "hom?eostasis model assessment" OR "hom?eostatic model assessment"):ti,ab,kw	8393
#10	#1 OR #2 OR #3 OR #4 OR #5 OR #6 OR #7 OR #8 OR #9	130291
#11	[mh "Ascorbic Acid"]	2573
#12	[mh ^"beta Carotene"]	881
#13	[mh ^Carotenoids]	868
#14	[mh "Vitamin E"]	2864
#15	((ascorbic OR dehydroascorbic) NEAR/1 acid*):ti,ab,kw	4250
#16	((ferrous OR magnesium OR sodium) NEAR/1 ascorb*):ti,ab,kw	137
#17	((c NEXT vitamin*) OR hybrin OR magnorbin OR (vitamin* NEXT c)):ti,ab,kw	4399
#18	(bellacarotin OR "β-caroten" OR betacarotene OR "beta-carotene" OR carotaben OR "max-caro" OR maxcaro OR provatene OR solatene OR vetoron):ti,ab,kw	1864
#19	(carotenoid? OR (caroten NEXT tetraterpene*) OR carotene? OR "tetraterpene derivatives"):ti,ab,kw	3098
#20	((vitamin* NEXT E) OR (E NEXT vitamin*)):ti,ab,kw	5292
#21	alpha tocopherol:ti,ab,kw	2472
#22	((almond* or apricot? or asparagus or avocado* or blackcurrant? or broccoli or brussels or carrot? or cereal? or citrus or fruit? or mango? or nut or nuts or orange? or papaya? or peanut* or pepper? or peppers or potato? or pumpkin or seed or seeds or spinach or strawberry* or vegetable? or wheatgerm*) NEAR/3 (ate or consum* or diet* or eat or eating or intake* or ingest*)):ti,ab,kw	7001
#23	#11 OR #12 OR #13 OR #14 OR #15 OR #16 OR #17 OR #18 OR #19 OR #20 OR #21 OR #22	19328
#24	[mh ^Antioxidants]	5402
#25	[mh ^Micronutrients]	1154
#26	[mh ^Nutrients]	257
#27	[mh ^Vitamins]	4764
#28	#25 OR #26 OR #27	6018
#29	#24 AND #28	410
#30	((antioxid* OR (anti NEXT oxid*)) NEAR/2 (micronutri* OR nutrition* OR nutrient* OR (trace NEXT element?) OR vitamin*)):ti,ab,kw	1338
#31	#23 OR #29 OR #30	19759
#32	[mh ^Diet]	11235
#33	[mh ^"Dietary Supplements"]	13902
#34	[mh ^Plasma]	2913

#35	[mh ^Serum]	2437
#36	(ate OR consum* OR diet* OR eat OR eating OR intake* OR ingest*):ti,ab,kw	211486
#37	(nutr?ceutical* or supplement*):ti,ab,kw	84996
#38	(blood OR plasma* OR serum):ti,ab,kw	494811
#39	[mh /AD]	195611
#40	[mh /BL]	116016
#41	[mh /PD]	76972
#42	[mh /TU]	247627
#43	(administration OR dosage):ti,ab,kw	410993
#44	("mode of action" OR "mechanism of action" OR pharmacolog* OR pharmacodynamic*):ti,ab,kw	138006
#45	therapeutic use:ti,ab,kw	278239
#46	#32 OR #33 OR #34 OR #35 OR #36 OR #37 OR #38 OR #39 OR #40 OR #41 OR #42 OR #43 OR #44 OR #45	977160
#47	#31 AND #46	17965
#48	#47 AND #10	2017
#49	#48 in Trials	2000
#50	#49 in English	1954

Supplementary Table 4. Studies that were excluded after full text screening and reasons for exclusion

Reason	Number of studies	References
Not relevant study design	79	39-117
Not relevant exposure	244	118-361
Not relevant outcome	20	362-381
Not relevant publication type	37	382-418
Prevalent diabetes	19	419-437
Estimates not reported	3	438-440
Not in English	3	441-442
Animal study	1	444
Retracted	1	445

Supplementary Table 5. Characteristics of eligible studies on type 2 diabetes

Study	Country	Study name	Study design	N total	N cases	Sex	Age, years	Exposure	Exposure assessment	T2D assessment	RR (95% CI)	Adjusted factors
Ärnlöv 2009[1]	Sweden	ULSAM	Cohort	684	57	Men	70	Dietary alpha-tocopherol: >6.30 vs <4.82 mg/day	7-day pre-coded food diary	WHO criteria from 1999 and Swedish national hospital discharge register	0.70 (0.31, 1.58)	BMI, level of physical activity, smoking status, impaired fasting glucose, insulin sensitivity [clamp], and early insulin response in the OGTT
								Dietary beta-carotene: >1.89 vs <1.02 mg/day			0.67 (0.31, 1.44)	
				846	245		50	Serum alpha-tocopherol: >3.67 vs <3.25 µmol/mmol	HPLC		0.61 (0.34, 1.07)	BMI, level of physical activity, smoking status, fasting glucose, insulin sensitivity [HOMA], and acute insulin response at IVGTT
								Serum beta-carotene: >0.335 vs <0.210 µmol/L			0.41 (0.23, 0.74)	
Cooper 2015[2]	England and Wales	EPIC-Norfolk	Nested case-control	1244	318	Men and women	40-79	Plasma beta-carotene: 40.8 vs 8.9 µg/dL	HPLC	Self-report and linkage with general practice and hospital diabetes registers, hospital admissions data and mortality data	0.35 (0.21, 0.58)	Age, sex, education level, occupational social class, smoking status, physical activity level, family history of diabetes, total energy intake, vitamin supplement use, HDL- and LDL cholesterol, BMI and waist circumference
								Plasma vitamin C: 74.3 vs 25.6 µmol/L	Fluorometric assay		0.47 (0.29, 0.75)	
de Oliveira Otto 2012[3]	USA	MESA	Cohort	4982	399	Men and women	45-84	Dietary vitamin C: ≥136 vs ≤57 mg/day	FFQ	Self-report, serum glucose ≥ 6.99 mmol/L, or new use of hypoglycemic medication	0.91 (0.63, 1.32)	Energy intake, age, sex, race-ethnicity, study center, alcohol intake, physical activity, BMI, fiber intake, cigarette smoking, dietary supplement use, ratio of polyunsaturated fat intake to saturated fat intake and mutual adjustment for Mg,
								Dietary vitamin E: ≥12.4 vs ≤7.3 mg/day			0.82 (0.53, 1.27)	

Study	Country	Study name	Study design	N total	N cases	Sex	Age, years	Exposure	Exposure assessment	T2D assessment	RR (95% CI)	Adjusted factors
								Dietary beta-carotene: ≥4.75 vs ≤1.61 mg/day			0.81 (0.54, 1.21)	Zn, heme iron, non-heme iron and antioxidants intake
Eshak 2019a[4]	Japan	Japan Collaborative Cohort Study for Evaluation of Cancer Risk	Cohort	19168	494	Men and women	40-79	Dietary vitamin C: 196.9 vs 90.1 mg/day	FFQ	Self-reported diagnosis	0.73 (0.55, 0.96)	Age, past history of hypertension, family history of diabetes, BMI, smoking status, alcohol intakes, hours of exercise, hours of walking, supplement use of vitamins E, C, B1 and multivitamins, intakes of coffee and green tea and quartiles of total energy and Mg intakes, quartiles of fat-soluble vitamins (A, K, E, D) intakes
Eshak 2019b[5]	Japan	Japan Collaborative Cohort Study for Evaluation of Cancer Risk	Cohort	19168	494	Men and women	40-79	Dietary vitamin E: 7.5 vs 3.1 mg/day	FFQ	Self-reported diagnosis	0.77 (0.59, 0.98)	Age, sex, past history of hypertension, family history of diabetes, BMI, smoking status, hours of exercise, hours of walking and supplement use of vitamins E and C and multivitamins coffee, green tea and alcohol intakes and total energy, carbohydrate and Mg intakes, dietary intakes of fat-soluble vitamins
Harding 2008[6]	England and Wales	EPIC-Norfolk	Cohort	19246	638	Men and women	40-75	Plasma vitamin C: ≥1.20 vs ≤0.7 mg/dL	Fluorometric assay	Self-report and linkage with general practice and hospital diabetes registers, hospital admissions	0.38 (0.28, 0.52)	Age, sex, family history of diabetes, alcohol consumption, educational level, occupational socioeconomic class, physical activity, smoking status, vitamin supplementation, BMI, and waist circumference

Study	Country	Study name	Study design	N total	N cases	Sex	Age, years	Exposure	Exposure assessment	T2D assessment	RR (95% CI)	Adjusted factors
										data and mortality data		
Hozawa 2006[7]	USA	CARDIA	Cohort	4681	148	Men and women	18-30	Serum beta-carotene: per 1 SD increase	HPLC	Fasting glucose level of at least 126 mg/dl at year 7, 10, or 15 or the self-reported use of oral hypoglycemic medication or insulin at any examination	0.89 (0.69, 1.16)	Race, sex, study center, age, education, systolic blood pressure, ethanol intake, plasma levels of total cholesterol, high density lipoprotein cholesterol, and triglycerides, total energy intake, BMI, total energy expenditure, and use of vitamin supplements (vitamin A, C, or E or b-carotene).
Kataja-Tuomola 2011[8]	Finland	ATBC	Cohort	25505	660	Men	50-69	Dietary vitamin C: 152.83 vs 54.24 mg/day	FFQ	Social insurance register	1.04 (0.81, 1.33)	Age, supplementation, BMI, cigarettes smoked daily, smoking years, blood pressure, total cholesterol, high-density lipoprotein cholesterol, leisure-time physical activity and daily intake of alcohol and energy
								Dietary alpha-tocopherol: 15.47 vs 6.09 mg/day			0.92 (0.71, 1.19)	
								Dietary beta-carotene: 3.9 vs 0.77 mg/day			1.03 (0.79, 1.35)	
Kataja-Tuomola 2008[9]	Finland	ATBC	Cohort	6867	188	Men	50-69	Serum alpha-tocopherol: 15.7 vs 8.3 mg/L	HPLC	Social insurance register or self-reported diabetes medication or physician-diagnosed,	1.59 (0.89, 2.84)	Age, BMI, number of cigarettes per day, years of smoking, total cholesterol, HDL-cholesterol, systolic and diastolic blood pressures, alcohol consumption and leisure-time physical activity
								Serum beta-carotene: 379 vs 72 µg/L			0.66 (0.40, 1.10)	

Study	Country	Study name	Study design	N total	N cases	Sex	Age, years	Exposure	Exposure assessment	T2D assessment	RR (95% CI)	Adjusted factors
										diet-treated diabetes		
			RCT	13729	358			Alpha-tocopherol supplementation: 50 mg/day vs placebo for 6 years			0.91 (0.73, 1.12)	
				13699	368			Beta-carotene supplementation: 20 mg/day vs placebo for 6 years			0.97 (0.79, 1.20)	
Klein 2011[10] Dunn 2010[11] Lippman 2009[12]	USA, Canada, Puerto Rico	SELECT	RCT	17433	1369	Men	≥50	Alpha-tocopherol supplementation: 400 mg/day vs placebo for 7-12 years		Self-report or reported use of diabetes medication	1.04 (0.94, 1.14)	
Liu 1999[13]	USA	Physician's Health Study	RCT	21468	798	Men	40-84	Beta-carotene supplementation: 50 mg every other day vs placebo for 12 years		Self-report	0.99 (0.86, 1.14)	Age, aspirin assignment, smoking status, alcohol intake, physical activity, BMI, history of high cholesterol or hypertension, use of multivitamins

Study	Country	Study name	Study design	N total	N cases	Sex	Age, years	Exposure	Exposure assessment	T2D assessment	RR (95% CI)	Adjusted factors
Liu 2006[14]	USA	WHS	RCT	38716	1696	Women	≥45	Alpha-tocopherol supplementation: 600 IU every other day vs placebo for 10 years		Self-report confirmed by a medical record review	0.95 (0.87, 1.05)	
Mayer-Davis 2002[15]	USA	IRAS	Cohort	895	148	Men and women	40-69	Dietary vitamin E: highest vs lowest quintile of alpha-tocopherol equivalents	FFQ	WHO criteria or report taking oral hypoglycemic agents	0.74 (0.24, 2.25)	Glucose tolerance status at baseline, age, ethnicity, clinic, sex, general health, family history of diabetes, calories, BMI, waist circumference, smoking status, participation in vigorous physical activity, total fat intake, fiber intake, alcohol intake, and intake of Mg and vitamin C from food and supplements
								Plasma alpha-tocopherol: ≥40 vs <24 µmol/L	HPLC		0.27 (0.06, 1.17)	
Montonen 2004[16]	Finland	Finnish Mobile Clinic Health Examination Survey	Cohort	3921	383	Men and women	40-69	Dietary vitamin C: >87.9 vs <49.7 mg/day	Dietary history interview	Social insurance register	0.97 (0.72, 1.32)	Age, sex, geographic area, occupation, smoking, BMI, and family history of diabetes, and energy intake
								Dietary vitamin E: >7.31 vs <5.51 alpha-tocopherol equivalents mg/day			0.69 (0.51, 0.94)	

Study	Country	Study name	Study design	N total	N cases	Sex	Age, years	Exposure	Exposure assessment	T2D assessment	RR (95% CI)	Adjusted factors
								Dietary beta-carotene: >2121 vs <698 µg/day			0.74 (0.54, 1.01)	
Prentice 2019[17]	USA	WHI	Cohort	3693	644	Women	50-79	Dietary alpha-tocopherol: per doubling in intake	Estimated intake based on serum levels	Self-reported taking oral diabetes medications or insulin	1.05 (0.98, 1.13)	Age, race/ethnicity, family income, education, cigarette smoking history, alcohol consumption, leisure physical activity, height, weight, any supplement use, prior menopausal hormone use, hypertension, personal history of cancer, and personal or family history of diabetes
								Dietary beta-carotene: per doubling in intake			0.72 (0.63, 0.82)	
Reunanen 1998[18]	Finland	Mobile Clinic Health Examination of the Social Insurance Institution	Nested case-control	307	106	Men and women	15-99	Serum alpha-tocopherol: highest vs lowest tertile	HPLC	Social insurance register	0.78 (0.28, 2.2)	Smoking, hypertension serum cholesterol and BMI, plasma glucose
								Serum beta-carotene: highest vs lowest tertile			1.53 (0.51, 4.64)	
Salonen 1995[19]	Finland	Kuopio ischaemic heart disease risk factor study	Cohort	944	45	Men	42-60	Plasma alpha-tocopherol: ≥0.98 vs <0.98 µmol/L	HPLC	WHO criteria complemented by a clinical diagnosis	0.20 (0.08, 0.50)	Age, socioeconomic status, BMI, cigarettes smoked daily, ratio of serum saturated fatty acids to sum of monoenes and polyenes, baseline blood glucose and serum fructosamine concentrations
Savolainen 2017[20]	Sweden		Cohort	399	69	Women	64	Plasma alpha-tocopherol: per 1 unit increase		WHO criteria	0.72 (0.56, 0.93)	Total cholesterol, LDL
Sluijs 2015[21]	the Netherlands	EPIC-NL	Cohort	37846	915	Men and women	21-70	Dietary beta-carotene: 3.7 vs 1.3 mg/day	FFQ	Self-report and linkage with hospital	0.78 (0.64, 0.95)	Age, sex, educational status, physical activity, systolic blood pressure,

Study	Country	Study name	Study design	N total	N cases	Sex	Age, years	Exposure	Exposure assessment	T2D assessment	RR (95% CI)	Adjusted factors
										discharge diagnoses registries. Verified against general practitioner or pharmacist information.		fiber intake, vitamin E intake, BMI, and waist circumference
Song 2009[22]	USA	Women's Antioxidant Cardiovascular Study	RCT	6574	895	Women	≥40	Alpha-tocopherol supplementation: 600 IU every other day vs placebo for 9 years		Self-reported confirmed by a medical record review	1.13 (0.99, 1.29)	
								Beta-carotene supplementation: 50 mg every other day vs placebo for 9 years			0.97 (0.85, 1.11)	
								Ascorbic acid supplementation: 500 mg/day vs placebo for 9 years			0.89 (0.78, 1.02)	
Sugiura 2015[23]	Japan	Mikkabi Cohort Study	Cohort	864	55	Men and women	30-79	Serum beta-carotene: 1.22 vs 0.32 mmol/L	HPLC	Fasting plasma glucose was equal to or more than 7 mmol/L or treatment with oral hypoglycemic medication or insulin	0.53 (0.23, 1.22)	Age, sex, BMI, current tobacco use, exercise habits, total energy intake excluding ethanol, and ethanol intake
Wang 2006[24]	USA	WHS	Nested case-control	940	470	Women	≥45	Plasma beta-carotene: 48.7 vs 9.1 μg/dL	HPLC	Self-reported	1.1 (0.57, 2.13)	Smoking status, alcohol use, exercise, menopausal status, postmenopausal hormone use, multivitamin use, family history of diabetes, BMI, history of

Study	Country	Study name	Study design	N total	N cases	Sex	Age, years	Exposure	Exposure assessment	T2D assessment	RR (95% CI)	Adjusted factors
												hypertension, and hypercholesterolemia, total energy intake, energy-adjusted intake of saturated fat, fiber, and dietary glycemic load
Zheng 2020[25]	Denmark, France, Germany, Italy, Netherlands, Spain, Sweden, and UK	EPIC-InterAct	Case-cohort	22833	9754	Men and women	≥20	Plasma beta-carotene: 0.87 vs 0.14 µmol/L	HPLC	Self-report and linkage to primary and secondary care registers, use of medication (drug registers), hospital admissions, or mortality data	0.45 (0.39, 0.52)	Age, sex, centre, physical activity, smoking status, employment, marital status, education, alcohol intake, total energy intake, high density lipoprotein cholesterol, and low-density lipoprotein cholesterol BMI, and waist circumference
								Plasma vitamin C: 64.9 vs 17 µmol/L			0.58 (0.47, 0.72)	Age, sex, centre, physical activity, smoking status, employment, marital status, education, alcohol intake, total energy intake, BMI, and waist circumference
Zheng 2021[26]	Denmark, France, Germany, Italy, Netherlands,	EPIC-InterAct	Case-cohort	19206	8133	Men and women	≥20	Plasma vitamin C: per 1 SD increase	HPLC	Self-report and linkage to primary and secondary care registers, use of medication	0.88 (0.82, 0.94)	Age, sex, centre, physical activity, smoking status, employment, marital status, education, alcohol intake, total energy intake, individual plasma

Study	Country	Study name	Study design	N total	N cases	Sex	Age, years	Exposure	Exposure assessment	T2D assessment	RR (95% CI)	Adjusted factors
	Spain, Sweden, and UK									(drug registers), hospital admissions, or mortality data		carotenoids, BMI, and waist circumference
Zhou 2016[27]	China	HDNNC DS	Cohort	7595	522	Men and women	20-74	Dietary vitamin C: ≥109.93 vs <67.56 mg/day	FFQ	Based on OGTT, diabetes was defined as fasting blood glucose ≥7.0 mmol/L, and/or 2-glucose ≥11.1 mmol/L	0.76 (0.64, 0.89)	Age at study recruitment, sex, BMI, waist circumference, exercise regularly, total energy intake, hypertension, coronary heart disease, hyperlipemia, body fat percentage, education, current smoking, and family history of type 2 diabetes
Zhou 2016[27]	China	HDNNC DS	Cohort	7595	522	Men and women	20-74	Dietary vitamin E: ≥13.49 vs <9.67 mg/day	FFQ	Based on OGTT, diabetes was defined as fasting blood glucose ≥7.0 mmol/L, and/or 2-glucose ≥11.1 mmol/L	0.89 (0.65, 1.2)	Age at study recruitment, sex, BMI, waist circumference, exercise regularly, total energy intake, hypertension, coronary heart disease, hyperlipemia, body fat percentage, education, current smoking, and family history of type 2 diabetes
Zhou 2016[27]	China	HPHS	Cohort	3483	178	Men and women	20-74	Dietary vitamin C: ≥116.6 vs <69.08 mg/day	FFQ	Based on OGTT, diabetes was defined as fasting blood glucose ≥7.0 mmol/L, and/or 2-glucose ≥11.1 mmol/L, and/or taking medications for diabetes in the follow-up data in the HPHS	0.46 (0.3, 0.71)	Age at study recruitment, sex, BMI, waist circumference, exercise regularly, total energy intake, hypertension, coronary heart disease, and hyperlipemia
Zhou 2016[27]	China	HPHS	Cohort	3483	178	Men and women	20-74	Dietary vitamin E: ≥12.78 vs <9.07 mg/day	FFQ	Based on OGTT, diabetes was defined as fasting blood glucose ≥7.0 mmol/L, and/or 2-glucose ≥11.1 mmol/L, and/or taking medications for diabetes in the follow-up data in the HPHS	0.71 (0.39, 1.28)	Age at study recruitment, sex, BMI, waist circumference, exercise regularly, total energy intake, hypertension, coronary heart disease, and hyperlipemia

ATBC, Alpha-Tocopherol, Beta-Carotene Cancer Prevention; BMI, body mass index; CARDIA, Coronary Artery Risk Development in Young Adults; CI, confidence interval; EPIC, European Prospective Investigation into Cancer and Nutrition; FFQ, food frequency questionnaire; HDNNCDS, The Harbin Cohort Study on Diet, Nutrition and Chronic Non-Communicable Diseases; HOMA, homeostatic model assessment; HPHS, Harbin People Health Study; HPLC, high-performance liquid chromatography; IRAS, Insulin Resistance and Atherosclerosis Study; IVGTT, intravenous glucose tolerance test; MESA, Multi-Ethnic Study of Atherosclerosis; OGTT, oral glucose tolerance test; RCT, randomized controlled trial; RR, relative risk; SELECT, Selenium and Vitamin E Cancer Prevention Trial; SD, standard deviation; T2D, type 2 diabetes; WHI, Women's Health Initiative; WHO, World Health Organization; WHS, Women's Health Study

Supplementary Table 6. Characteristics of eligible studies on insulin resistance, insulin sensitivity, and beta cell function

Study	Country	Study name	Study design	N	Sex	Age, years	Exposure	Exposure assessment	Outcome	Risk estimate (95% CI)	Adjusted factors
**Insulin resistance**											
Blondin 2013[28]	USA	The BioCycle Study	Cohort	243	Women	18-44	Serum beta-carotene: per unit increase	HPLC	log(HOMA-IR)	β-coefficient -0.02 (-0.06, 0.02)	Age, race, smoking, physical activity, BMI, triglycerides, and total average energy intake
Hozawa 2006[7]	USA	CARDIA	Cohort	4681	Men and women	18-30	Serum beta-carotene: per 1 SD increase	HPLC	HOMA-IR	β-coefficient -0.10 (-0.19, -0.01)	Age, sex, race, study center, education, BMI, total energy intake, ethanol intake, physical activity, systolic blood pressure, plasma levels of total cholesterol, high density lipoprotein cholesterol, and triglycerides, use of vitamin supplements (vitamin A, C, or E or beta-carotene), and baseline insulin
Xiao 2019[29]	China	GNHS	Cohort	2687 NAFLD	Men and women	40-75	Serum ln(beta-carotene): per unit increase	HPLC	HOMA-IR	β-coefficient -0.40 (-0.47,-0.32)	Age, sex, family history of diabetes, alcohol consumption, educational level, occupational socioeconomic class, physical activity, smoking status, vitamin supplementation, BMI, and waist circumference
Zhou 2016[27]	China	HDNNCDS	Cohort	4349	Men and women	20-74	Dietary vitamin C: ≥111.3 vs <68.4 mg/day	FFQ	≥75th percentile of the HOMA-IR	RR 0.82 (0.67, 1.01)	Age at study recruitment, sex, BMI, waist circumference, exercise regularly, total energy intake, hypertension, coronary heart disease, hyperlipemia, body fat percentage, education, current smoking, and family history of type 2 diabetes
		HPHS		1621			Dietary vitamin C: ≥115.6 vs <70.5 mg/day			RR 0.88 (0.69, 1.02)	
		HDNNCDS		4349			Dietary vitamin E: ≥13.53 vs <9.72 mg/day			RR 0.79 (0.57, 1.08)	
		HPHS		1621			Dietary vitamin E: ≥12.96 vs <9.27 mg/day			RR 0.71 (0.38, 1.35)	
He 2021[30]	China		RCT	84 NAFLD	Men and women	18-60	Vitamin C supplementation: 2000 mg/day vs 250 mg/day for 12 weeks		HOMA-IR	Between group difference of mean change -0.43 (-2.20, 1.34)	
							Vitamin C supplementation: 1000 mg/day vs			Between group difference of mean change -0.78 (-2.60, 1.04)	

						250 mg/day for 12 weeks		
Akcam 2011[31]	Turkey	RCT	45	Men and women NAFLD	9-17	Vitamin E supplementation: 400 U/day vs diet and exercise for 6 months	HOMA-IR	Between group difference of mean change -0.60 (-1.65, 0.45)
Ekhlasi 2016[32]	Iran	RCT	30	Men and women NAFLD	25-64	Alpha-tocopherol supplementation: 400 IU/day vs placebo for 2 months	HOMA-IR	Between group difference of mean change -0.01 (-0.15, 0.13)
Homaei 2022[33]	Iran	RCT	100	Men and women NAFLD	10-14	Vitamin E supplementation: 800 U/day vs placebo for 3 months	HOMA-IR	Between group difference of mean change -1.05 (-1.45, -0.65)
Izadi 2019[34]	Iran	RCT	43	Women PCOS	20-40	Vitamin E supplementation: 400 IU/day vs placebo for 2 months	HOMA-IR	Between group difference of mean change -0.29 (-0.86, 0.28)
Manning 2013[35]	New Zealand	RCT	76	Men and women Metabolic syndrome	27-80	Vitamin E supplementation: 100 IU/day vs placebo for 3 months	HOMA-IR	Between group difference of mean change 0.10 (-0.46, 0.66)
						Vitamin E supplementation: 100 IU/day vs placebo for 6 months		Between group difference of mean change 0.20 (-0.44, 0.84)
Pervez 2020[36]	Pakistan	RCT	71	Men and women NAFLD	20-70	Delta-tocotrienol supplementation: 600 mg/day vs placebo for 6 months	HOMA-IR	Between group difference of mean change -0.37 (-0.53, -0.21)
Suleman 2022[37]	Pakistan	RCT	90	Men and women Prediabetes	18-60	Delta-tocotrienol supplementation: 300 mg/day vs placebo for 3 months	HOMA-IR	Between group difference of mean change -0.42 (-0.66, -0.18)
**Insulin sensitivity**								

Ärnlöv 2009[1]	Sweden	ULSAM	Cohort	601	Men	50	Serum beta-carotene: per 1 SD increase	HPLC	HOMA-S	β-coefficient 0.08 (0.06, 0.11)	
							Serum alpha-tocopherol: per 1 SD increase			β-coefficient 0.04 (0.01, 0.06)	
Costacou 2008[38]	USA	IRAS	Cohort	457	Men and women	40-69	Plasma log(alpha-tocopherol): per unit increase	HPLC	log(S_I + 1)	β-coefficient 0.27 (0.09, 0.45)	Cholesterol, log triglycerides, age, gender, ethnicity, clinical centre and family history of diabetes, BMI, energy expended in physical activity, total energy intake, vitamin E intake, vitamin C and beta-carotene (diet and supplements), fat, fibre, calcium and magnesium
							Dietary log(vitamin E): per unit increase	FFQ		β-coefficient 0.01 (-0.13, 0.15)	Total energy intake, age, gender, ethnicity, clinical centre, family history of diabetes, BMI, energy expended in physical activity, lipid levels, vitamin C and beta-carotene (diet and supplements), dietary fat intake, fibre, calcium and magnesium
**Beta cell function**											
Costacou 2008[38]	USA	IRAS	Cohort	457	Men and women	40-69	Plasma log(alpha-tocopherol): per unit increase	HPLC	log(AIR + 397)	β-coefficient 0.14 (-0.08, 0.36)	Cholesterol, log triglycerides, age, gender, ethnicity, clinical centre and family history of diabetes, BMI, energy expended in physical activity, total energy intake, vitamin E intake, vitamin C and beta-carotene (diet and supplements), fat, fibre, calcium and magnesium
							Dietary log(vitamin E): per unit increase	FFQ		β-coefficient 0.07 (-0.09, 0.23)	Total energy intake, age, gender, ethnicity, clinical centre, family history of diabetes, BMI, energy expended in physical activity, lipid levels, vitamin C and beta-carotene (diet and supplements), dietary fat intake, fibre, calcium and magnesium
Zhou 2016[27]	China	HDNNCDS	Cohort	4349	Men and women	20-74	Dietary vitamin C: per 10mg/day increase	FFQ	HOMA-B	β-coefficient 0.43 (0.00, 0.86)	Age at study recruitment, sex, BMI, waist circumference, exercise regularly, total energy intake, hypertension, coronary heart disease, hyperlipemia, body fat percentage, education, current

smoking, and family history of type 2 diabetes

AIR, acute insulin response to glucose; BMI, body mass index; CARDIA, Coronary Artery Risk Development in Young Adults; CI, confidence interval; FFQ, food frequency questionnaire; GNHS, Guangzhou Nutrition and Health Study; HDNNCDS, The Harbin Cohort Study on Diet, Nutrition and Chronic Non-Communicable Diseases; HOMA-B, homeostatic model assessment of beta cell function; HOMA-IR, homeostatic model assessment of insulin resistance; HOMA-S, homeostatic model assessment of insulin sensitivity; HPHS, Harbin People Health Study; HPLC, high-performance liquid chromatography; IRAS, Insulin Resistance and Atherosclerosis Study; NAFLD, non-alcoholic fatty liver disease; PCOS, polycystic ovary syndrome; RCT, randomized controlled trial; RR, relative risk; S_I, insulin sensitivity index; ULSAM, Uppsala Longitudinal Study of Adult Men; SD, standard deviation

Supplementary Table 7. Certainty of evidence assessment using the GRADE system

**Certainty assessment**							**N**	**Cases**	**Comparison**	**Risk estimate (95% CI)**	**Certainty**	**Outcome**
**Studies**	**Study design**	**Risk of bias**	**Inconsistency**	**Indirectness**	**Imprecision**	**Other considerations**						
**Dietary vitamin C**												
6	Cohort	serious	serious	not serious	not serious	Non-linear dose-response relationship	64654	2636	High vs low	**RR 0.81** (0.66 to 0.99)	⊕⊕⊕◯ MODERATE	T2D
6	Cohort	serious	not serious	not serious	not serious	None	64654	2636	Per 10 mg/day	**RR 0.99** (0.97 to 1.00)	⊕⊕⊕◯ MODERATE	T2D
2	Cohort	serious	not serious	serious	not serious	None	5970	788	High vs low	**RR 0.85** (0.74 to 0.98)	⊕⊕◯◯ LOW	Insulin resistance
**Dietary vitamin E**												
8	Cohort	serious	not serious	not serious	not serious	Non-linear dose-response relationship	66233	2841	High vs low	**RR 0.81** (0.71 to 0.92)	⊕⊕⊕⊕ HIGH	T2D
8	Cohort	serious	serious	not serious	not serious	None	66233	2841	Per 1 mg/day	**RR 0.98** (0.96 to 1.00)	⊕⊕◯◯ LOW	T2D

Certainty assessment							N	Cases	Comparison	Risk estimate (95% CI)	Certainty	Outcome
Studies	Study design	Risk of bias	Inconsistency	Indirectness	Imprecision	Other considerations						
2	Cohort	serious	not serious	serious	serious	None	5970	797	High vs low	**RR 0.77** (0.58 to 1.03)	⊕◯◯◯ VERY LOW	Insulin resistance
**Circulating vitamin E (alpha-tocopherol)**												
5	4 Cohorts 1 Nested case-control	serious	serious	not serious	serious	No dose-response relationship	9859	732	High vs low	**RR 0.58** (0.27 to 1.24)	⊕◯◯◯ VERY LOW	T2D
5	4 Cohorts 1 Nested case-control	serious	serious	not serious	serious	None	9859	732	Per SD increment	**RR 0.90** (0.73 to 1.11)	⊕◯◯◯ VERY LOW	T2D
**Vitamin E supplementation**												
4	RCT	not serious	not serious	not serious	serious	None	76452	4318	Active vs placebo	**RR 1.01** (0.93 to 1.10)	⊕⊕⊕◯ MODERATE	T2D
7	RCT	not serious	very serious	serious	not serious	None	455	NA	Active vs placebo	**Between group difference of mean change -0.35** (-0.65, -0.06)	⊕◯◯◯ VERY LOW	HOMA-IR
**Dietary beta-carotene**												

Certainty assessment							N	Cases	Comparison	Risk estimate (95% CI)	Certainty	Outcome
Studies	Study design	Risk of bias	Inconsistency	Indirectness	Imprecision	Other considerations						
5	Cohort	serious	not serious	not serious	not serious	Non-linear dose-response relationship	72938	2414	High vs low	**RR 0.83** (0.71 to 0.97)	⊕⊕⊕⊕ HIGH	T2D
5	Cohort	serious	not serious	not serious	not serious	None	72938	2414	Per 1 mg/day	**RR 0.95** (0.91 to 1.00)	⊕⊕⊕◯ MODERATE	T2D
**Circulating beta-carotene**												
6	3 Cohort 2 Nested case-control 1 Case-cohort	serious	serious	not serious	not serious	Non-linear dose-response relationship	32657	10818	High vs low	**RR 0.61** (0.43 to 0.87)	⊕⊕⊕◯ MODERATE	T2D
6	3 Cohort 2 Nested case-control 1 Case-cohort	serious	serious	not serious	not serious	None	36474	10911	Per SD increment	**RR 0.82** (0.70 to 0.98)	⊕⊕◯◯ LOW	T2D
**Beta-carotene supplementation**												
3	RCT	not serious	not serious	not serious	serious	None	41741	2061	Active vs placebo	**RR 0.98** (0.90 to 1.07)	⊕⊕⊕◯ MODERATE	T2D

CI, confidence interval; HOMA-IR, homeostatic model assessment of insulin resistance; NA, not applicable; RCT, randomized controlled trial; RR, relative risk; SD, standard deviation; T2D, type 2 diabetes

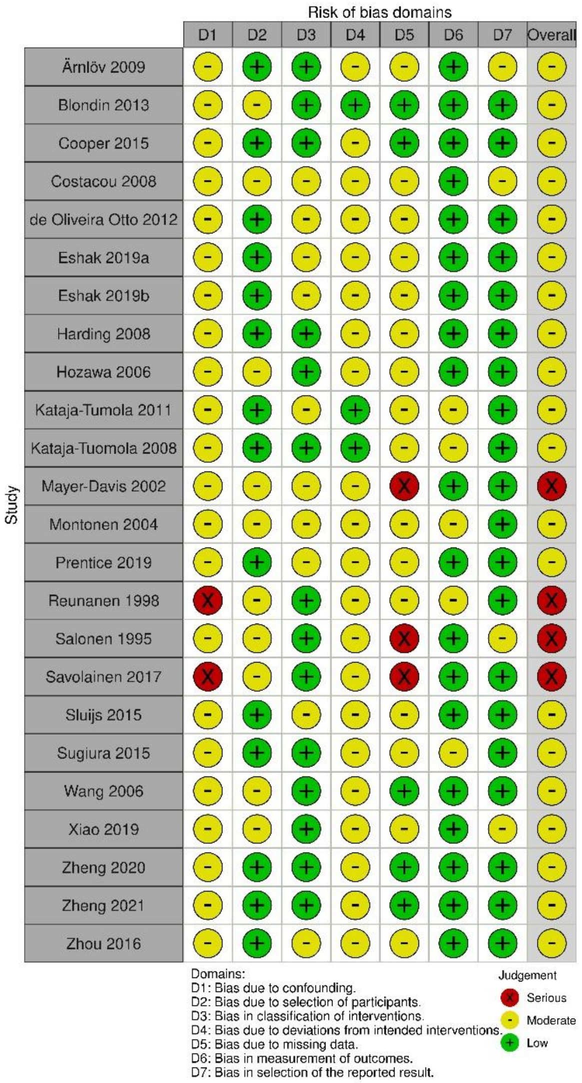

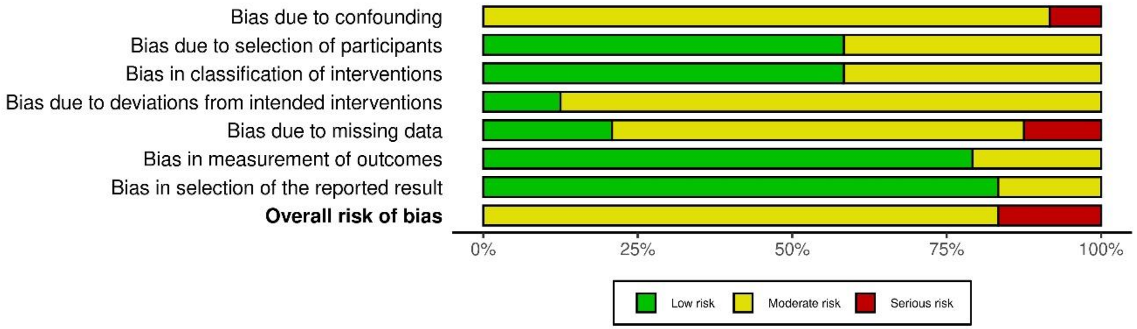

Supplementary Figure 1. Risk of bias assessment of observational studies using ROBINS-I, generated with the robvis tool

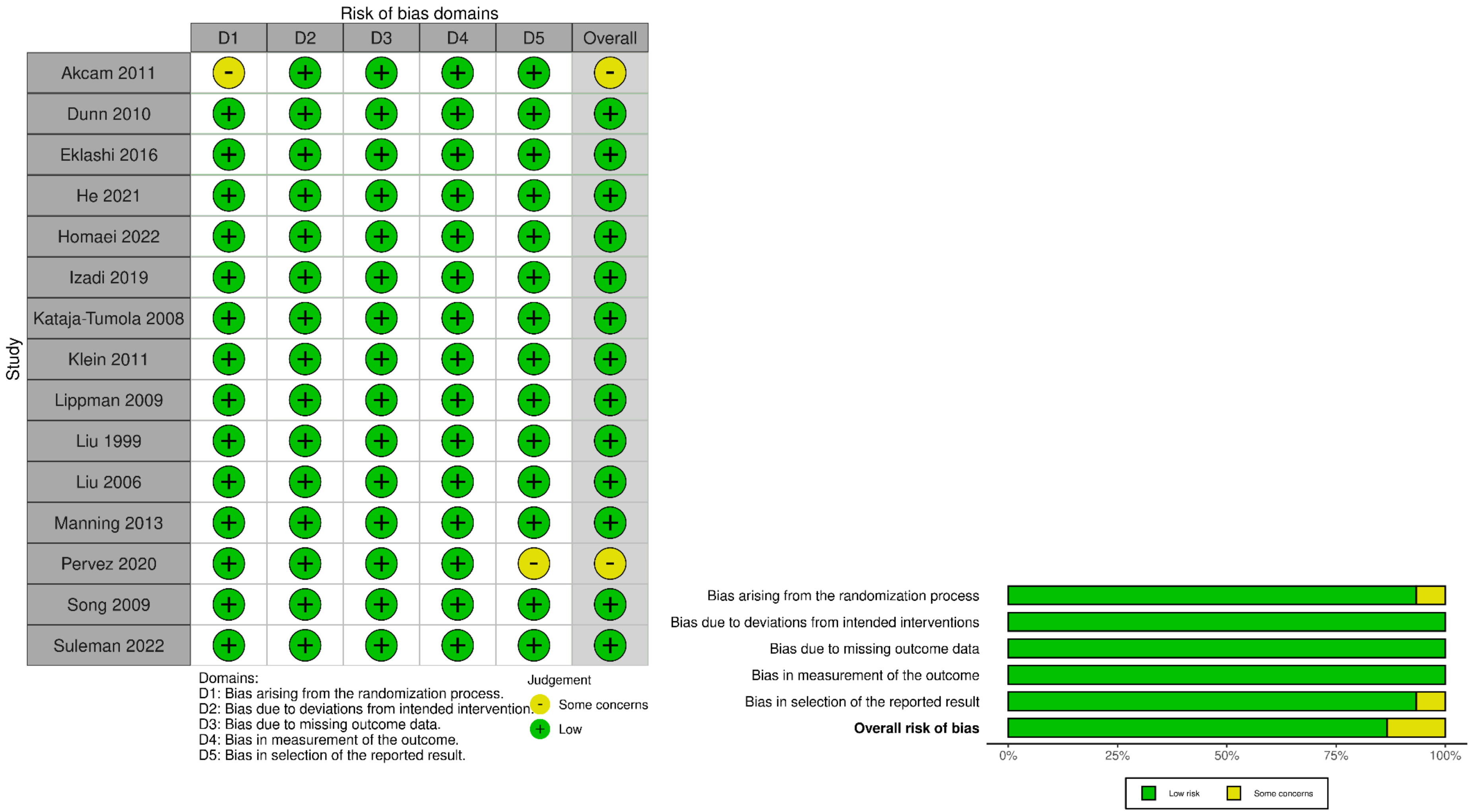

Supplementary Figure 2. Risk of bias assessment of randomized controlled trials using RoB 2.0, generated with the robvis tool

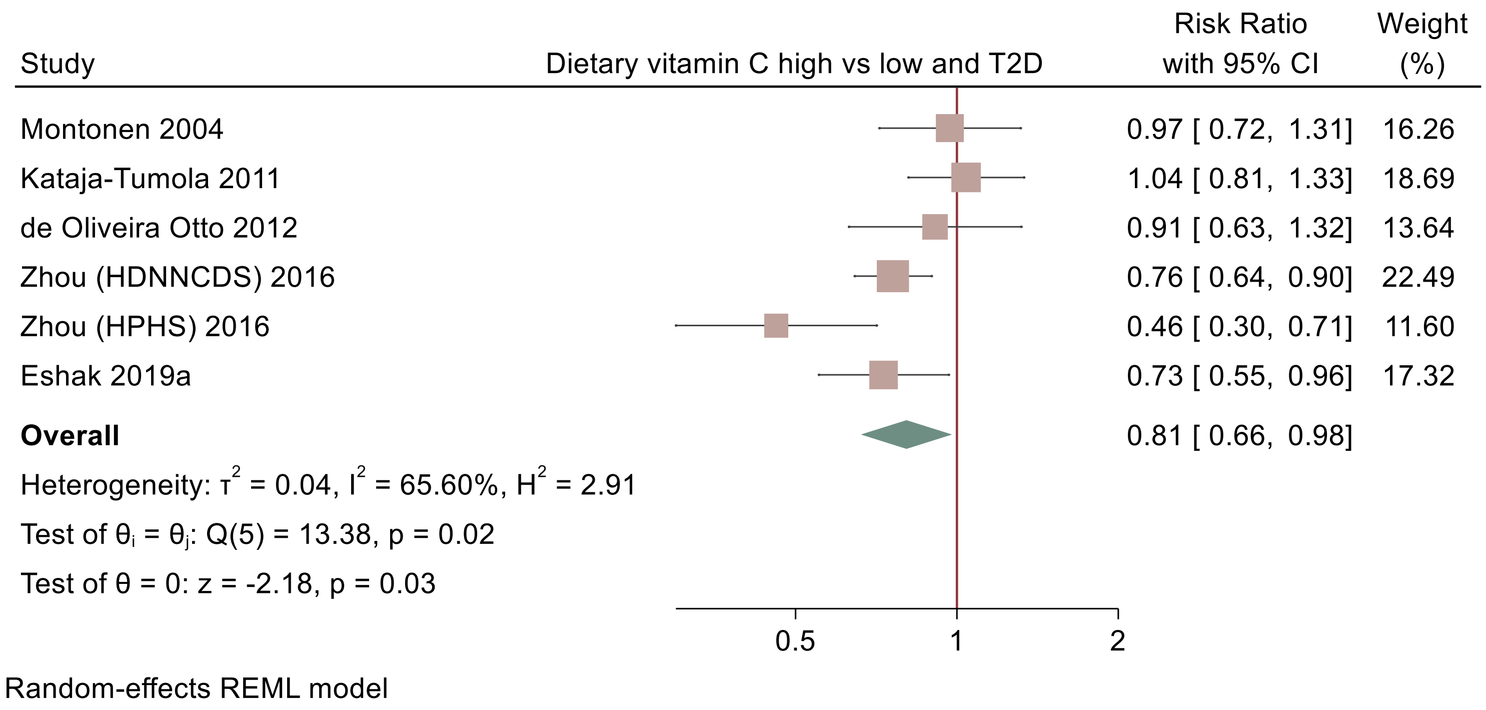

Supplementary Figure 3. Summary of relative risk of type 2 diabetes for high vs low dietary vitamin C

CI, confidence interval; HDNNCDS, The Harbin Cohort Study on Diet; HPHS, Harbin People Health Study; T2D, type 2 diabetes

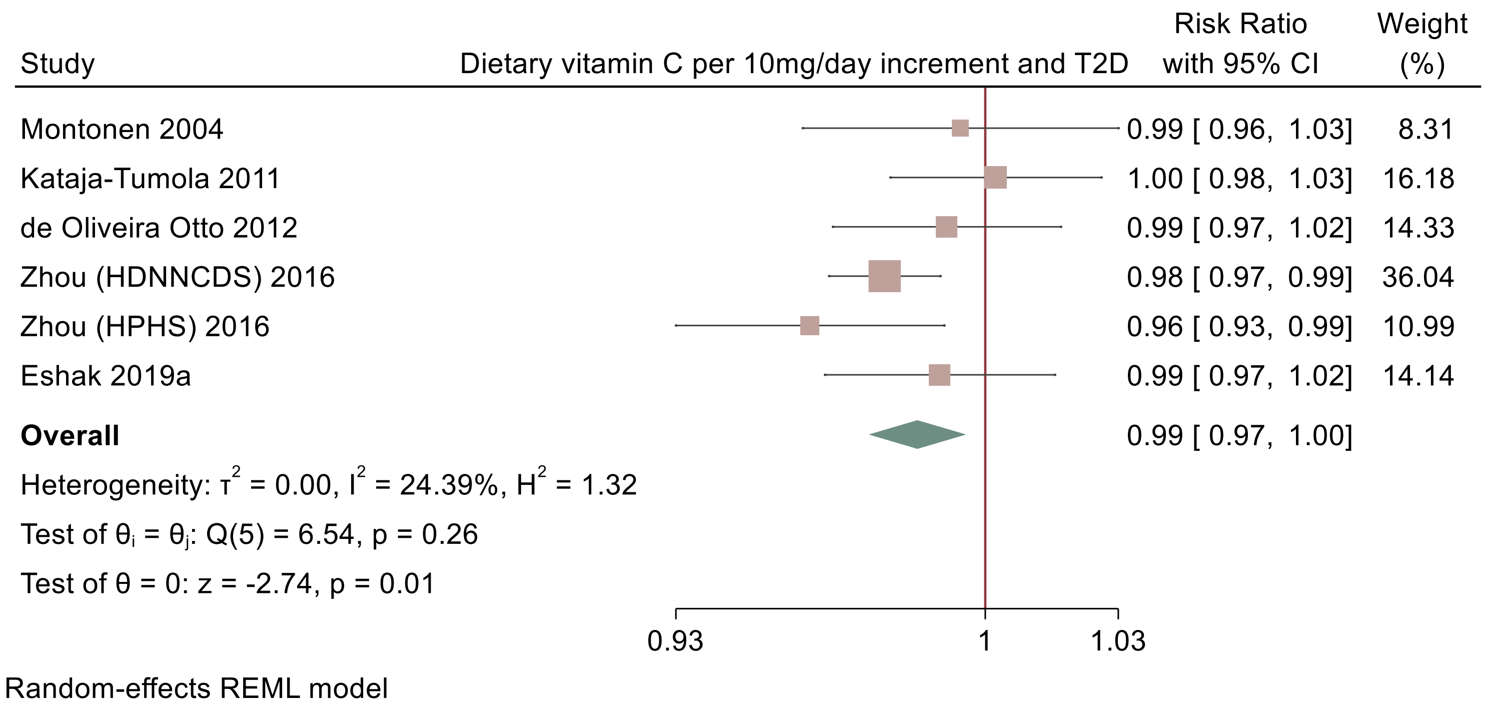

Supplementary Figure 4. Summary of relative risk of type 2 diabetes per 10 mg/day increment in dietary vitamin C

CI, confidence interval; HDNNCDS, The Harbin Cohort Study on Diet; HPHS, Harbin People Health Study; T2D, type 2 diabetes

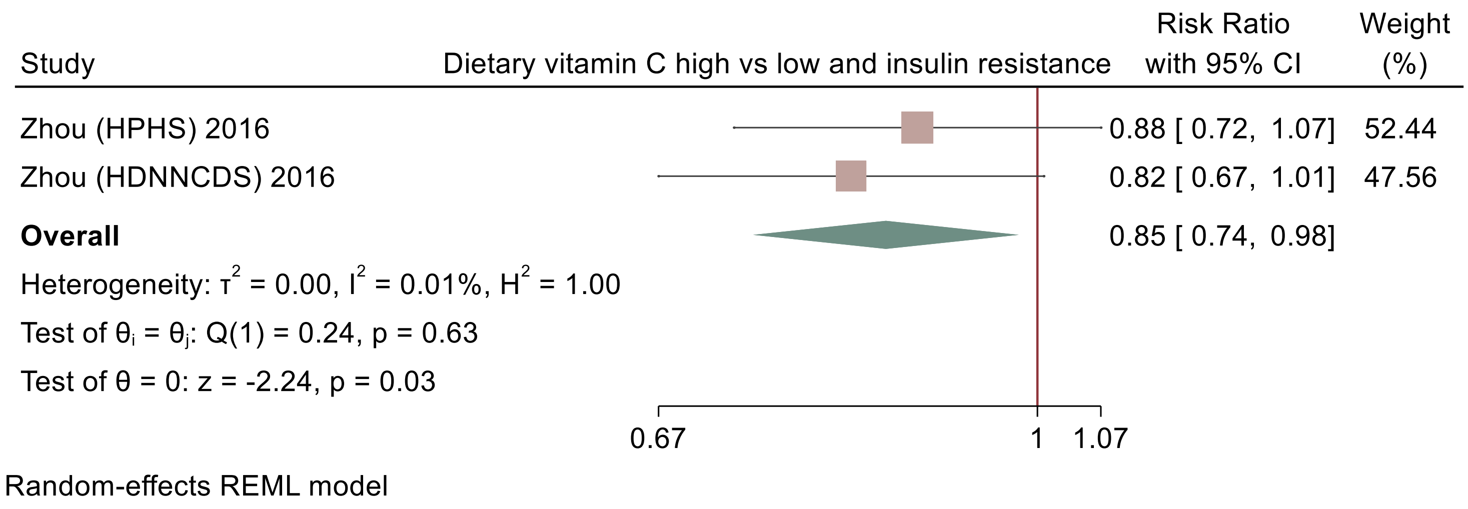

Supplementary Figure 5. Summary of relative risk of insulin resistance for high vs low dietary vitamin C

CI, confidence interval; HDNNCDS, The Harbin Cohort Study on Diet; HPHS, Harbin People Health Study

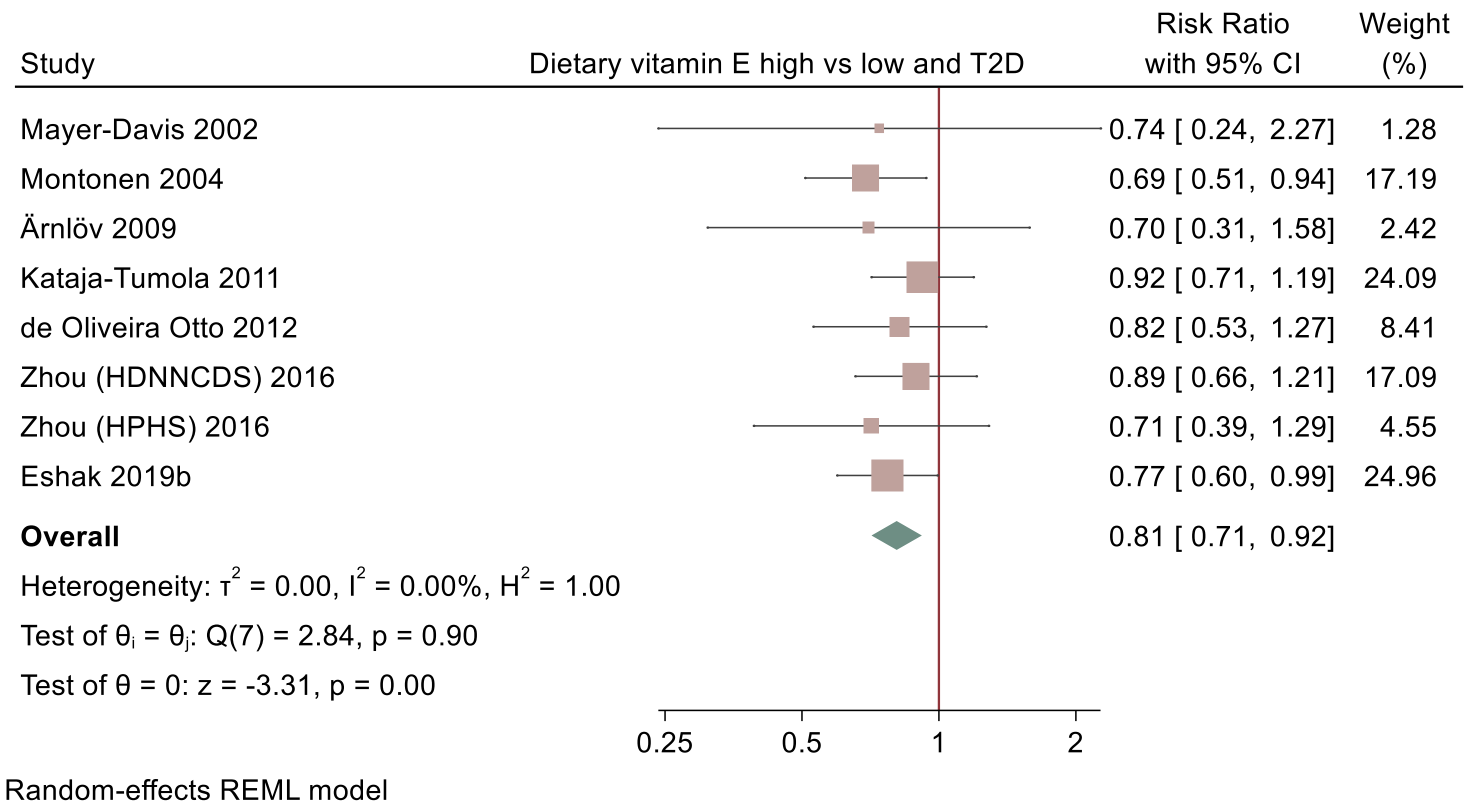

Supplementary Figure 6. Summary of relative risk of type 2 diabetes for high vs low dietary vitamin E

CI, confidence interval; HDNNCDS, The Harbin Cohort Study on Diet; HPHS, Harbin People Health Study; T2D, type 2 diabetes

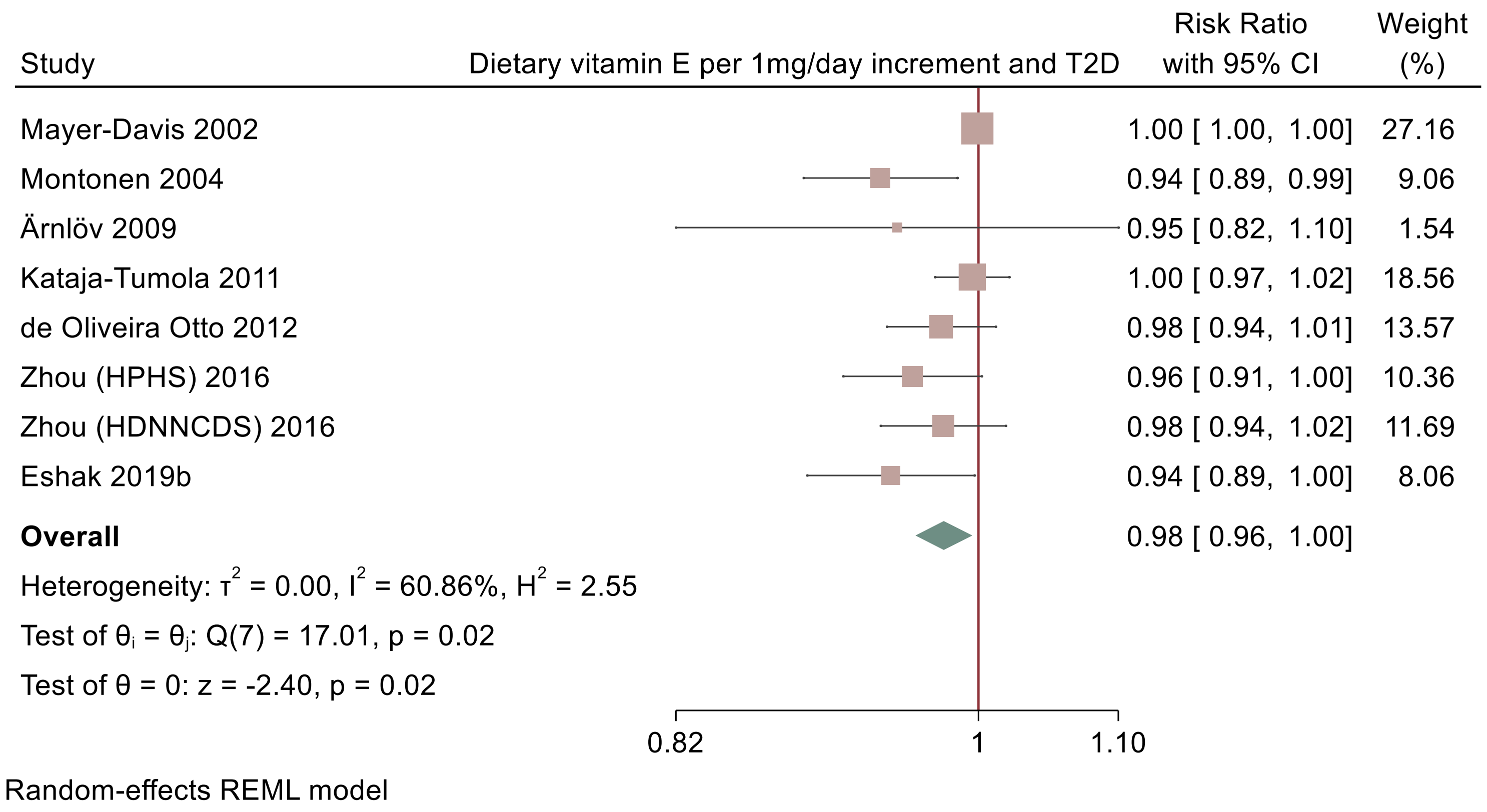

Supplementary Figure 7. Summary of relative risk of type 2 diabetes per 1 mg/day increment in dietary vitamin E

CI, confidence interval; HDNNCDS, The Harbin Cohort Study on Diet; HPHS, Harbin People Health Study; T2D, type 2 diabetes

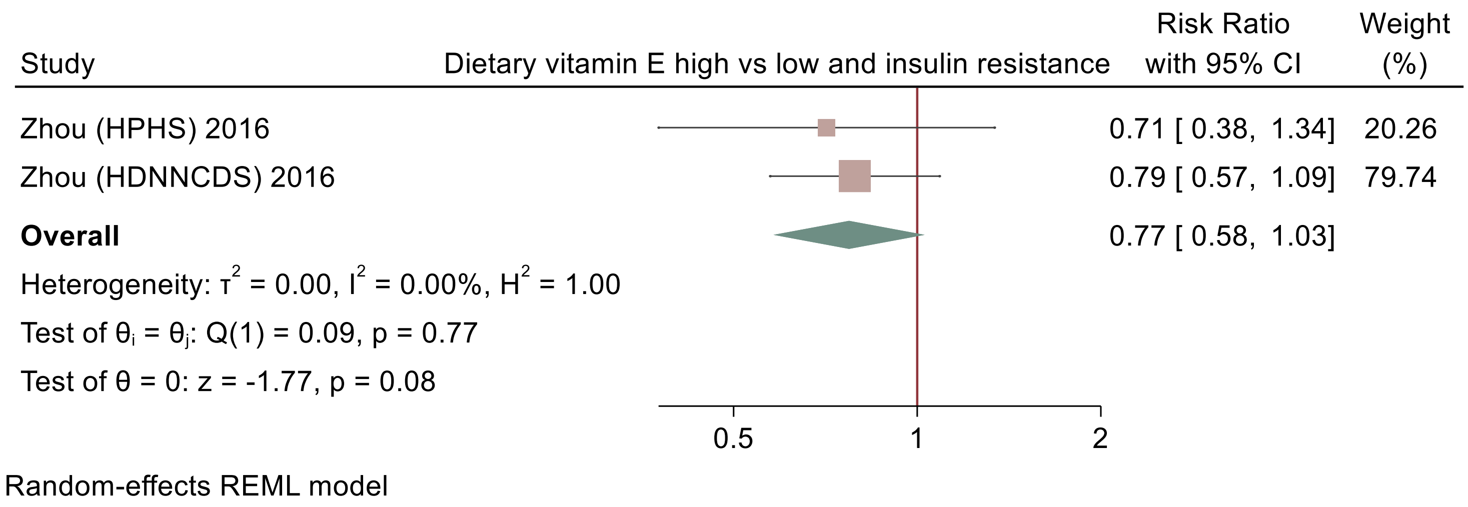

Supplementary Figure 8. Summary of relative risk of insulin resistance for high vs low dietary vitamin E

CI, confidence interval; HDNNCDS, The Harbin Cohort Study on Diet; HPHS, Harbin People Health Study

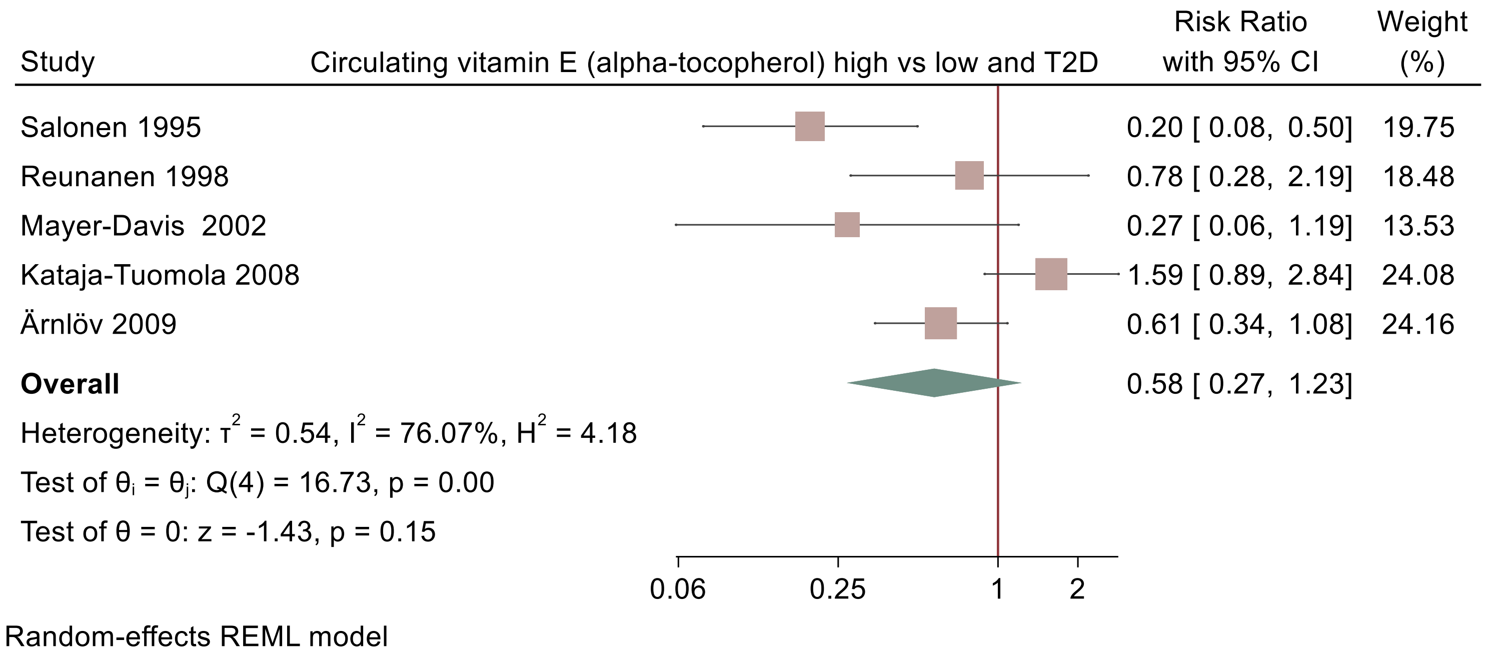

Supplementary Figure 9. Summary of relative risk of type 2 diabetes for high vs low circulating vitamin E (alpha-tocopherol)

CI, confidence interval; T2D, type 2 diabetes

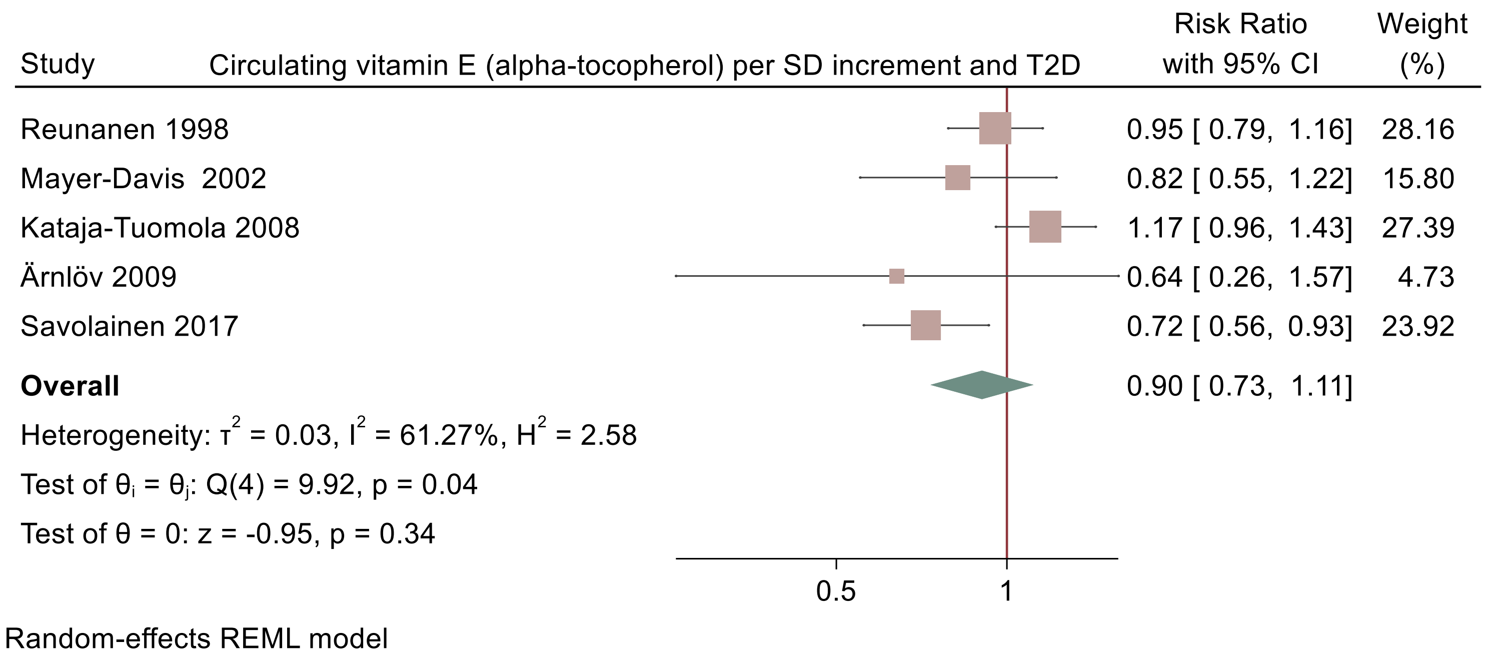

Supplementary Figure 10. Summary of relative risk of type 2 diabetes per 1 standard deviation increment in circulating vitamin E (alpha-tocopherol)

CI, confidence interval; SD, standard deviation; T2D, type 2 diabetes

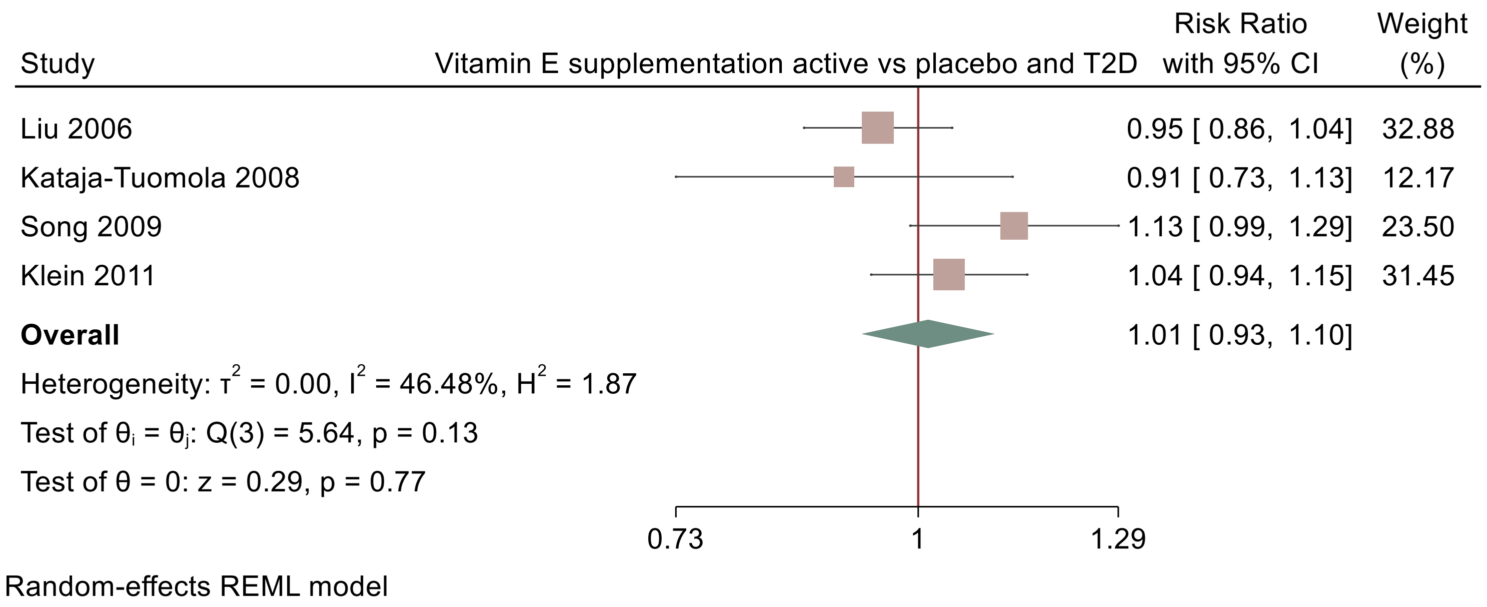

Supplementary Figure 11. Summary of relative risk of type 2 diabetes for vitamin E (alpha-tocopherol) supplementation vs placebo

CI, confidence interval; T2D, type 2 diabetes

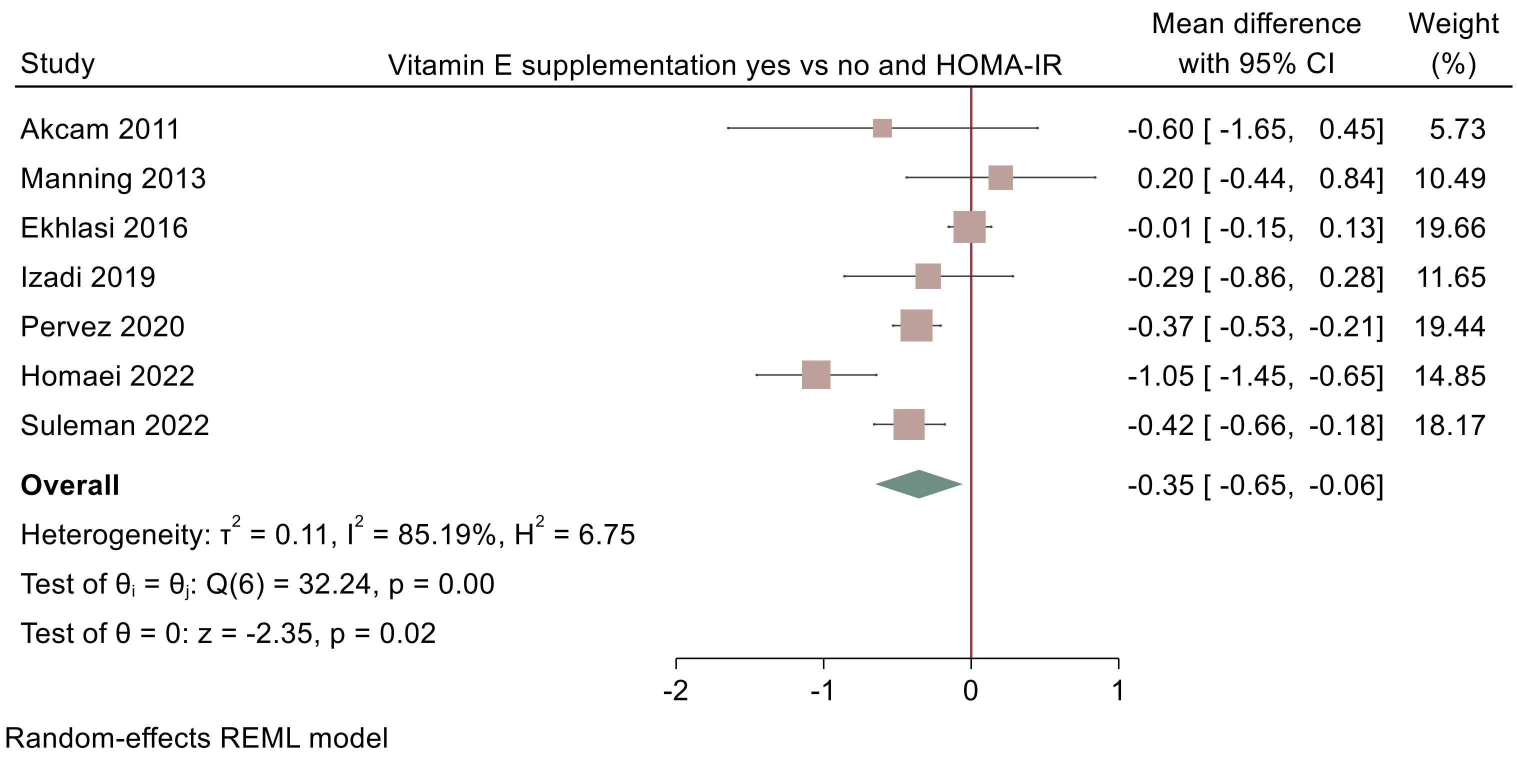

Supplementary Figure 12. Summary of mean difference of the change in HOMA-IR from baseline between individuals using vitamin E supplements or placebo/lifestyle intervention

CI, confidence interval; HOMA-IR, homeostatic model assessment of insulin resistance

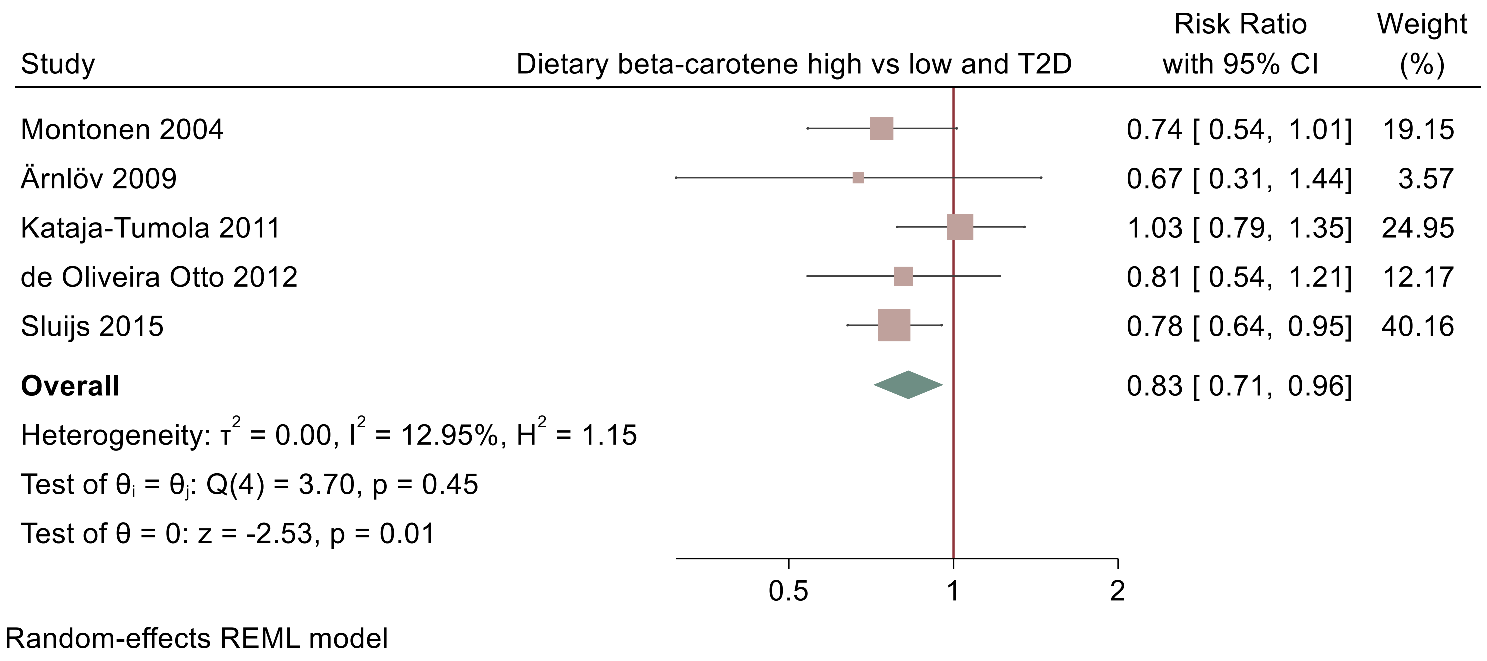

Supplementary Figure 13. Summary of relative risk of type 2 diabetes for high vs low dietary beta-carotene

CI, confidence interval; T2D, type 2 diabetes

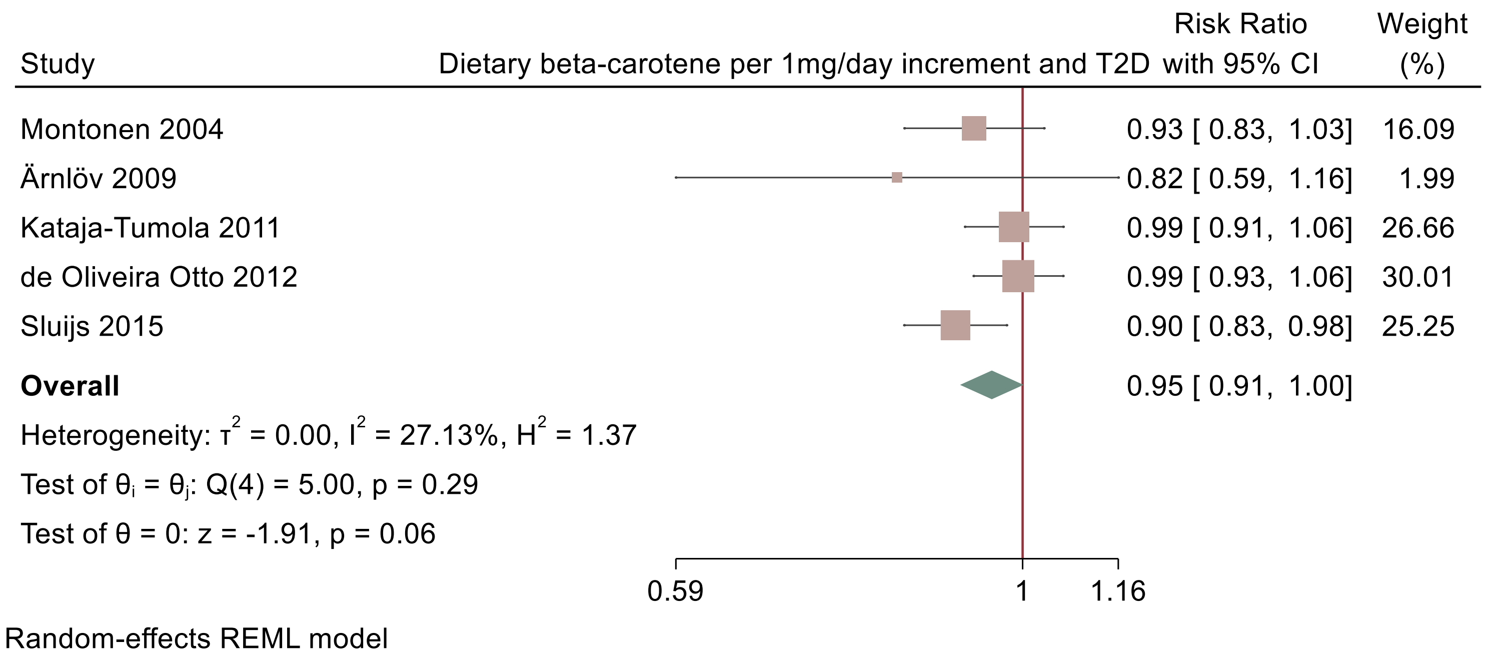

Supplementary Figure 14. Summary of relative risk of type 2 diabetes per 1 mg/day increment in dietary beta-carotene

CI, confidence interval; T2D, type 2 diabetes

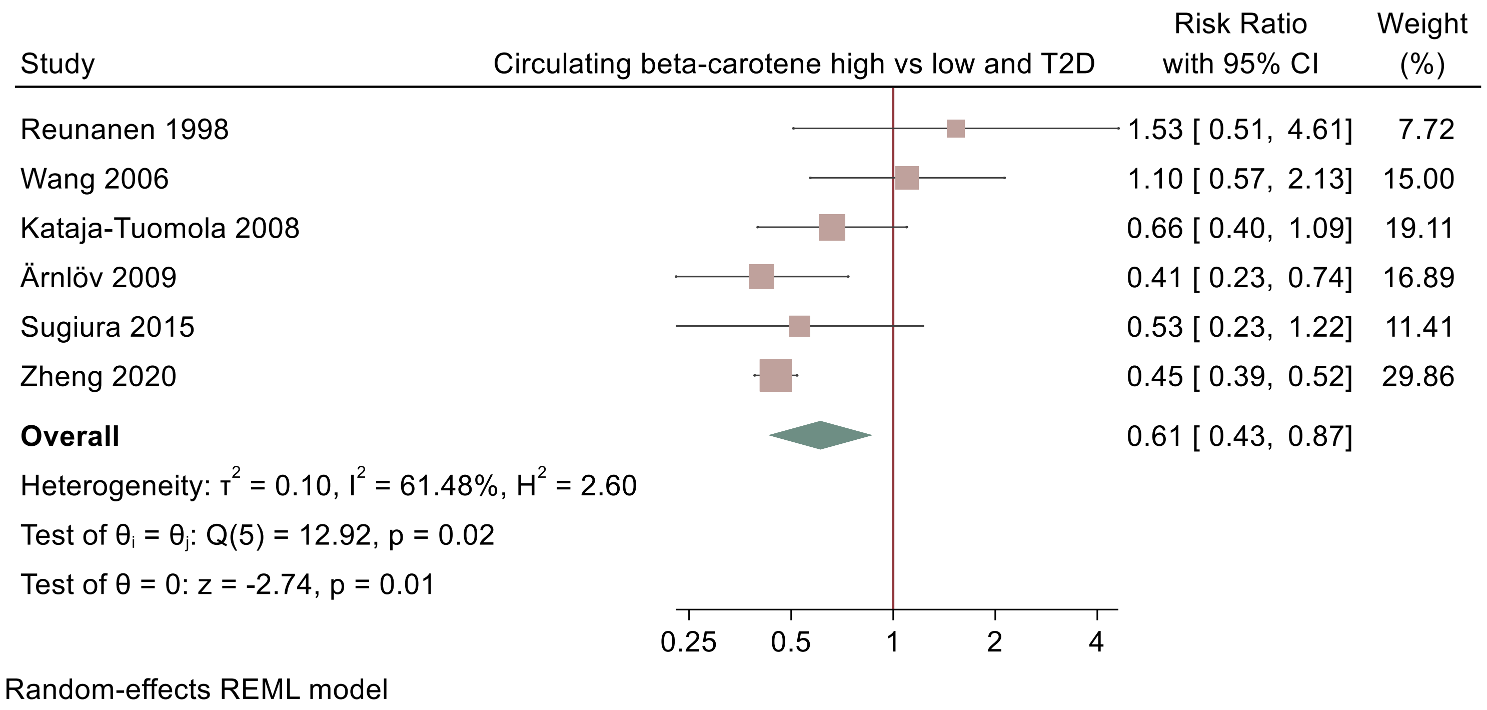

Supplementary Figure 15. Summary of relative risk of type 2 diabetes for high vs low circulating beta-carotene

CI, confidence interval; T2D, type 2 diabetes

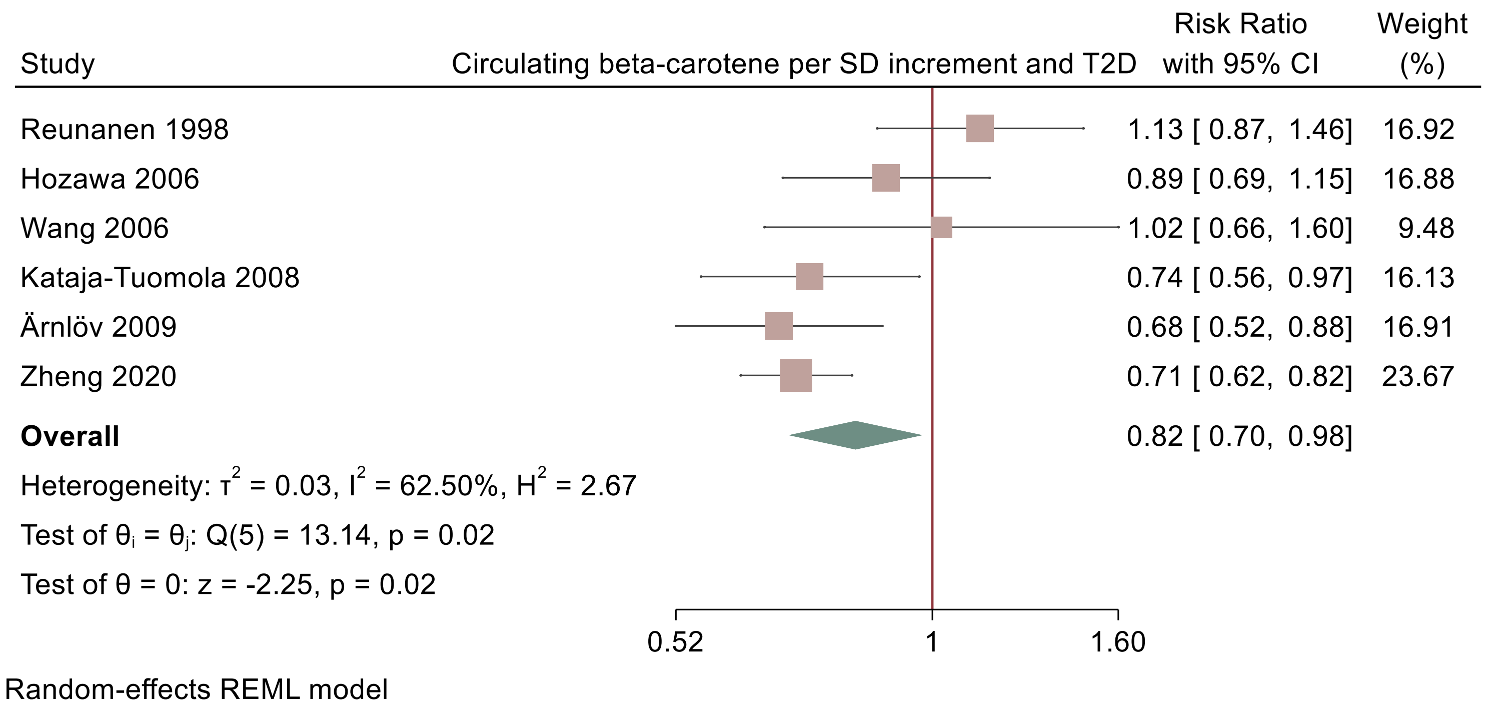

Supplementary Figure 16. Summary of relative risk of type 2 diabetes per 1 standard deviation increment in circulating beta-carotene

CI, confidence interval; SD, standard deviation; T2D, type 2 diabetes

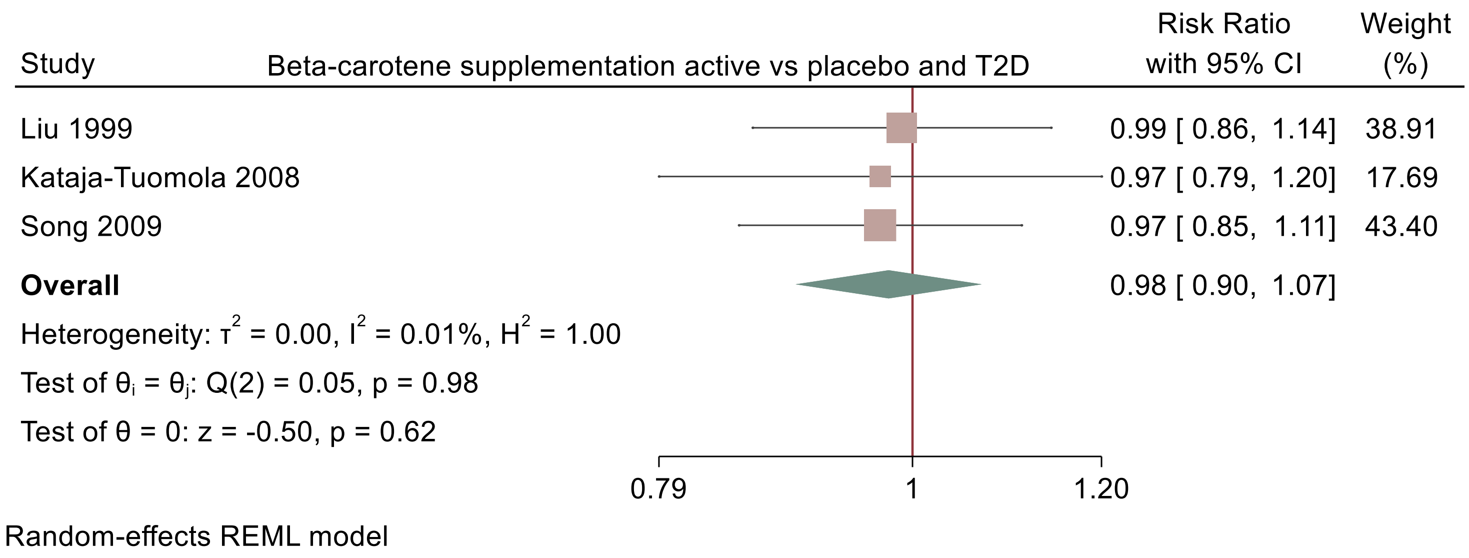

Supplementary Figure 17. Summary of relative risk of type 2 diabetes for beta-carotene supplementation vs placebo

CI, confidence interval; SD, standard deviation; T2D, type 2 diabetes

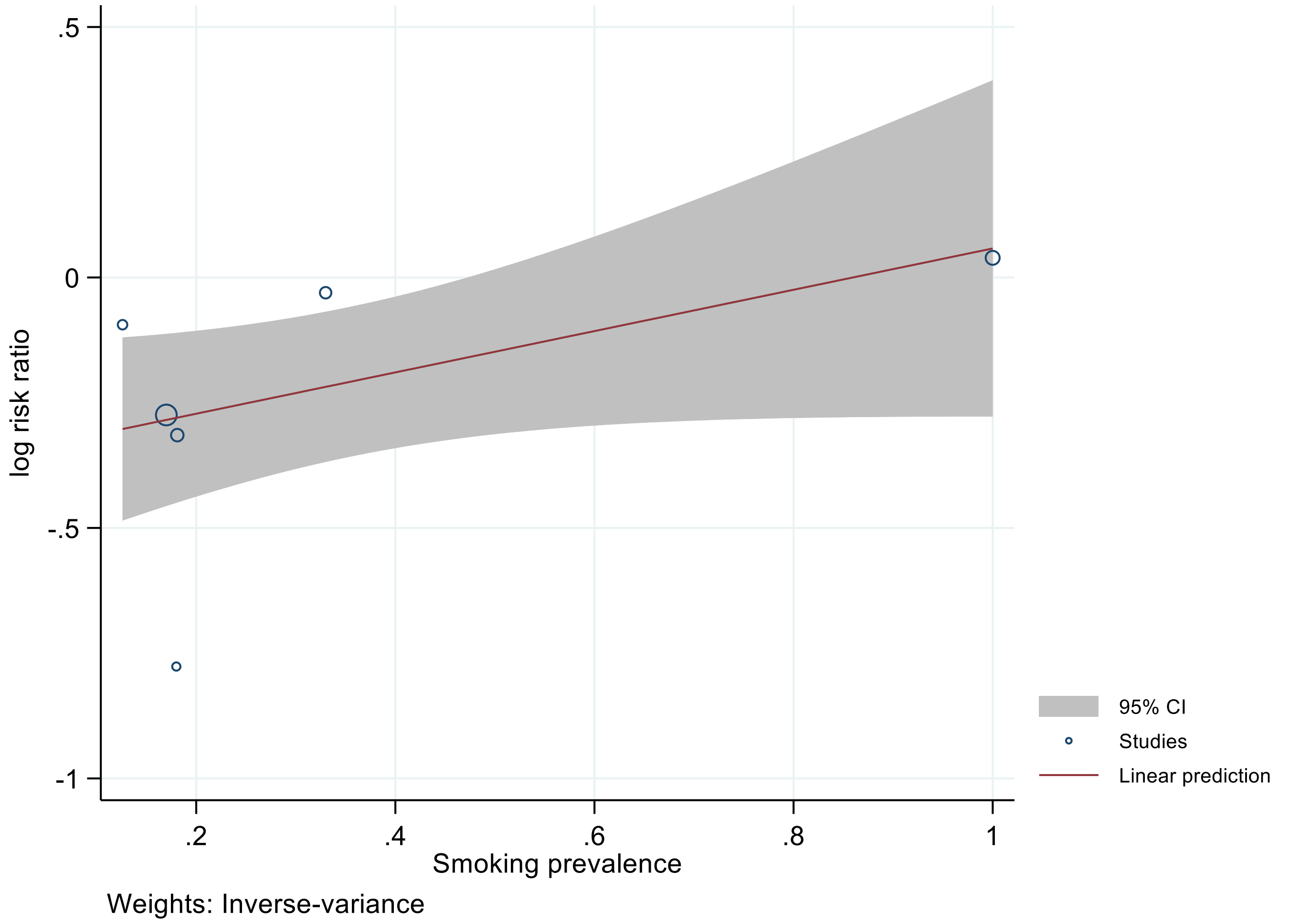

Supplementary Figure 18. Meta-regression for high vs low dietary vitamin C and type 2 diabetes with smoking prevalence within the cohort as effect modifier (*p*=0.081)

CI, confidence interval

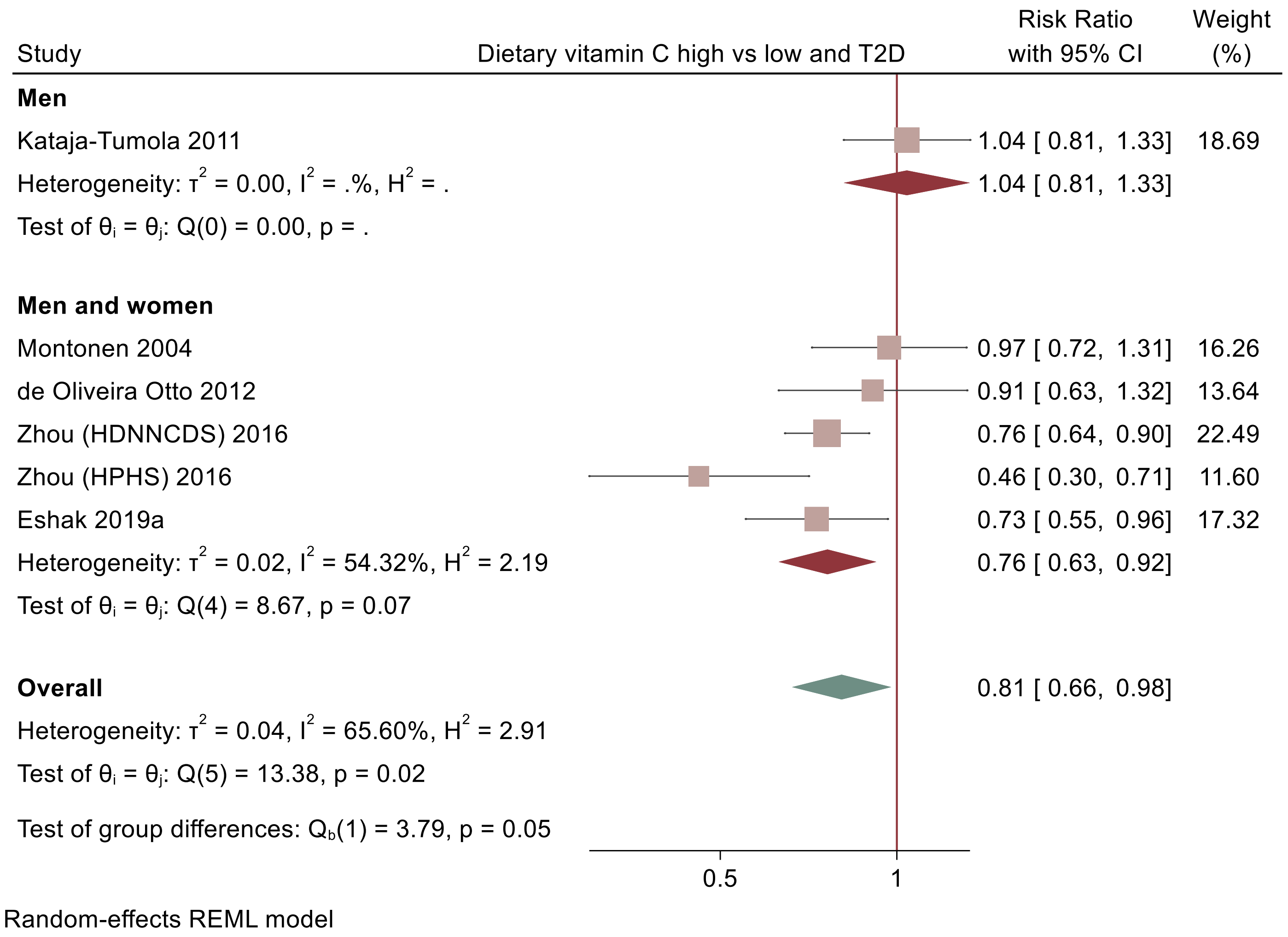

Supplementary Figure 19. Subgroup analysis for high vs low dietary vitamin C and type 2 diabetes based on sex

CI, confidence interval; HDNNCDS, The Harbin Cohort Study on Diet; HPHS, Harbin People Health Study; T2D, type 2 diabetes

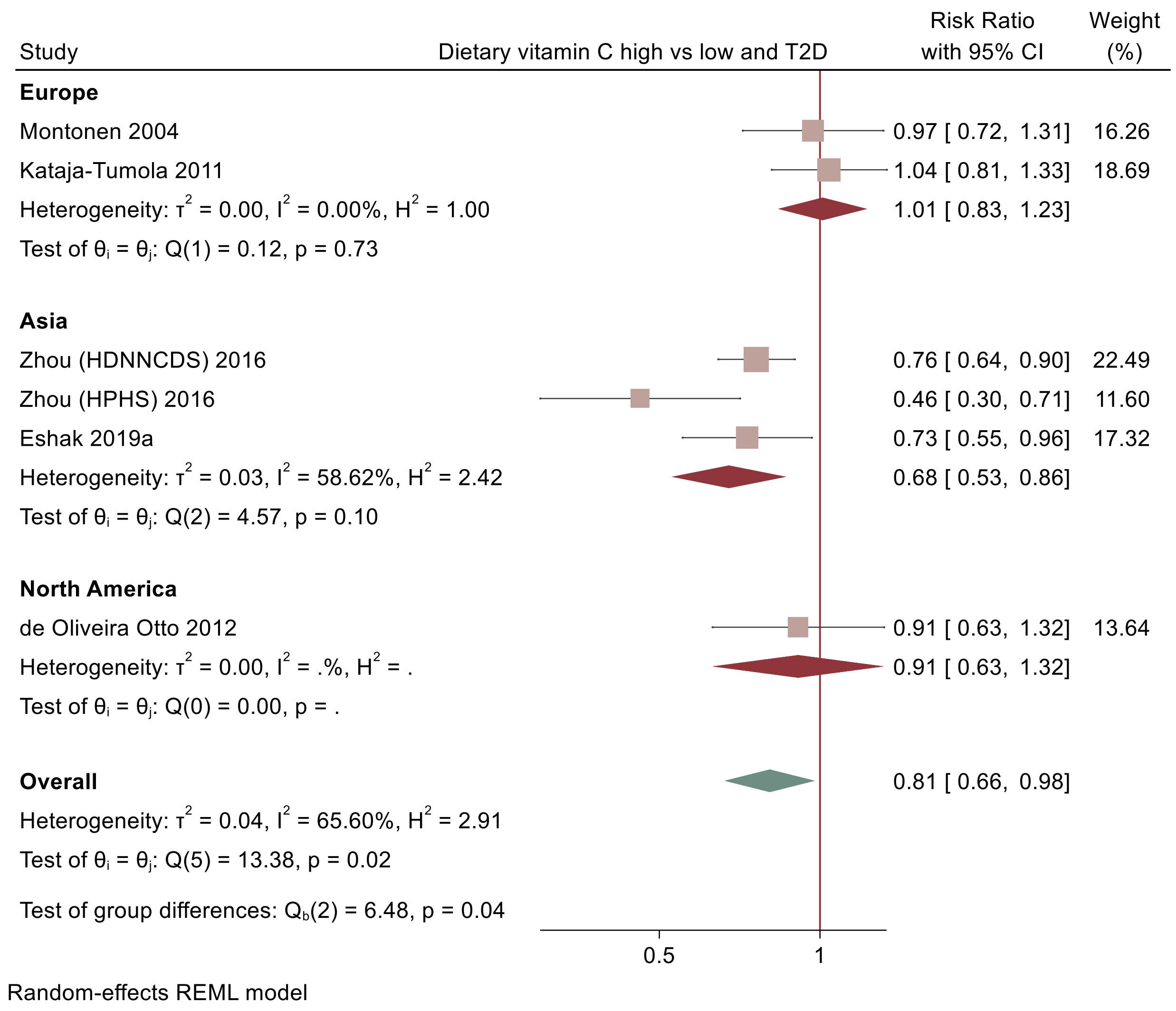

Supplementary Figure 20. Subgroup analysis for high vs low dietary vitamin C and type 2 diabetes based on geographic region

CI, confidence interval; HDNNCDS, The Harbin Cohort Study on Diet; HPHS, Harbin People Health Study; T2D, type 2 diabetes

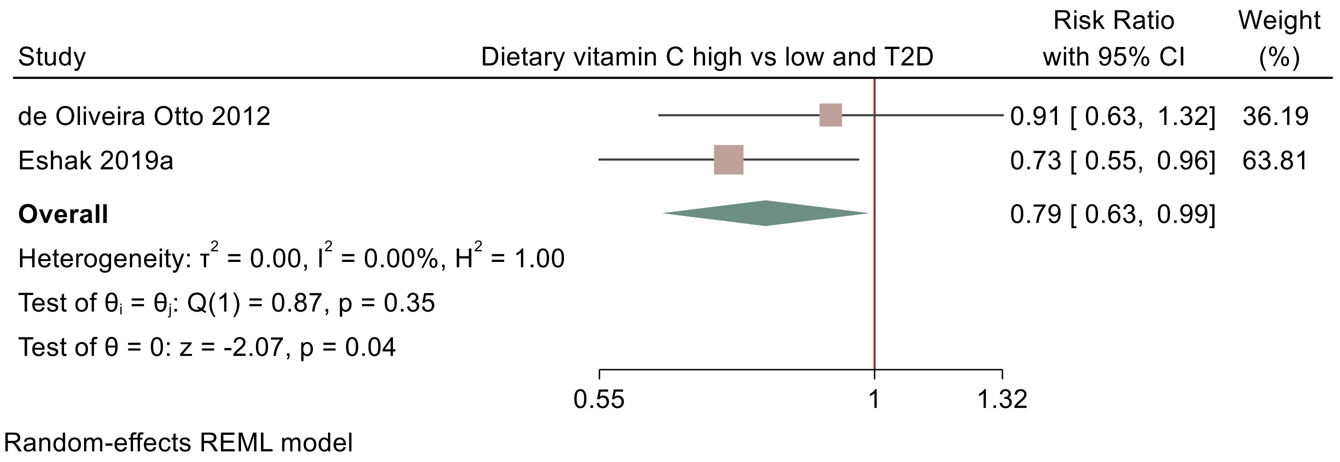

Supplementary Figure 21. Sensitivity analysis for high vs low dietary vitamin C and type 2 diabetes including only studies that had adjusted for dietary co-exposures
CI, confidence interval; T2D, type 2 diabetes

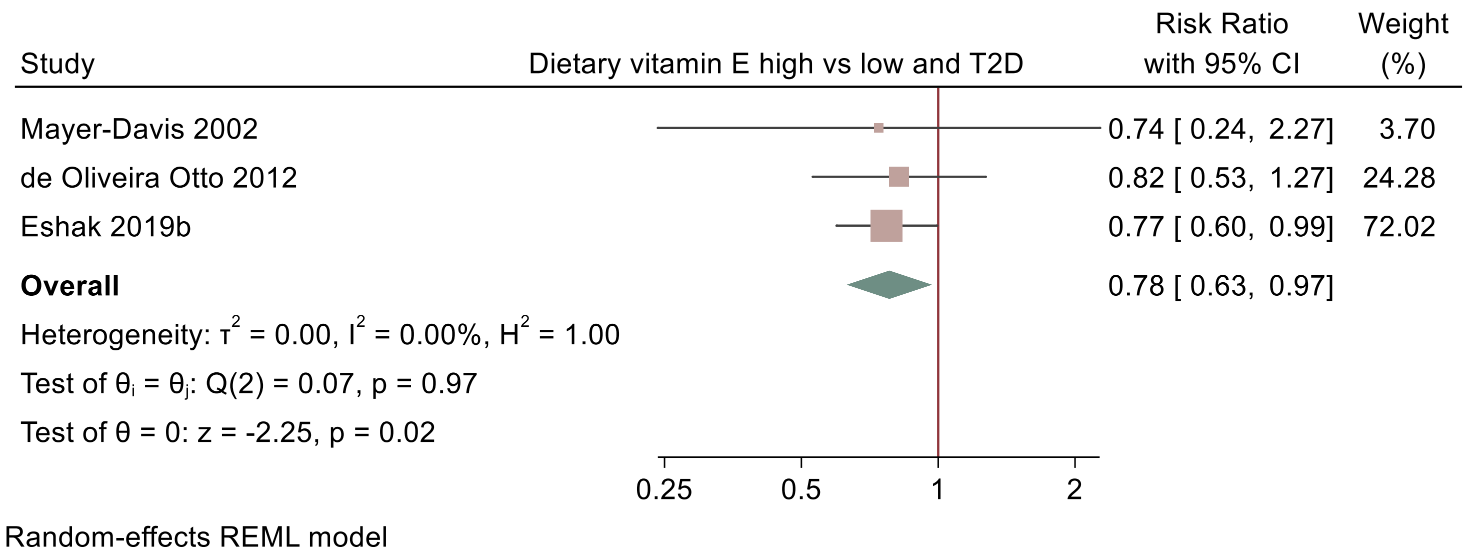

Supplementary Figure 22. Sensitivity analysis for high vs low dietary vitamin E and type 2 diabetes including only studies that had adjusted for dietary co-exposures

CI, confidence interval; T2D, type 2 diabetes

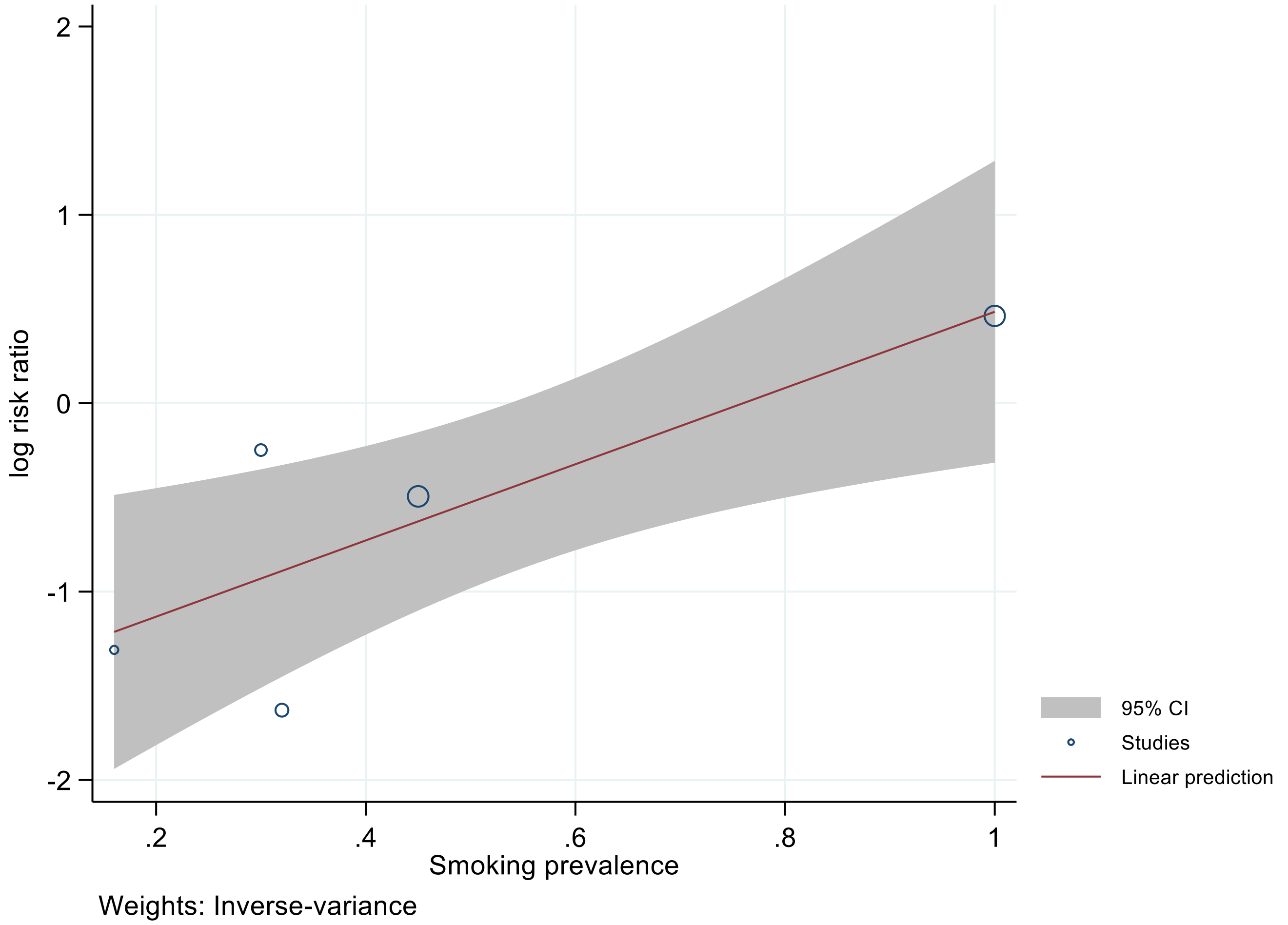

Supplementary Figure 23. Meta-regression for high vs low circulating vitamin E (alpha-tocopherol) and type 2 diabetes with smoking prevalence within the cohort as effect modifier (p=0.007)

CI, confidence interval

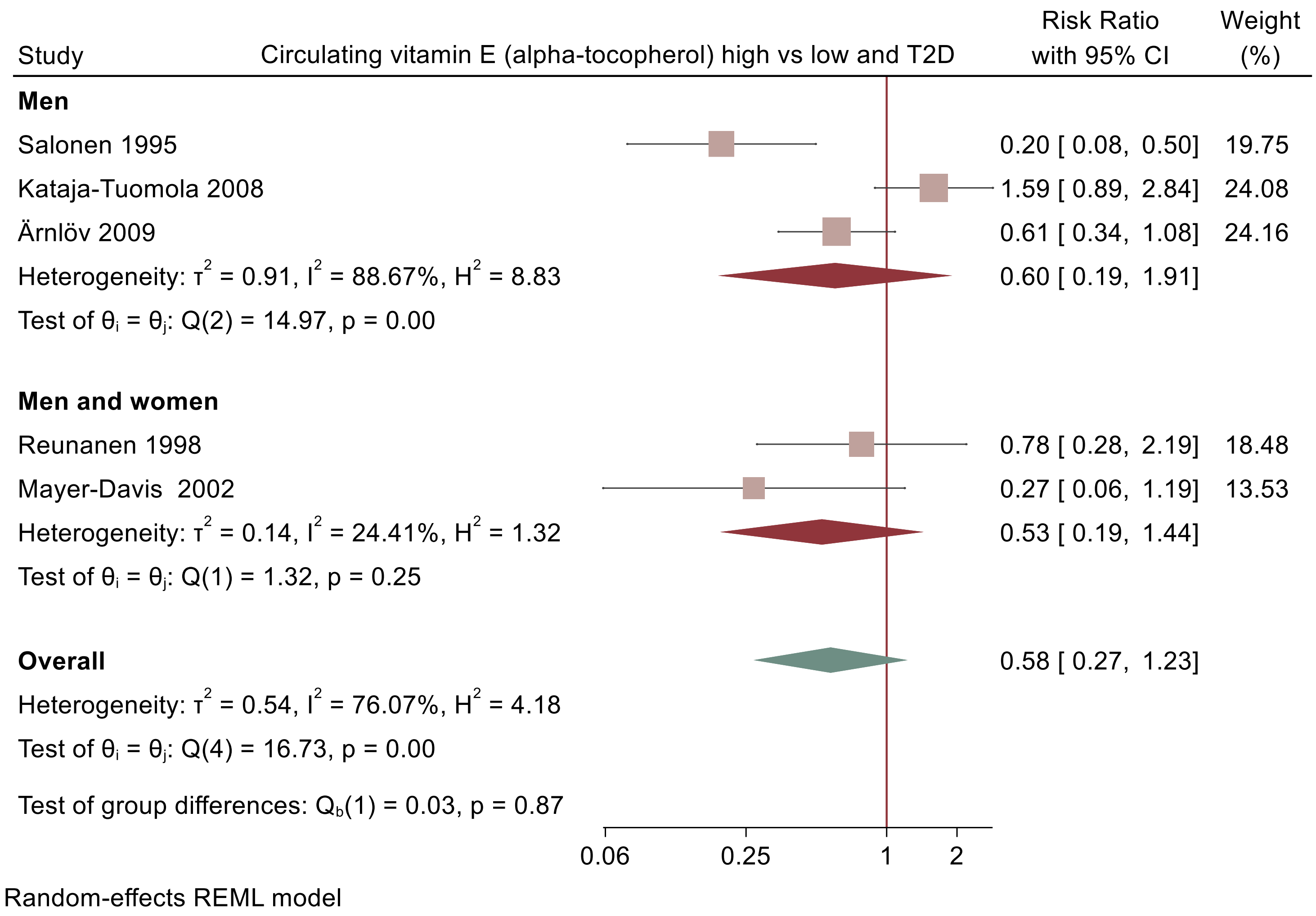

Supplementary Figure 24. Subgroup analysis for high vs low circulating vitamin E (alpha-tocopherol) and type 2 diabetes based on sex

CI, confidence interval; T2D, type 2 diabetes

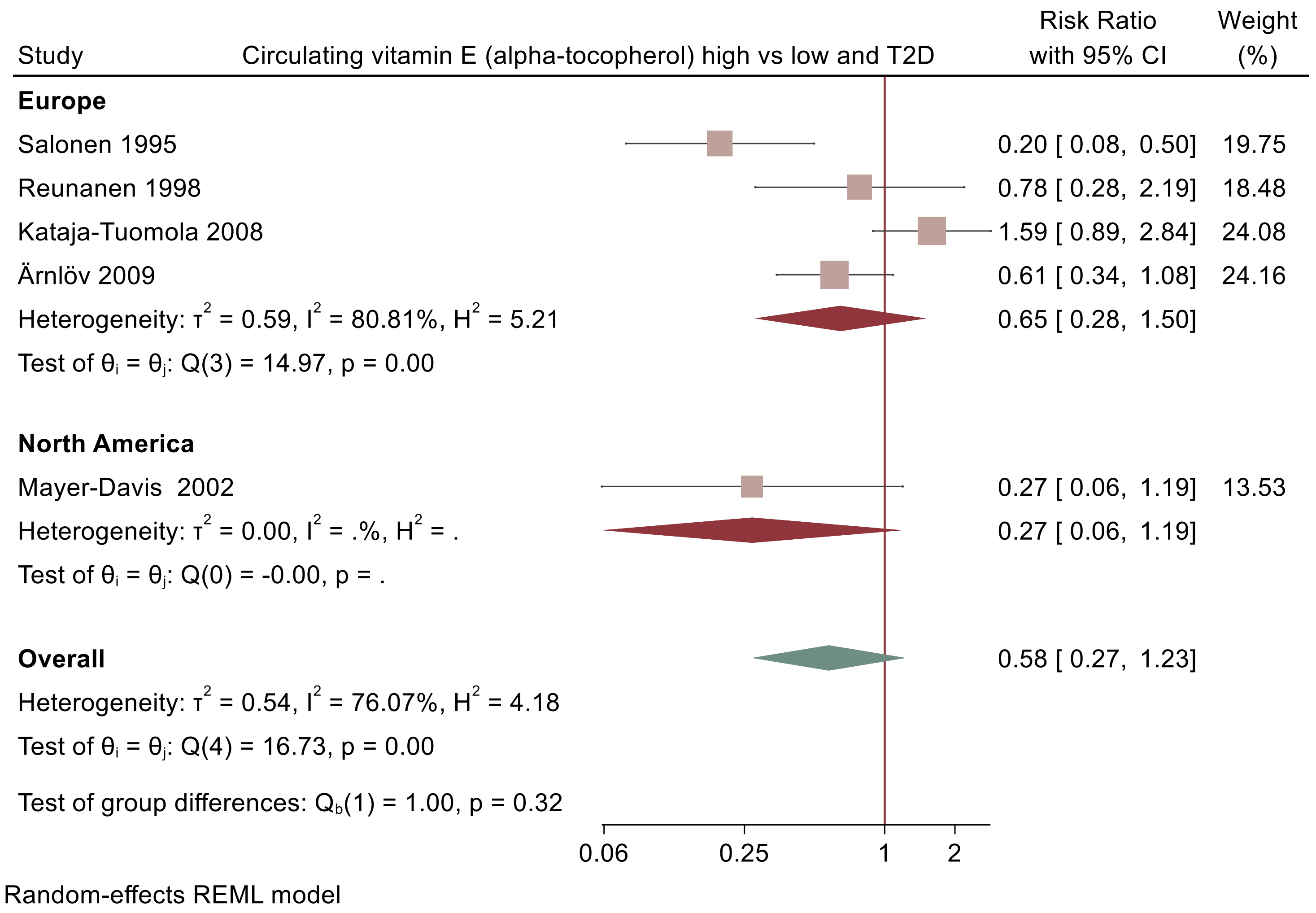

Supplementary Figure 25. Subgroup analysis for high vs low circulating vitamin E (alpha-tocopherol) and type 2 diabetes based on geographic region

CI, confidence interval; T2D, type 2 diabetes

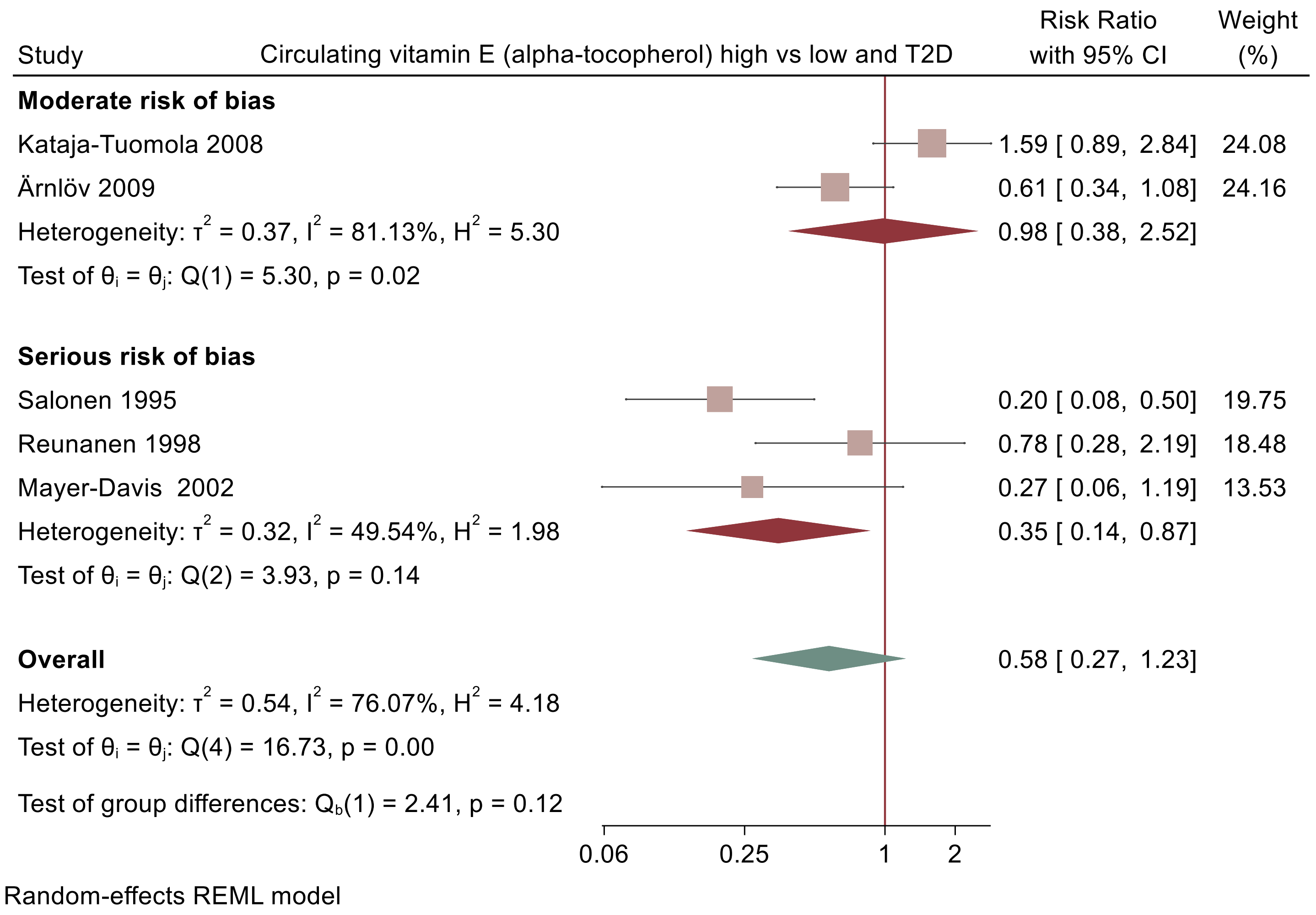

Supplementary Figure 26. Subgroup analysis for high vs low circulating vitamin E (alpha-tocopherol) and type 2 diabetes based on risk of bias

CI, confidence interval; T2D, type 2 diabetes

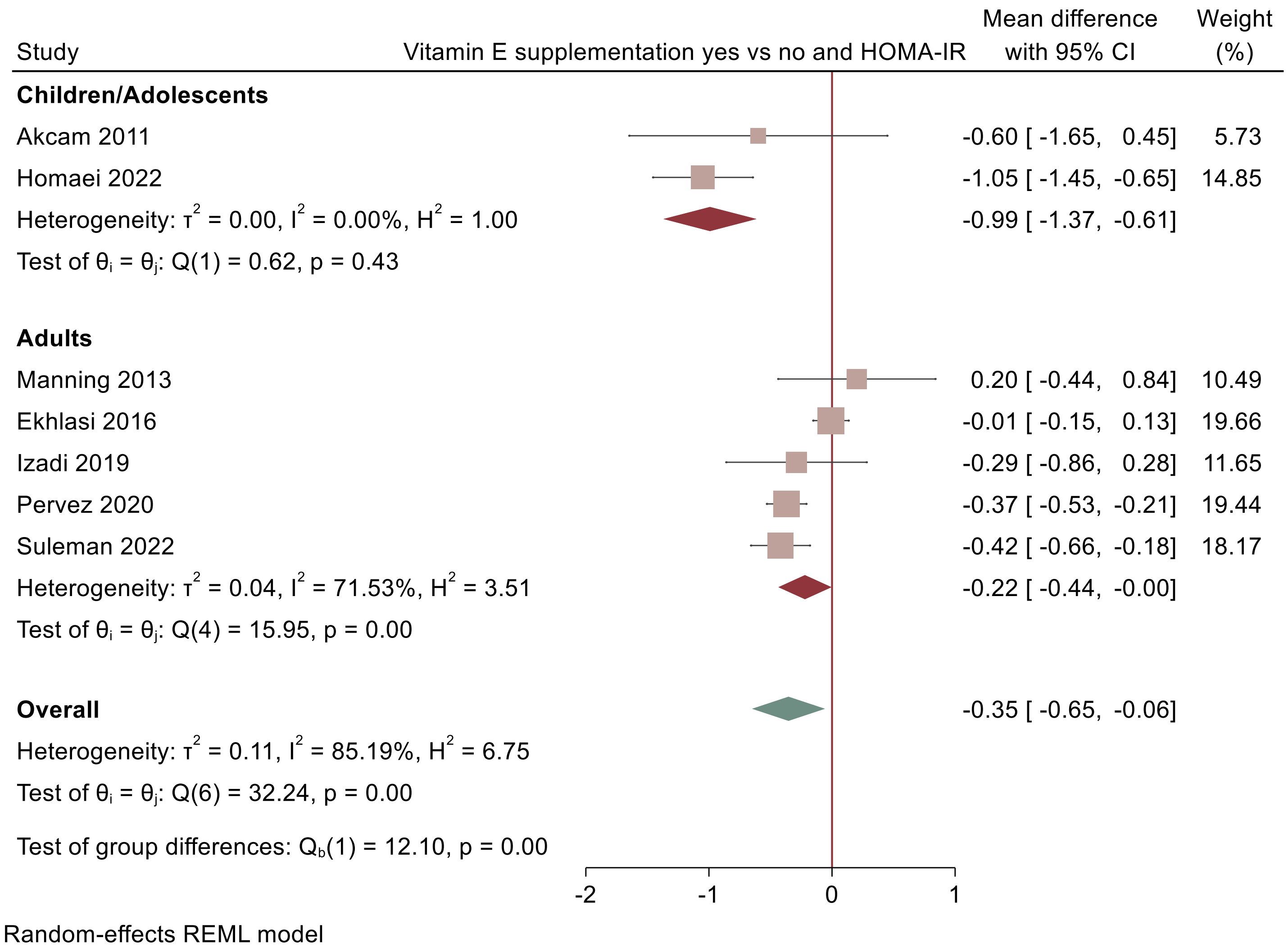

Supplementary Figure 27. Subgroup analysis for vitamin E supplementation vs placebo or lifestyle intervention and HOMA-IR based on age group

CI, confidence interval; HOMA-IR, homeostatic model assessment of insulin resistance

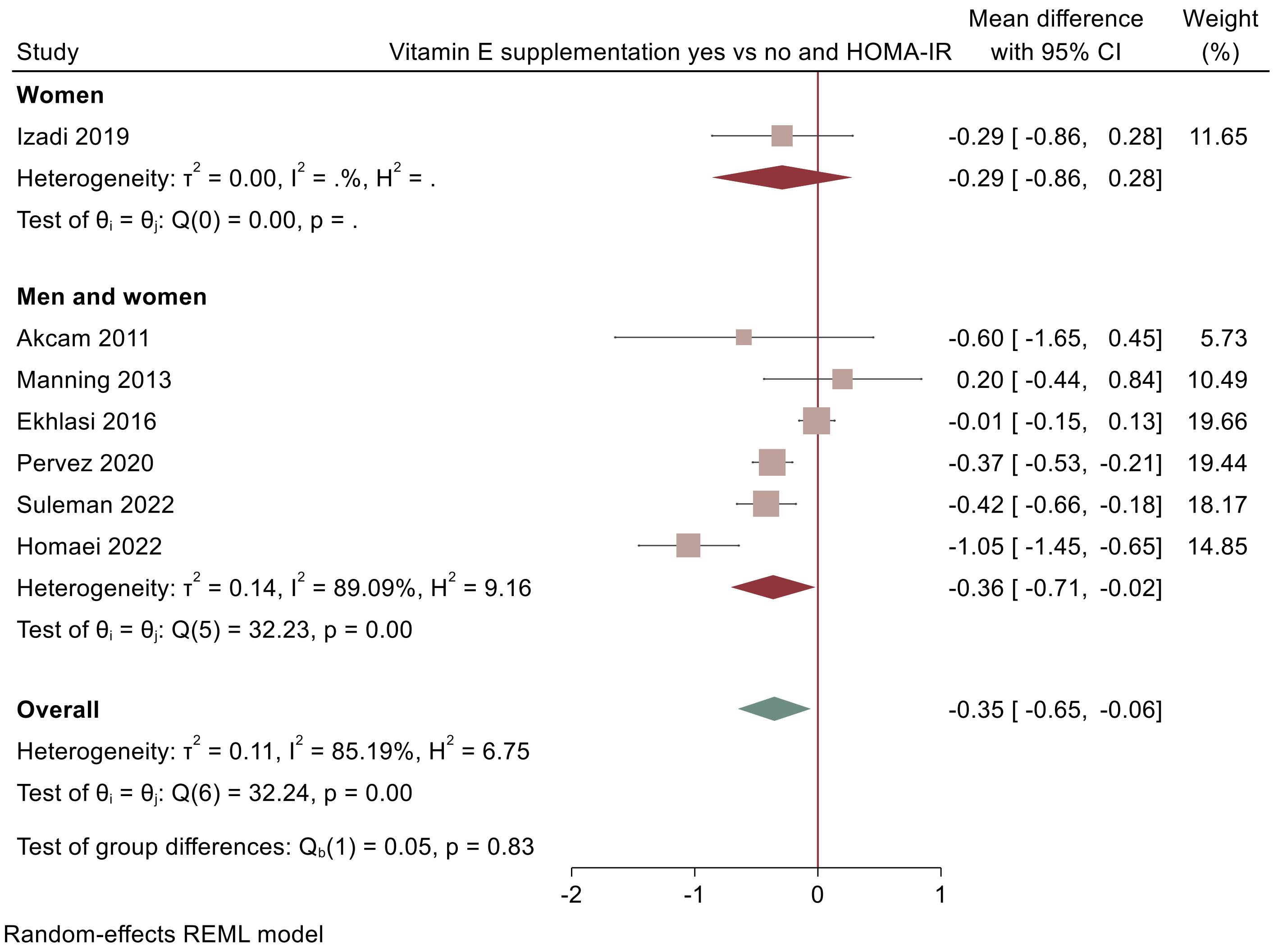

Supplementary Figure 28. Subgroup analysis for vitamin E supplementation vs placebo or lifestyle intervention and HOMA-IR based on sex

CI, confidence interval; HOMA-IR, homeostatic model assessment of insulin resistance

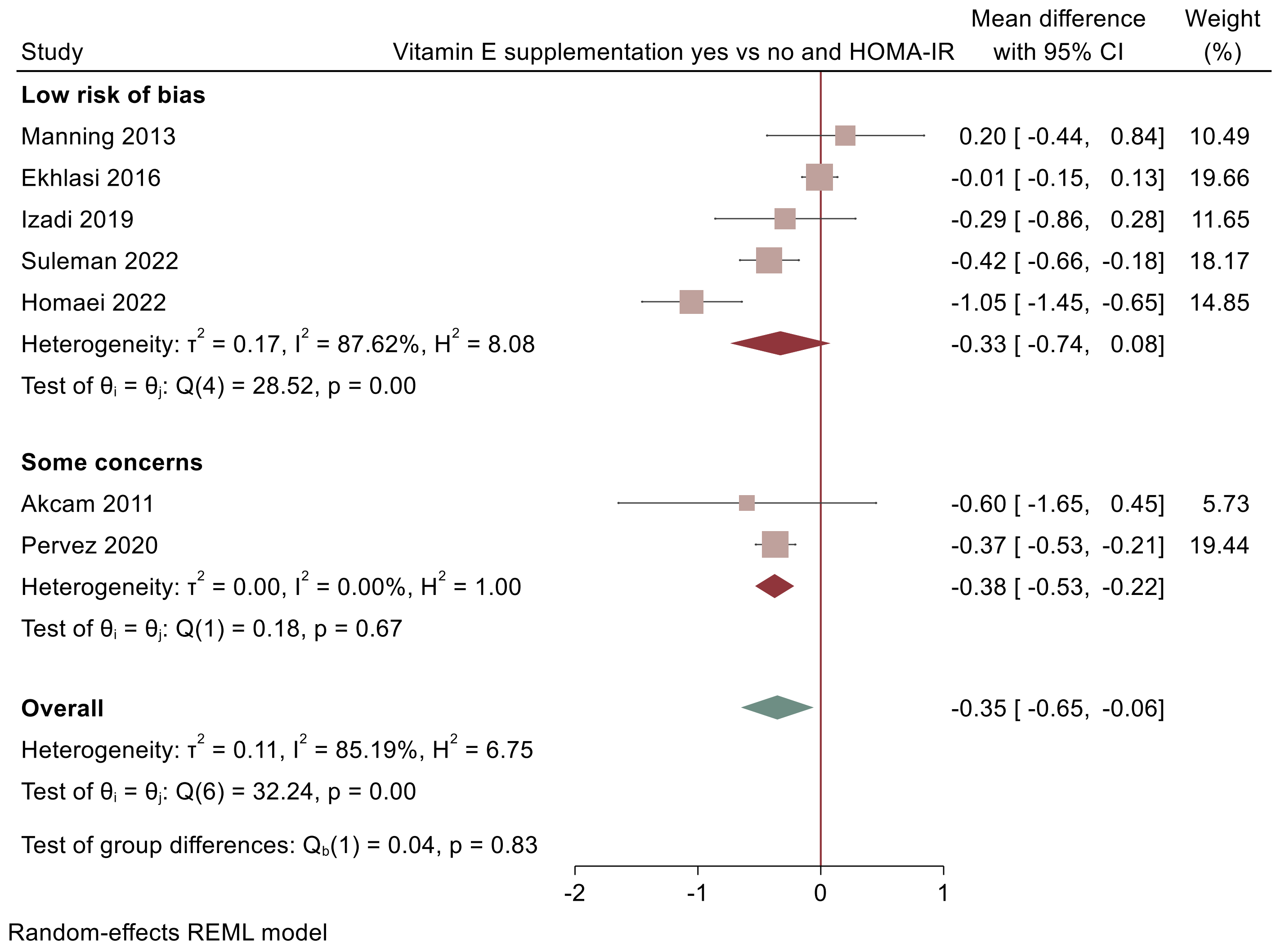

Supplementary Figure 29. Subgroup analysis for vitamin E supplementation vs placebo or lifestyle intervention and HOMA-IR based on risk of bias

CI, confidence interval; HOMA-IR, homeostatic model assessment of insulin resistance

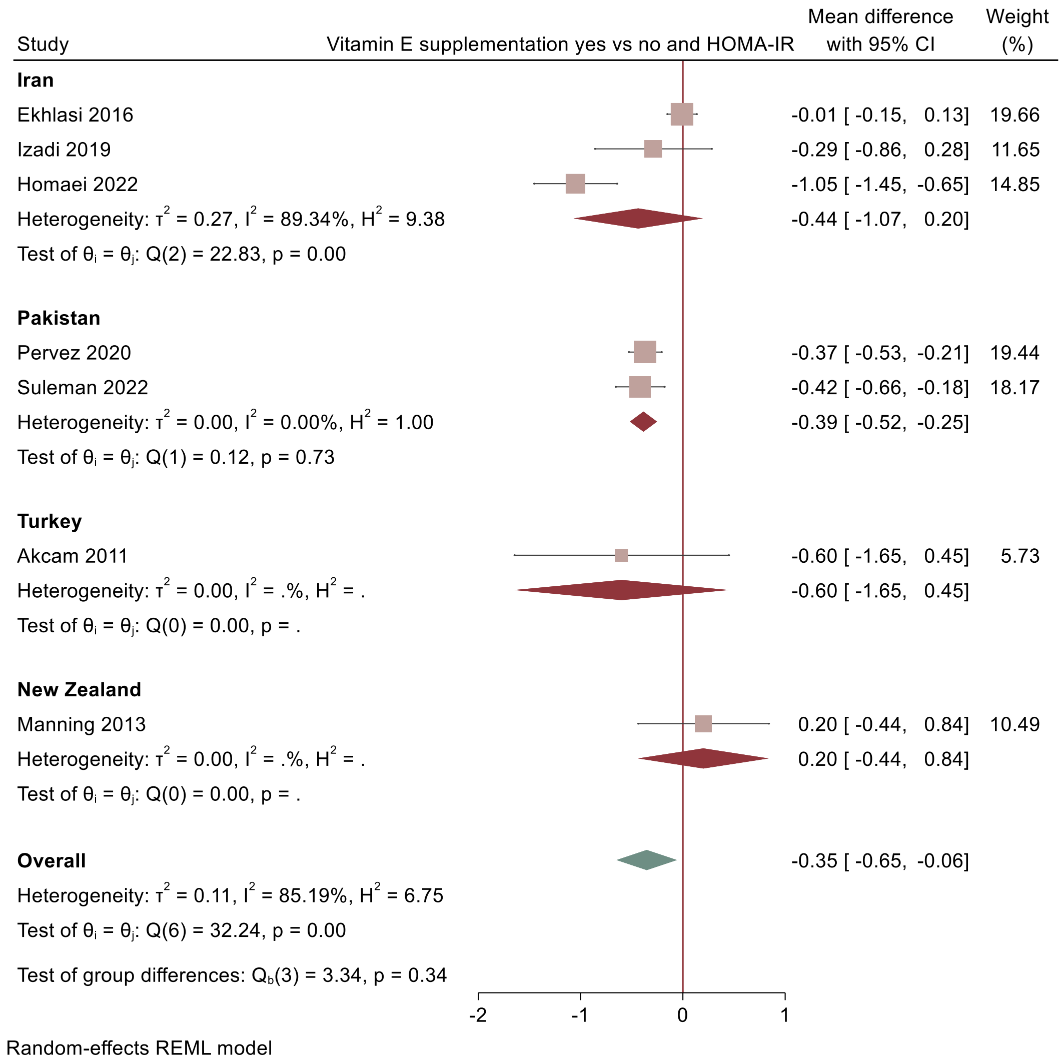

Supplementary Figure 30. Subgroup analysis for vitamin E supplementation vs placebo or lifestyle intervention and HOMA-IR based on geographic region

CI, confidence interval; HOMA-IR, homeostatic model assessment of insulin resistance

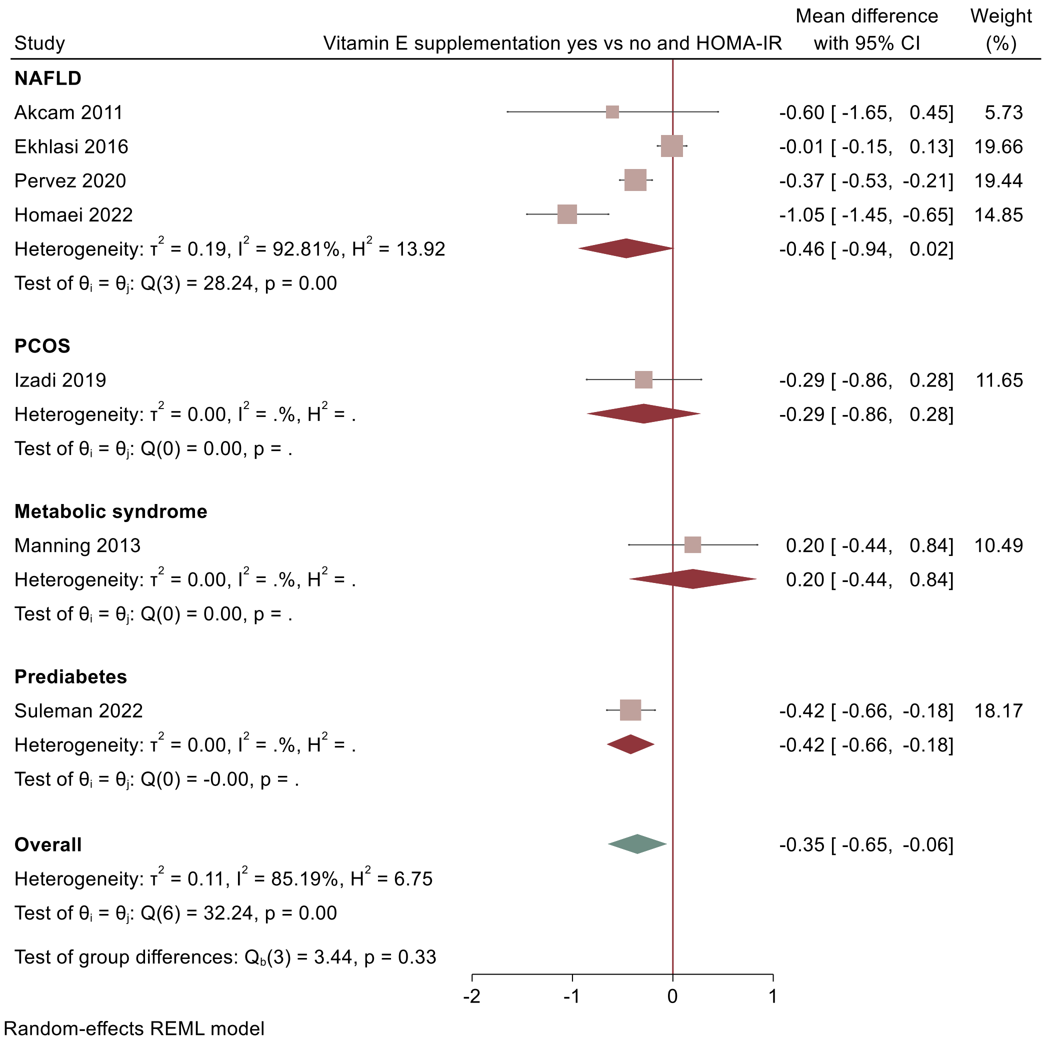

Supplementary Figure 31. Subgroup analysis for vitamin E supplementation vs placebo or lifestyle intervention and HOMA-IR based on health condition

CI, confidence interval; HOMA-IR, homeostatic model assessment of insulin resistance; NAFLD, non-alcoholic fatty liver disease; PCOS, polycystic ovary syndrome

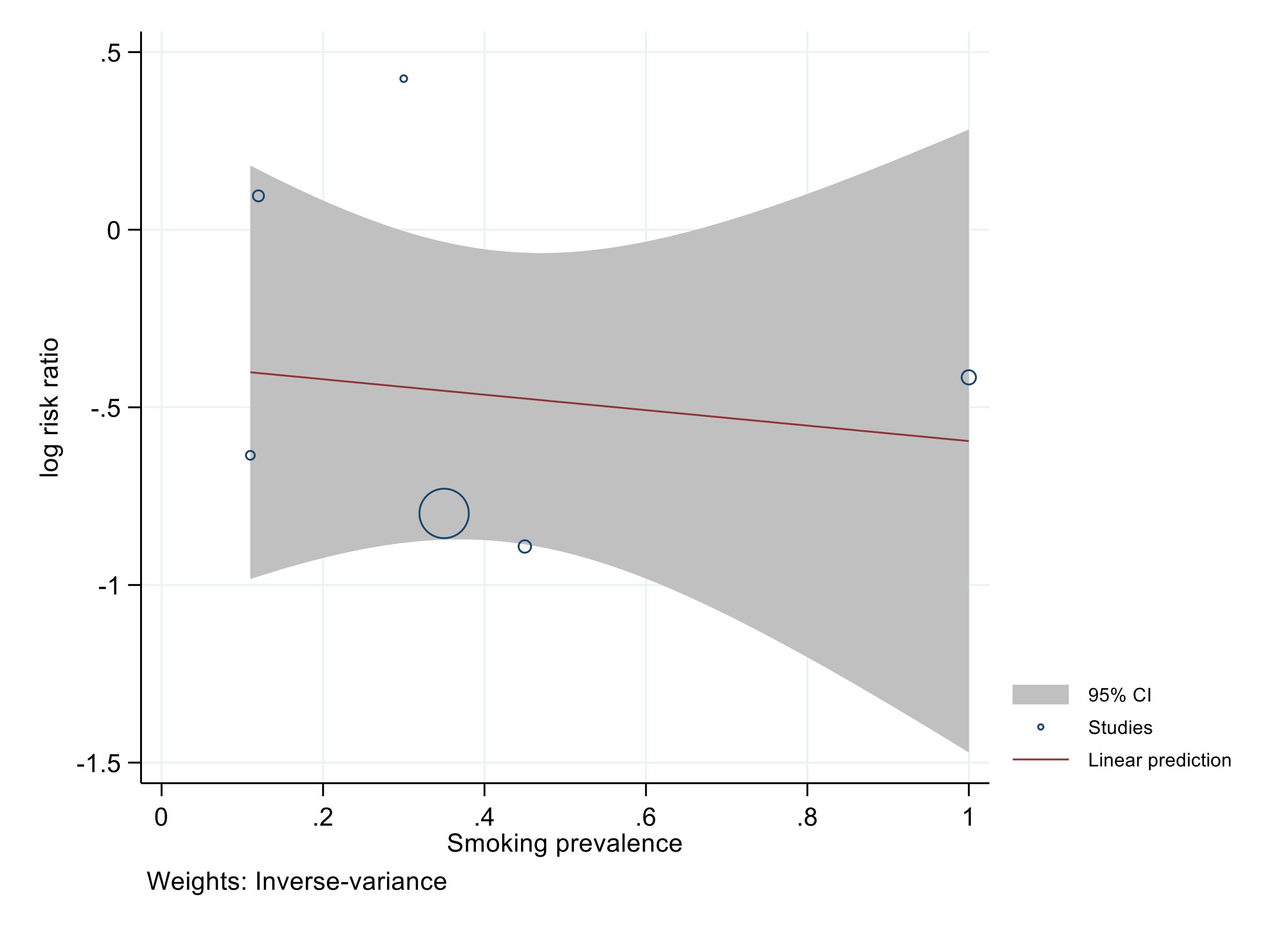

Supplementary Figure 32. Meta-regression for high vs low circulating beta-carotene and type 2 diabetes with smoking prevalence within the cohort as effect modifier (*p*=0.750)

CI, confidence interval

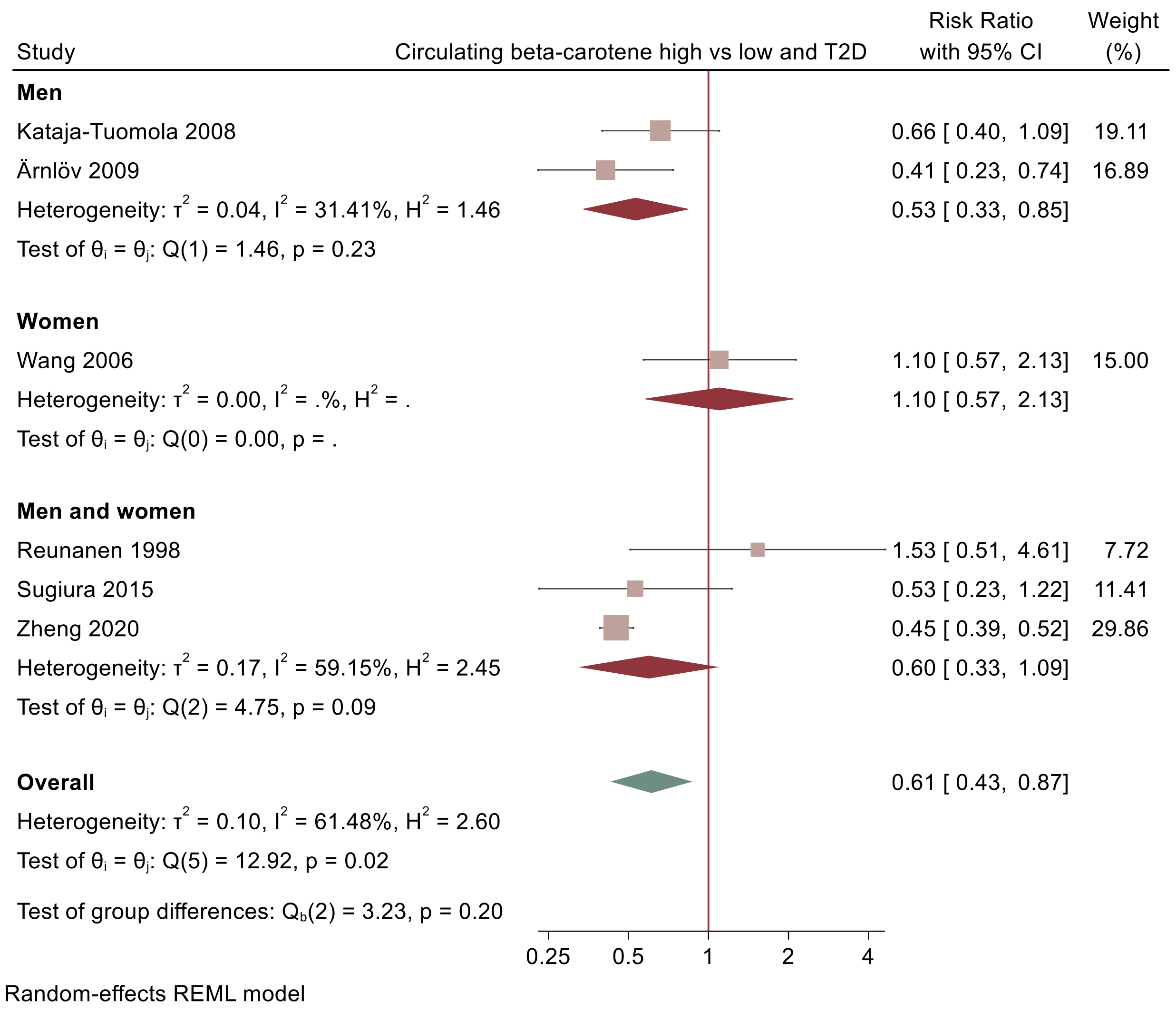

Supplementary Figure 33. Subgroup analysis for high vs low circulating beta-carotene and type 2 diabetes based on sex

CI, confidence interval; T2D, type 2 diabetes

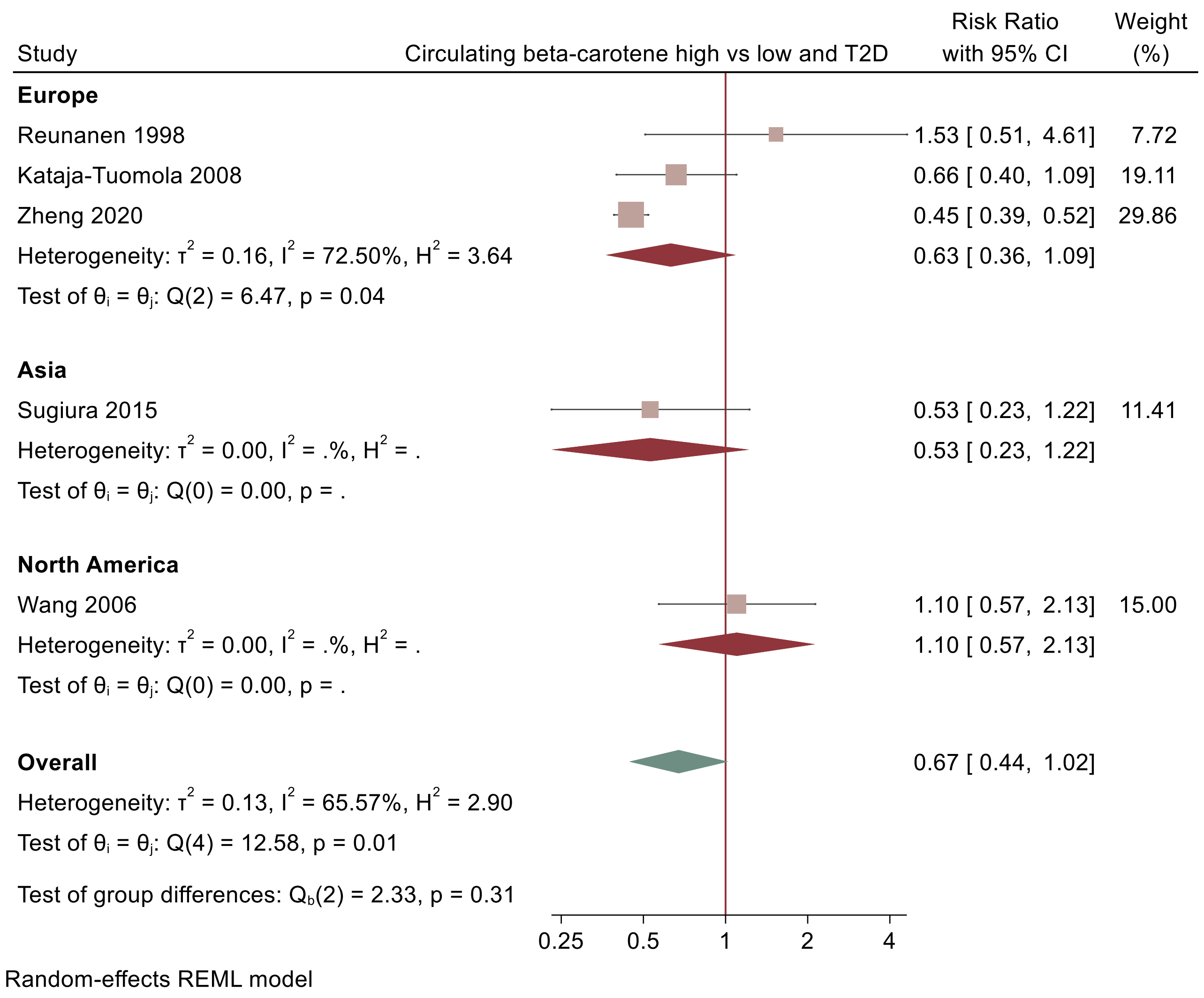

Supplementary Figure 34. Subgroup analysis for high vs low circulating beta-carotene and type 2 diabetes based on geographic region

CI, confidence interval; T2D, type 2 diabetes

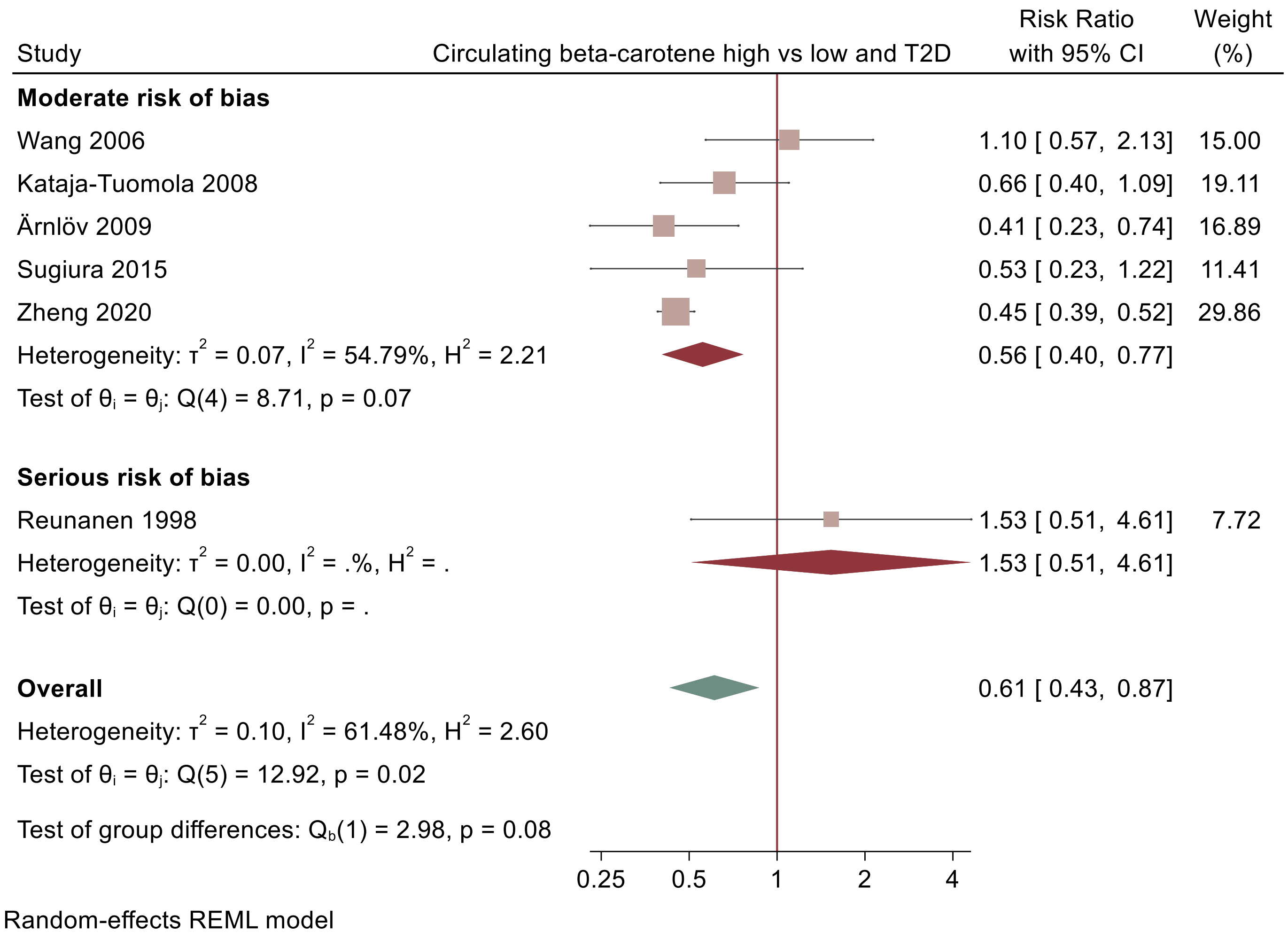

Supplementary Figure 35. Subgroup analysis for high vs low circulating beta-carotene and type 2 diabetes based on risk of bias

CI, confidence interval; T2D, type 2 diabetes

Supplementary references

1. Ärnlöv, J. *et al.* Serum and dietary β-carotene and α-tocopherol and incidence of type 2 diabetes mellitus in a community-based study of Swedish men: report from the Uppsala Longitudinal Study of Adult Men (ULSAM) study. *Diabetologia* **52**, 97–105 (2009).
2. Cooper, A. J. M. *et al.* The association between a biomarker score for fruit and vegetable intake and incident type 2 diabetes: the EPIC-Norfolk study. *Eur. J. Clin. Nutr.* **69**, 449–454 (2015).
3. de Oliveira Otto, M. C. *et al.* Dietary Intakes of Zinc and Heme Iron from Red Meat, but Not from Other Sources, Are Associated with Greater Risk of Metabolic Syndrome and Cardiovascular Disease3. *J. Nutr.* **142**, 526–533 (2012).
4. Eshak, E. S., Iso, H., Muraki, I. & Tamakoshi, A. Among the water-soluble vitamins, dietary intakes of vitamins C, B 2 and folate are associated with the reduced risk of diabetes in Japanese women but not men. *Br. J. Nutr.* **121**, 1357–1364 (2019).
5. Eshak, E. S., Iso, H., Muraki, I. & Tamakoshi, A. Fat-soluble vitamins from diet in relation to risk of type 2 diabetes mellitus in Japanese population. *Br. J. Nutr.* **121**, 647–653 (2019).
6. Harding, A.-H. *et al.* Plasma vitamin C level, fruit and vegetable consumption, and the risk of new-onset type 2 diabetes mellitus: the European prospective investigation of cancer--Norfolk prospective study. *Arch. Intern. Med.* **168**, 1493–1499 (2008).
7. Hozawa, A. *et al.* Associations of Serum Carotenoid Concentrations with the Development of Diabetes and with Insulin Concentration: Interaction with Smoking. *Am. J. Epidemiol.* **163**, 929–937 (2006).
8. Kataja-Tuomola, M. K., Kontto, J. P., Männistö, S., Albanes, D. & Virtamo, J. Intake of antioxidants and risk of type 2 diabetes in a cohort of male smokers. *Eur. J. Clin. Nutr.* **65**, 590–597 (2011).
9. Kataja-Tuomola, M. *et al.* Effect of α-tocopherol and β-carotene supplementation on the incidence of type 2 diabetes. *Diabetologia* **51**, 47–53 (2008).
10. Klein, E. A. *et al.* Vitamin E and the Risk of Prostate Cancer. *JAMA* **306**, 1549 (2011).
11. Dunn, B. K., Richmond, E. S., Minasian, L. M., Ryan, A. M. & Ford, L. G. A Nutrient Approach to Prostate Cancer Prevention: The Selenium and Vitamin E Cancer Prevention Trial (SELECT). *Nutr. Cancer* **62**, 896–918 (2010).
12. Lippman, S. M. *et al.* Effect of Selenium and Vitamin E on Risk of Prostate Cancer and Other Cancers. *JAMA* **301**, 39 (2009).
13. Liu, S. *et al.* Long-term β-Carotene Supplementation and Risk of Type 2 Diabetes Mellitus. *JAMA* **282**, 1073 (1999).
14. Liu, S. *et al.* Vitamin E and Risk of Type 2 Diabetes in the Women's Health Study Randomized Controlled Trial. *Diabetes* **55**, 2856–2862 (2006).
15. Mayer-Davis, E. J., Costacou, T., King, I., Zaccaro, D. J. & Bell, R. A. Plasma and Dietary Vitamin E in Relation to Incidence of Type 2 Diabetes. *Diabetes Care* **25**, 2172–2177 (2002).
16. Montonen, J., Knekt, P., Järvinen, R. & Reunanen, A. Dietary Antioxidant Intake and Risk of Type 2 Diabetes. *Diabetes Care* **27**, 362–366 (2004).
17. Prentice, R. L. *et al.* Application of blood concentration biomarkers in nutritional epidemiology: example of carotenoid and tocopherol intake in relation to chronic disease risk. *Am. J. Clin. Nutr.* **109**, 1189–1196 (2019).
18. Reunanen, A., Knekt, P., Aaran, R. K. & Aromaa, A. Serum antioxidants and risk of non-insulin dependent diabetes mellitus. *Eur. J. Clin. Nutr.* **52**, 89–93 (1998).
19. Salonen, J. T. *et al.* Increased risk of non-insulin dependent diabetes mellitus at low plasma vitamin E concentrations: a four year follow up study in men. *BMJ* **311**, 1124–1127 (1995).
20. Savolainen, O. *et al.* Biomarkers of food intake and nutrient status are associated with glucose tolerance status and development of type 2 diabetes in older Swedish women. *Am. J. Clin. Nutr.* **106**, 1302–1310 (2017).
21. Sluijs, I. *et al.* Dietary intake of carotenoids and risk of type 2 diabetes. *Nutr. Metab. Cardiovasc. Dis.* **25**, 376–381 (2015).
22. Song, Y., Cook, N. R., Albert, C. M., Van Denburgh, M. & Manson, J. E. Effects of vitamins C and E and β-carotene on the risk of type 2 diabetes in women at high risk of cardiovascular disease: a randomized controlled trial. *Am. J. Clin. Nutr.* **90**, 429–437 (2009).
23. Sugiura, M., Nakamura, M., Ogawa, K., Ikoma, Y. & Yano, M. High-serum carotenoids associated with lower risk for developing type 2 diabetes among Japanese subjects: Mikkabi cohort study. *BMJ Open Diabetes Res. Care* **3**, e000147 (2015).
24. Wang, L. *et al.* Plasma Lycopene, Other Carotenoids, and the Risk of Type 2 Diabetes in Women. *Am. J. Epidemiol.* **164**, 576–585 (2006).
25. Zheng, J.-S. *et al.* Association of plasma biomarkers of fruit and vegetable intake with incident type 2 diabetes: EPIC-InterAct case-cohort study in eight European countries. *BMJ* **370**, m2194 (2020).
26. Zheng, J.-S. *et al.* Plasma Vitamin C and Type 2 Diabetes: Genome-Wide Association Study and Mendelian Randomization Analysis in European Populations. *Diabetes Care* **44**, 98–106 (2021).
27. Zhou, C. *et al.* Dietary Vitamin C Intake Reduces the Risk of Type 2 Diabetes in Chinese Adults: HOMA-IR and T-AOC as Potential Mediators. *PLoS One* **11**, e0163571 (2016).
28. Blondin, S. A. *et al.* Serum Retinol and Carotenoids in Association with Biomarkers of Insulin Resistance among Premenopausal Women. *ISRN Nutr.* **2013**, 619516 (2013).
29. Xiao, M.-L. *et al.* Higher serum carotenoids associated with improvement of non-alcoholic fatty liver disease in adults: a prospective study. *Eur. J. Nutr.* **58**, 721–730 (2019).
30. He, Z. *et al.* Effects of Oral Vitamin C Supplementation on Liver Health and Associated Parameters in Patients With Non-Alcoholic Fatty Liver Disease: A Randomized Clinical Trial. *Front. Nutr.* **8**, 745609 (2021).
31. Akcam, M. *et al.* Therapeutic Effect of Metformin and Vitamin E Versus Prescriptive Diet in Obese Adolescents

with Fatty Liver. *Int. J. Vitam. Nutr. Res.* **81**, 398–406 (2011).
32. Ekhlasi, G. *et al.* Do symbiotic and Vitamin E supplementation have favorite effects in nonalcoholic fatty liver disease? A randomized, double-blind, placebo-controlled trial. *J. Res. Med. Sci.* **21**, 106 (2016).
33. Homaei, A., Alhadad, M., Arad, B. & Saffari, F. Effect of Metformin or Vitamin E on Ultrasonographic Grade and Biochemical Findings of Children and Adolescents with Nonalcoholic Fatty Liver Disease: A Randomized Clinical Trial. *J. Compr. Pediatr.* **13**, (2022).
34. Izadi, A. *et al.* Hormonal and Metabolic Effects of Coenzyme Q10 and/or Vitamin E in Patients with Polycystic Ovary Syndrome. *J. Clin. Endocrinol. Metab.* **104**, 319–327 (2018).
35. Manning, P. J. *et al.* The effect of lipoic acid and vitamin E therapies in individuals with the metabolic syndrome. *Nutr. Metab. Cardiovasc. Dis.* **23**, 543–549 (2013).
36. Pervez, M. A., Khan, D. A., Slehria, A. U. R. & Ijaz, A. Delta-tocotrienol supplementation improves biochemical markers of hepatocellular injury and steatosis in patients with nonalcoholic fatty liver disease: A randomized, placebo-controlled trial. *Complement. Ther. Med.* **52**, 102494 (2020).
37. Suleman, F., Khan, D. A., Pervez, M. A. & Aamir, M. Effects of delta-tocotrienol supplementation on glycaemic control in individuals with prediabetes: A randomized controlled study. *J. Pak. Med. Assoc.* **72**, 4–7 (2022).
38. Costacou, T., Ma, B., King, I. B. & Mayer-Davis, E. J. Plasma and dietary vitamin E in relation to insulin secretion and sensitivity. *Diabetes, Obes. Metab.* **10**, 223–228 (2008).
39. Chiang, Y.-F. *et al.* Dietary oxidised frying oil causes oxidative damage of pancreatic islets and impairment of insulin secretion, effects associated with vitamin E deficiency. *Br. J. Nutr.* **105**, 1311–1319 (18336BC).
40. McSorley, P. T., Young, I. S., Bell, P. M., Fee, J. P. H. & McCance, D. R. Vitamin C improves endothelial function in healthy estrogen-deficient postmenopausal women. *Climacteric* **6**, 238–247.
41. Agte, V. V, Nagmote, R. V & Tarwadi, K. V. Comparative in vitro uptake of zinc by erythrocytes of normal vs Type 2 diabetic individuals and the associated factors. *Diabetes. Nutr. Metab.* **17**, 343–349.
42. Yakaryilmaz, F. *et al.* Effects of vitamin E treatment on peroxisome proliferator-activated receptor-alpha expression and insulin resistance in patients with non-alcoholic steatohepatitis: results of a pilot study. *Intern. Med. J.* **37**, 229–235.
43. Paolisso, G. *et al.* Pharmacologic doses of vitamin E improve insulin action in healthy subjects and non-insulin-dependent diabetic patients. *Am. J. Clin. Nutr.* **57**, 650–656.
44. Villaca Chaves, G. *et al.* Serum retinol and beta-carotene levels and risk factors for cardiovascular disease in morbid obesity. *Int. J. Vitam. Nutr. Res.* **80**, 159–167.
45. Galvan, A. Q. *et al.* Insulin decreases circulating vitamin E levels in humans. *Metabolism.* **45**, 998–1003.
46. Chapple, I. L. C., Milward, M. R. & Dietrich, T. The prevalence of inflammatory periodontitis is negatively associated with serum antioxidant concentrations. *J. Nutr.* **137**, 657–664.
47. Rodriguez-Ramirez, G., Simental-Mendia, L. E., Carrera-Gracia, M. de la A. & Quintanar-Escorza, M.-A. Vitamin E Deficiency and Oxidative Status are Associated with Prediabetes in Apparently Healthy Subjects. *Arch. Med. Res.* **48**, 257–262.
48. Rizzo, M. R. *et al.* Evidence for anti-inflammatory effects of combined administration of vitamin E and C in older persons with impaired fasting glucose: impact on insulin action. *J. Am. Coll. Nutr.* **27**, 505–511.
49. Lovejoy, J. C. The impact of nuts on diabetes and diabetes risk. *Curr. Diab. Rep.* **5**, 379–384.
50. McComsey, G., Southwell, H., Gripshover, B., Salata, R. & Valdez, H. Effect of antioxidants on glucose metabolism and plasma lipids in HIV-infected subjects with lipoatrophy. *J. Acquir. Immune Defic. Syndr.* **33**, 605–607.
51. Alexander, H., Lockwood, L. P., Harris, M. A. & Melby, C. L. Risk factors for cardiovascular disease and diabetes in two groups of Hispanic Americans with differing dietary habits. *J. Am. Coll. Nutr.* **18**, 127–136.
52. Hirashima, O. *et al.* Improvement of endothelial function and insulin sensitivity with vitamin C in patients with coronary spastic angina: possible role of reactive oxygen species. *J. Am. Coll. Cardiol.* **35**, 1860–1866.
53. Alho, H., Leinonen, J. S., Erhola, M., Lonnrot, K. & Aejmelaeus, R. Assay of antioxidant capacity of human plasma and CSF in aging and disease. *Restor. Neurol. Neurosci.* **12**, 159–165.
54. Facchini, F., Coulston, A. M. & Reaven, G. M. Relation between dietary vitamin intake and resistance to insulin-mediated glucose disposal in healthy volunteers. *Am. J. Clin. Nutr.* **63**, 946–949.
55. Kostecka, M. Eating habits of preschool children and the risk of obesity, insulin resistance and metabolic syndrome in adults. *Pakistan J. Med. Sci.* **30**, 1299–1303.
56. Bo, S. *et al.* Gestational hyperglycemia, zinc, selenium, and antioxidant vitamins. *Nutrition* **21**, 186–191.
57. Caballero, B. Vitamin E improves the action of insulin. *Nutr. Rev.* **51**, 339–340.
58. Gray, B., Swick, J. & Ronnenberg, A. G. Vitamin E and adiponectin: proposed mechanism for vitamin E-induced improvement in insulin sensitivity. *Nutr. Rev.* **69**, 155–161.
59. Duc Son, L. N. T. *et al.* Anthropometric characteristics, dietary patterns and risk of type 2 diabetes mellitus in Vietnam. *J. Am. Coll. Nutr.* **24**, 229–234 (2005).
60. Actrn. Randomised, double-blind, placebo controlled phase II study of the efficacy of Phospha-E biomarkers of inflammation, in patients with metabolic syndrome and mild to moderate hyperlipidemia. *https://trialsearch.who.int/Trial2.aspx?TrialID=ACTRN12607000343404* (2007).
61. Andersen, G., Koehler, P. & Somoza, V. Postprandial glucose and free fatty acid response is improved by wheat bread fortified with germinated wheat seedlings. *Curr. Top. Nutraceutical Res.* **6**, 15–21 (2008).
62. England, C. Y., Coulman, K. D., Gorton, J. G., Paxton, E. C. & Andrews, R. C. Does a diagnosis of Type 2 diabetes lead to a change in diet? *Diabet. Med.* **26**, 171- (2009).
63. Paolisso, G. *et al.* Plasma vitamin C affects glucose homeostasis in healthy subjects and in non-insulin-dependent diabetics. *Am. J. Physiol.* **266**, E261-8.

64. LEE, H. J., KIM, H. C., VITEK, L. & NAM, C. M. Algae Consumption and Risk of Type 2 Diabetes: Korean National Health and Nutrition Examination Survey in 2005. *J. Nutr. Sci. Vitaminol. (Tokyo).* **56**, 13–18 (2010).
65. Tsitouras, P. D. & Traustadóttir, T. Role of exercise and dietary supplements in the management of prediabetes and type 2 diabetes. *Clin. Geriatr.* **18**, 22–27 (2010).
66. Ansari, M. A., Ansari, S. & Memon, Z. Does antioxidant ascorbic acid supplementation delay lung function deterioration in stable patients with chronic obstructive? Pulmonary disease. *Rawal Med. J.* **35**, 133-136 (2010).
67. Cene, C. W. & Pignone, M. The effect of fruit and vegetable intake on the incidence of diabetes. *Clin. Diabetes* **29**, 113–115 (2011).
68. D'Adamo, E. *et al.* Improved oxidative stress and insulin sensitivity in obese prepubertal children with liver steatosis treated with vitamin E. *Horm. Res. Paediatr.* **78**, 75 (2012).
69. Actrn. Fruit and vegetable supplement study in obese adults aged 50 years or older. *https://trialsearch.who.int/Trial2.aspx?TrialID=ACTRN12614000079640* (2014).
70. A Mendelian randomization study of circulating uric acid and type 2 diabetes. *Diabetes. 64 (pp 3028-3036), 2015. Date Publ. august 2015.* (2015) doi:10.2337/db14-0742.
71. Ros, E. Nuts and CVD. *Br. J. Nutr.* **113**, S111--S120 (2015).
72. Kiss, R. *et al.* Insulin-Sensitizer Effects of Fenugreek Seeds in Parallel with Changes in Plasma MCH Levels in Healthy Volunteers. *Int. J. Mol. Sci.* **19**, 771 (2018).
73. Ghanwat, G. H. & Sontakke, A. V. Effect of Vitamin C supplementation on insulin resistance, β-cell function and insulin sensitivity in obese and non obese individuals. *Indian J. Public Heal. Res. Dev.* **10**, 183–188 (2019).
74. Hirai, N. *et al.* Insulin resistance and endothelial dysfunction in smokers: effects of vitamin C. *Am. J. Physiol. Heart Circ. Physiol.* **279**, H1172-8.
75. Fan, C. *et al.* Circulating vitamin E and cardiometabolic measures: A Mendelian randomization analysis. *J. Clin. Biochem. Nutr.* **65**, 160–169 (2019).
76. Bi, X., Loo, Y. T., Yeo, P. L. Q. & Henry, C. J. Are α-tocopherol levels associated with improved glycaemia? *J. Nutr. Intermed. Metab.* **18**, (2019).
77. Abdel-Maboud, M. *et al.* The efficacy of vitamin E in reducing non-alcoholic fatty liver disease: a systematic review, meta-analysis, and meta-regression. *Therap. Adv. Gastroenterol.* **13**, (2020).
78. Toz, H. T. & Gozke, E. Investigation of insulin resistance and vitamin e deficiency in chronic inflammatory demyelinatıng polyneuropathy: A 5-year retrospective study. *Neurol. Sci. Neurophysiol.* **37**, 24–28 (2020).
79. Harari, A. *et al.* Obesity and Insulin Resistance Are Inversely Associated with Serum and Adipose Tissue Carotenoid Concentrations in Adults. *J. Nutr.* **150**, 38–46 (2020).
80. Actrn. Micronutrient supplementation in metabolic syndrome. *https://trialsearch.who.int/Trial2.aspx?TrialID=ACTRN12621000678897* (2021).
81. Alduraywish, A. A. Cardiorespiratory and metabolic fitness indicators in novice volleyball trainees: effect of 1-week antioxidant supplementation with N-acetyl-cysteine/zinc/vitamin C. *J. Int. Med. Res.* **49**, 3000605211067125 (2021).
82. Afreeth, S. U. M., Dheepthi, M., Dhanisha, S. S., Sowmya, C. & Kaleeswari, R. Antioxidants And Their Potential Role In Diabetes. *Res. J. Pharm. Biol. Chem. Sci.* **12**, 43–50 (2021).
83. Kardas, F. *et al.* Evaluation of micronutrient levels in children and adolescents with obesity and their correlation with the components of metabolic syndrome. *Turk. J. Pediatr.* **63**, 48–58 (2021).
84. Crosby, L. *et al.* Changes in Food and Nutrient Intake and Diet Quality on a Low-Fat Vegan Diet Are Associated with Changes in Body Weight, Body Composition, and Insulin Sensitivity in Overweight Adults: A Randomized Clinical Trial. *J. Acad. Nutr. Diet.* (2022) doi:https://dx.doi.org/10.1016/j.jand.2022.04.008.
85. Fang, F., Kang, Z. & Wong, C. Vitamin E tocotrienols improve insulin sensitivity through activating peroxisome proliferator-activated receptors. *Mol. Nutr. Food Res.* **54**, 345–352.
86. Li, M. C. Associations between Adherence to the Taiwan Dietary Reference Intakes of Micronutrients and the Risk of Type 2 Diabetes. *Int. J. Environ. Res. Public Health* **19**, (2022).
87. Sun, H., Karp, J., Sun, K. M. & Weaver, C. M. Decreasing Vitamin C Intake, Low Serum Vitamin C Level and Risk for US Adults with Diabetes. *Nutrients* **14**, 21 (2022).
88. Actrn. Elucidating the Effects of Tocotrienol rich Vitamin E on Metabolic Biomarkers in a Pre-diabetes Population of different ethnicities in Malaysia. *https://trialsearch.who.int/Trial2.aspx?TrialID=ACTRN12622000256774* (2022).
89. Shin, M.-J., Park, E., Lee, J. H. & Chung, N. Relationship between insulin resistance and lipid peroxidation and antioxidant vitamins in hypercholesterolemic patients. *Ann. Nutr. Metab.* **50**, 115–120 (2795BC).
90. Villaca Chaves, G., Pereira, S. E., Saboya, C. J. & Ramalho, A. Non-alcoholic fatty liver disease and its relationship with the nutritional status of vitamin A in individuals with class III obesity. *Obes. Surg.* **18**, 378–385 (26193).
91. Chalasani, N. P. *et al.* Pioglitazone versus vitamin E versus placebo for the treatment of non-diabetic patients with non-alcoholic steatohepatitis: PIVENS trial design. *Contemp. Clin. Trials* **30**, 88–96 (26894).
92. Bovet, P. *et al.* Divergent fifteen-year trends in traditional and cardiometabolic risk factors of cardiovascular diseases in the Seychelles. *Cardiovasc. Diabetol.* **8**, 34 (28926BC).
93. Beydoun, M. A. *et al.* Serum antioxidant status is associated with metabolic syndrome among U.S. adults in recent national surveys. *J. Nutr.* **141**, 903–913 (9222BC).
94. Mehmetoglu, I., Yerlikaya, F. H. & Kurban, S. Correlation between vitamin A, E, coenzyme Q(10) and degree of insulin resistance in obese and non-obese subjects. *J. Clin. Biochem. Nutr.* **49**, 159–163 (8547BC).
95. Denny-Brown, S., Stanley, T. L., Grinspoon, S. K. & Makimura, H. The association of macro- and micronutrient intake with growth hormone secretion. *Growth Horm. IGF Res.* **22**, 102–107 (849).
96. de Oliveira e Silva, L. G. *et al.* Relationship of the nutritional status of vitamin A and the regression of hepatic steatosis after Roux-en-Y gastric bypass surgery for treatment of class III obesity. *Arq. Bras. Cir. Dig.* **25**, 250–256.

97. D'Adamo, E. *et al.* Improved oxidative stress and cardio-metabolic status in obese prepubertal children with liver steatosis treated with lifestyle combined with Vitamin E. *Free Radic. Res.* **47**, 146–153 (10557).
98. Patel, C. J., Chen, R., Kodama, K., Ioannidis, J. P. A. & Butte, A. J. Systematic identification of interaction effects between genome- and environment-wide associations in type 2 diabetes mellitus. *Hum. Genet.* **132**, 495–508 (10568).
99. Suarez, E. C. & Schramm-Sapyta, N. L. Race differences in the relation of vitamins A, C, E, and beta-carotene to metabolic and inflammatory biomarkers. *Nutr. Res.* **34**, 1–10 (11462).
100. Higuchi, K. *et al.* Associations of serum beta-carotene and retinol concentrations with insulin resistance: the Toon Health Study. *Nutrition* **31**, 975–980 (30772).
101. Yki-Jarvinen, H. Nutritional Modulation of Non-Alcoholic Fatty Liver Disease and Insulin Resistance. *Nutrients* **7**, 9127–9138 (31553).
102. Olofsson, C. *et al.* Changes in fruit, vegetable and juice consumption after the diagnosis of type 2 diabetes: a prospective study in men. *Br. J. Nutr.* **117**, 712–719 (24375BC).
103. Azzini, E. *et al.* Effect of Red Orange Juice Consumption on Body Composition and Nutritional Status in Overweight/Obese Female: A Pilot Study. *Oxid. Med. Cell. Longev.* **2017**, 1672567 (14768BC).
104. Kollerits, B. *et al.* Plasma Concentrations of Afamin Are Associated With Prevalent and Incident Type 2 Diabetes: A Pooled Analysis in More Than 20,000 Individuals. *Diabetes Care* **40**, 1386–1393 (14182BC).
105. Quansah, D. Y. *et al.* Associations of Dietary Antioxidants and Risk of Type 2 Diabetes: Data from the 2007-2012 Korea National Health and Nutrition Examination Survey. *Molecules* **22**, (14083BC).
106. Wilson, R. *et al.* SunGold Kiwifruit Supplementation of Individuals with Prediabetes Alters Gut Microbiota and Improves Vitamin C Status, Anthropometric and Clinical Markers. *Nutrients* **10**, (4376BC).
107. Tran, D. T., Jorm, L. R., Johnson, M., Bambrick, H. & Lujic, S. Prevalence and risk factors of type 2 diabetes in older Vietnam-born Australians. *J. Community Health* **39**, 99–107.
108. Castellanos-Gutierrez, A., Sanchez-Pimienta, T. G., Carriquiry, A., da Costa, T. H. M. & Ariza, A. C. Higher dietary magnesium intake is associated with lower body mass index, waist circumference and serum glucose in Mexican adults. *Nutr. J.* **17**, 114 (3883BC).
109. Tobias, T. A. M., Wood, L. G. & Rastogi, D. Carotenoids, fatty acids and disease burden in obese minority adolescents with asthma. *Clin. Exp. Allergy* **49**, 838–846 (5337).
110. Wisgerhof, W. *et al.* Phenotypic and lifestyle determinants of HbA1c in the general population-The Hoorn Study. *PLoS One* **15**, e0233769 (15516).
111. Jiang, L. *et al.* Omega-3 fatty acids plus vitamin for women with gestational diabetes or prediabetes: a meta-analysis of randomized controlled studies. *J. Matern. Fetal. Neonatal Med.* **35**, 3135–3142 (15835).
112. Luo, J. *et al.* Urinary oxidized, but not enzymatic vitamin E metabolites are inversely associated with measures of glucose homeostasis in middle-aged healthy individuals. *Clin. Nutr.* **40**, 4192–4200 (25115).
113. Ewers, B., Sorensen, M. R., Fagt, S., Diaz, L. J. & Vilsboll, T. Intention and Perceptions of Healthy Eating versus Actual Intake Among Patients with Type 1 and Type 2 Diabetes and the General Population. *Patient Prefer. Adherence* **15**, 2027–2037 (25826).
114. Nguyen, H. D., Oh, H. & Kim, M.-S. Higher intakes of nutrients are linked with a lower risk of cardiovascular diseases, type 2 diabetes mellitus, arthritis, and depression among Korean adults. *Nutr. Res.* **100**, 19–32 (26130).
115. Piyathilake, C. J., Badiga, S., Hernandez, A., Brill, I. K. & Jolly, P. E. The consumption of micronutrients in relation to calorie intake and risk of insulin resistance. *Nutr. Metab. Cardiovasc. Dis.* **32**, 1385–1391 (30401BC).
116. Basu, A., Alman, A. C. & Snell-Bergeon, J. K. Associations of Dietary Antioxidants with Glycated Hemoglobin and Insulin Sensitivity in Adults with and without Type 1 Diabetes. *J. Diabetes Res.* **2022**, 4747573 (29999BC).
117. Musso, G. *et al.* Dietary habits and their relations to insulin resistance and postprandial lipemia in nonalcoholic steatohepatitis. *Hepatology* **37**, 909–916.
118. Tavridou, A., Unwin, N. C., Laker, M. F., White, M. & Alberti, K. G. Serum concentrations of vitamins A and E in impaired glucose tolerance. *Clin. Chim. Acta.* **266**, 129–140.
119. Morimoto, A., Ohno, Y., Tatsumi, Y., Mizuno, S. & Watanabe, S. Effects of healthy dietary pattern and other lifestyle factors on incidence of diabetes in a rural Japanese population. *Asia Pac. J. Clin. Nutr.* **21**, 601–608.
120. Schulze, M. B. *et al.* Glycemic index, glycemic load, and dietary fiber intake and incidence of type 2 diabetes in younger and middle-aged women. *Am. J. Clin. Nutr.* **80**, 348–356.
121. Aller, R. *et al.* Effect of silymarin plus vitamin E in patients with non-alcoholic fatty liver disease. A randomized clinical pilot study. *Eur. Rev. Med. Pharmacol. Sci.* **19**, 3118–3124.
122. Pervez, M. A. *et al.* Comparison of delta-tocotrienol and alpha-tocopherol effects on hepatic steatosis and inflammatory biomarkers in patients with non-alcoholic fatty liver disease: A randomized double-blind active-controlled trial. *Complement. Ther. Med.* **70**, 102866 (2022).
123. Hosseini-Esfahani, F., Beheshti, N., Koochakpoor, G., Mirmiran, P. & Azizi, F. Meat Food Group Intakes and the Risk of Type 2 Diabetes Incidence. *Front. Nutr.* **9**, 891111 (2022).
124. Gao, M. *et al.* Associations Between Dietary Patterns and Incident Type 2 Diabetes: Prospective Cohort Study of 120,343 UK Biobank Participants. *Diabetes Care* **45**, 1315–1325 (2022).
125. Lopes, T. *et al.* Consumption of Plant Foods and Its Association with Cardiovascular Disease Risk Profile in South Africans at High-Risk of Type 2 Diabetes Mellitus. *Int. J. Environ. Res. Public Heal. [Electronic Resour.* **19**, 14 (2022).
126. Tan, L. J., Hwang, S. B., Jun, S., Joung, H. & Shin, S. Dietary antioxidant consumption and the risk of type 2 diabetes in South Korean adults: a prospective cohort study based on the Health Examinees study. *BMJ Open* **12**, (2022).
127. Smith, D. L. *et al.* French-fried potato consumption and energy balance: a randomized controlled trial. *Am. J. Clin.*

Nutr. **115**, 1626–1636 (2022).
128. Smith, E. *et al.* A healthy dietary metabolic signature is associated with a lower risk for type 2 diabetes and coronary artery disease. *BMC Med.* **20**, (2022).
129. Li, L. *et al.* Whole fresh fruit intake and risk of incident diabetes in different glycemic stages: a nationwide prospective cohort investigation. *Eur. J. Nutr.* **62**, 771–782 (2023).
130. Liu, Z., Huang, H., Xie, J. & Xu, C. Dietary Patterns and Long-Term Outcomes in Patients with NAFLD: A Prospective Analysis of 128,695 UK Biobank Participants. *Nutrients* **15**, (2023).
131. Niu, K. *et al.* The dose-response relationship of fruit and vegetable intake and risk of type 2 diabetes among rural China: The Henan Rural Cohort study. *Prim. Care Diabetes* **17**, 161–167 (2023).
132. Partula, V. *et al.* Associations between consumption of dietary fibers and the risk of cardiovascular diseases, cancers, type 2 diabetes, and mortality in the prospective NutriNet-Sante cohort. *Am. J. Clin. Nutr.* **112**, 195–207.
133. Sobiecki, J. G. *et al.* A nutritional biomarker score of the Mediterranean diet and incident type 2 diabetes: Integrated analysis of data from the MedLey randomised controlled trial and the EPIC-InterAct case-cohort study. *PLoS Med. / Public Libr. Sci.* **20**, e1004221 (2023).
134. Gariballa, S., Al-Bluwi, G. S. M. & Yasin, J. Increased Fruit and Vegetable Consumption Mitigates Oxidative Damage and Associated Inflammatory Response in Obese Subjects Independent of Body Weight Change. *Nutrients* **15**, 28 (2023).
135. Kosti, R. I. *et al.* The association of specific types of vegetables consumption with 10-year type II diabetes risk: Findings from the ATTICA cohort study. *J. Hum. Nutr. Diet.* **36**, 226–240 (2023).
136. Panagiotakos, D. B. *et al.* The relationship between dietary habits, blood glucose and insulin levels among people without cardiovascular disease and type 2 diabetes; the ATTICA study. *Rev. Diabet. Stud.* **2**, 208–215 (6194).
137. Bazzano, L. A., Li, T. Y., Joshipura, K. J. & Hu, F. B. Intake of fruit, vegetables, and fruit juices and risk of diabetes in women. *Diabetes Care* **31**, 1311–1317 (26388).
138. Meinhold, C. L. *et al.* Predictors of fasting serum insulin and glucose and the risk of pancreatic cancer in smokers. *Cancer Causes Control* **20**, 681–690 (27197).
139. Wannamethee, S. G., Whincup, P. H., Thomas, M. C. & Sattar, N. Associations between dietary fiber and inflammation, hepatic function, and risk of type 2 diabetes in older men: potential mechanisms for the benefits of fiber on diabetes risk. *Diabetes Care* **32**, 1823–1825 (28829BC).
140. Kochar, J., Gaziano, J. M. & Djousse, L. Nut consumption and risk of type II diabetes in the Physicians' Health Study. *Eur. J. Clin. Nutr.* **64**, 75–79 (28636BC).
141. Sluijs, I. *et al.* Dietary intake of total, animal, and vegetable protein and risk of type 2 diabetes in the European Prospective Investigation into Cancer and Nutrition (EPIC)-NL study. *Diabetes Care* **33**, 43–48 (28539BC).
142. Bobeuf, F., Labonte, M., Khalil, A. & Dionne, I. J. Effects of resistance training combined with antioxidant supplementation on fat-free mass and insulin sensitivity in healthy elderly subjects. *Diabetes Res. Clin. Pract.* **87**, e1-3 (28451BC).
143. Silveira, J. Q., Dourado, G. K. Z. S. & Cesar, T. B. Red-fleshed sweet orange juice improves the risk factors for metabolic syndrome. *Int. J. Food Sci. Nutr.* **66**, 830–836.
144. Hopping, B. N. *et al.* Dietary fiber, magnesium, and glycemic load alter risk of type 2 diabetes in a multiethnic cohort in Hawaii. *J. Nutr.* **140**, 68–74 (28448BC).
145. Casas-Agustench, P. *et al.* Effects of one serving of mixed nuts on serum lipids, insulin resistance and inflammatory markers in patients with the metabolic syndrome. *Nutr. Metab. Cardiovasc. Dis.* **21**, 126–135 (28330BC).
146. Birlouez-Aragon, I. *et al.* A diet based on high-heat-treated foods promotes risk factors for diabetes mellitus and cardiovascular diseases. *Am. J. Clin. Nutr.* **91**, 1220–1226 (19228BC).
147. McCall, D. O. *et al.* The effect of increased dietary fruit and vegetable consumption on endothelial activation, inflammation and oxidative stress in hypertensive volunteers. *Nutr. Metab. Cardiovasc. Dis.* **21**, 658–664 (19139BC).
148. Kalgaonkar, S. *et al.* Differential effects of walnuts vs almonds on improving metabolic and endocrine parameters in PCOS. *Eur. J. Clin. Nutr.* **65**, 386–393 (18337BC).
149. Yfanti, C. *et al.* Effect of antioxidant supplementation on insulin sensitivity in response to endurance exercise training. *Am. J. Physiol. Endocrinol. Metab.* **300**, E761-70 (9337BC).
150. Odegaard, A. O. *et al.* Dietary patterns and incident type 2 diabetes in chinese men and women: the singapore chinese health study. *Diabetes Care* **34**, 880–885 (9335BC).
151. Polyzos, S. A. *et al.* Effect of spironolactone and vitamin E on serum metabolic parameters and insulin resistance in patients with nonalcoholic fatty liver disease. *J. Renin. Angiotensin. Aldosterone. Syst.* **12**, 498–503 (9228BC).
152. Udani, J. K., Singh, B. B., Singh, V. J. & Barrett, M. L. Effects of Acai (Euterpe oleracea Mart.) berry preparation on metabolic parameters in a healthy overweight population: a pilot study. *Nutr. J.* **10**, 45 (9040BC).
153. Edirisinghe, I. *et al.* Strawberry anthocyanin and its association with postprandial inflammation and insulin. *Br. J. Nutr.* **106**, 913–922 (9036BC).
154. Fung, T. T. *et al.* Whole-grain intake and the risk of type 2 diabetes: a prospective study in men. *Am. J. Clin. Nutr.* **76**, 535–540.
155. Weickert, M. O. *et al.* Effects of supplemented isoenergetic diets differing in cereal fiber and protein content on insulin sensitivity in overweight humans. *Am. J. Clin. Nutr.* **94**, 459–471 (8951BC).
156. Taniguchi-Fukatsu, A. *et al.* Natto and viscous vegetables in a Japanese-style breakfast improved insulin sensitivity, lipid metabolism and oxidative stress in overweight subjects with impaired glucose tolerance. *Br. J. Nutr.* **107**, 1184–1191 (8645BC).
157. Weickert, M. O. *et al.* Changes in dominant groups of the gut microbiota do not explain cereal-fiber induced improvement of whole-body insulin sensitivity. *Nutr. Metab. (Lond).* **8**, 90 (8335BC).

158. Chatterjee, R. *et al.* Potassium intake and risk of incident type 2 diabetes mellitus: the Coronary Artery Risk Development in Young Adults (CARDIA) Study. *Diabetologia* **55**, 1295–1303 (658).
159. Canas, J. A. *et al.* Insulin resistance and adiposity in relation to serum beta-carotene levels. *J. Pediatr.* **161**, 52–58 (676).
160. Cooper, A. J. *et al.* A prospective study of the association between quantity and variety of fruit and vegetable intake and incident type 2 diabetes. *Diabetes Care* **35**, 1293–1300 (851).
161. Cooper, A. J. *et al.* Fruit and vegetable intake and type 2 diabetes: EPIC-InterAct prospective study and meta-analysis. *Eur. J. Clin. Nutr.* **66**, 1082–1092 (1249).
162. Eshak, E. S. *et al.* Soft drink, 100% fruit juice, and vegetable juice intakes and risk of diabetes mellitus. *Clin. Nutr.* **32**, 300–308 (1261).
163. Tsang, C., Smail, N. F., Almoosawi, S., Davidson, I. & Al-Dujaili, E. A. S. Intake of polyphenol-rich pomegranate pure juice influences urinary glucocorticoids, blood pressure and homeostasis model assessment of insulin resistance in human volunteers. *J. Nutr. Sci.* **1**, e9 (1279).
164. Giacco, R. *et al.* Effects of rye and whole wheat versus refined cereal foods on metabolic risk factors: a randomised controlled two-centre intervention study. *Clin. Nutr.* **32**, 941–949 (10654).
165. Kallio, P. *et al.* Inflammation markers are modulated by responses to diets differing in postprandial insulin responses in individuals with the metabolic syndrome. *Am. J. Clin. Nutr.* **87**, 1497–1503.
166. Pan, A., Sun, Q., Manson, J. E., Willett, W. C. & Hu, F. B. Walnut consumption is associated with lower risk of type 2 diabetes in women. *J. Nutr.* **143**, 512–518 (10668).
167. Asemi, Z., Samimi, M., Tabassi, Z., Shakeri, H. & Esmaillzadeh, A. Vitamin D supplementation affects serum high-sensitivity C-reactive protein, insulin resistance, and biomarkers of oxidative stress in pregnant women. *J. Nutr.* **143**, 1432–1438 (11172).
168. Muraki, I. *et al.* Fruit consumption and risk of type 2 diabetes: results from three prospective longitudinal cohort studies. *BMJ* **347**, f5001 (11276).
169. Buscemi, S. *et al.* Association of dietary patterns with insulin resistance and clinically silent carotid atherosclerosis in apparently healthy people. *Eur. J. Clin. Nutr.* **67**, 1284–1290 (11366).
170. Wallace, I. R. *et al.* Dose-response effect of fruit and vegetables on insulin resistance in people at high risk of cardiovascular disease: a randomized controlled trial. *Diabetes Care* **36**, 3888–3896 (11463).
171. Mursu, J., Virtanen, J. K., Tuomainen, T.-P., Nurmi, T. & Voutilainen, S. Intake of fruit, berries, and vegetables and risk of type 2 diabetes in Finnish men: the Kuopio Ischaemic Heart Disease Risk Factor Study. *Am. J. Clin. Nutr.* **99**, 328–333 (11568).
172. Giacco, R. *et al.* A whole-grain cereal-based diet lowers postprandial plasma insulin and triglyceride levels in individuals with metabolic syndrome. *Nutr. Metab. Cardiovasc. Dis.* **24**, 837–844 (20576).
173. Soriano-Maldonado, A., Hidalgo, M., Arteaga, P., de Pascual-Teresa, S. & Nova, E. Effects of regular consumption of vitamin C-rich or polyphenol-rich apple juice on cardiometabolic markers in healthy adults: a randomized crossover trial. *Eur. J. Nutr.* **53**, 1645–1657 (20664).
174. Georgoulis, M. *et al.* The impact of cereal grain consumption on the development and severity of non-alcoholic fatty liver disease. *Eur. J. Nutr.* **53**, 1727–1735 (20755).
175. Frank, L. K. *et al.* Dietary patterns in urban Ghana and risk of type 2 diabetes. *Br. J. Nutr.* **112**, 89–98 (20856).
176. Scheffers, F. R. *et al.* Pure Fruit Juice and Fruit Consumption Are Not Associated with Incidence of Type 2 Diabetes after Adjustment for Overall Dietary Quality in the European Prospective Investigation into Cancer and Nutrition-Netherlands (EPIC-NL) Study. *J. Nutr.* **150**, 1470–1477.
177. Tsitsimpikou, C. *et al.* Dietary supplementation with tomato-juice in patients with metabolic syndrome: a suggestion to alleviate detrimental clinical factors. *Food Chem. Toxicol.* **74**, 9–13 (21351).
178. Cases, J., Romain, C., Dallas, C., Gerbi, A. & Cloarec, M. Regular consumption of Fiit-ns, a polyphenol extract from fruit and vegetables frequently consumed within the Mediterranean diet, improves metabolic ageing of obese volunteers: a randomized, double-blind, parallel trial. *Int. J. Food Sci. Nutr.* **66**, 120–125 (21479).
179. Abenavoli, L. *et al.* Effects of Mediterranean diet supplemented with silybin-vitamin E-phospholipid complex in overweight patients with non-alcoholic fatty liver disease. *Expert Rev. Gastroenterol. Hepatol.* **9**, 519–527 (30571).
180. Moore, L. L., Singer, M. R., Bradlee, M. L. & Daniels, S. R. Adolescent dietary intakes predict cardiometabolic risk clustering. *Eur. J. Nutr.* **55**, 461–468 (30676).
181. Cicero, A. F. G. *et al.* Short-term effects of a combined nutraceutical of insulin-sensitivity, lipid level and indexes of liver steatosis: a double-blind, randomized, cross-over clinical trial. *Nutr. J.* **14**, 30 (30776).
182. O'Neil, C. E., Fulgoni 3rd, V. L. & Nicklas, T. A. Tree Nut consumption is associated with better adiposity measures and cardiovascular and metabolic syndrome health risk factors in U.S. Adults: NHANES 2005-2010. *Nutr. J.* **14**, 64 (31076).
183. Bernabe-Ortiz, A. *et al.* Contribution of modifiable risk factors for hypertension and type-2 diabetes in Peruvian resource-limited settings. *J. Epidemiol. Community Health* **70**, 49–55 (31254).
184. Damsgaard, C. T. *et al.* Associations between school meal-induced dietary changes and metabolic syndrome markers in 8-11-year-old Danish children. *Eur. J. Nutr.* **55**, 1973–1984 (31261).
185. Dourado, G. K. Z. S. & Cesar, T. B. Investigation of cytokines, oxidative stress, metabolic, and inflammatory biomarkers after orange juice consumption by normal and overweight subjects. *Food Nutr. Res.* **59**, 28147 (31468).
186. Canas, J. A., Damaso, L., Hossain, J. & Balagopal, P. B. Fatty acid binding proteins 4 and 5 in overweight prepubertal boys: effect of nutritional counselling and supplementation with an encapsulated fruit and vegetable juice concentrate. *J. Nutr. Sci.* **4**, e39 (31650).
187. Bahadoran, Z., Mirmiran, P., Momenan, A. A. & Azizi, F. Allium vegetable intakes and the incidence of cardiovascular disease, hypertension, chronic kidney disease, and type 2 diabetes in adults: a longitudinal follow-up

study. *J. Hypertens.* **35**, 1909–1916.
188. Muraki, I. *et al.* Potato Consumption and Risk of Type 2 Diabetes: Results From Three Prospective Cohort Studies. *Diabetes Care* **39**, 376–384 (31665).
189. Jia, X. *et al.* Consumption of citrus and cruciferous vegetables with incident type 2 diabetes mellitus based on a meta-analysis of prospective study. *Prim. Care Diabetes* **10**, 272–280 (24982BC).
190. Akbarzadeh, M., Eftekhari, M. H., Shafa, M., Alipour, S. & Hassanzadeh, J. Effects of a New Metabolic Conditioning Supplement on Perioperative Metabolic Stress and Clinical Outcomes: A Randomized, Placebo-Controlled Trial. *Iran. Red Crescent Med. J.* **18**, e26207 (24979BC).
191. AlEssa, H. B. *et al.* High Fiber and Low Starch Intakes Are Associated with Circulating Intermediate Biomarkers of Type 2 Diabetes among Women. *J. Nutr.* **146**, 306–317 (24975BC).
192. Alkerwi, A., Sauvageot, N., Crichton, G. E., Elias, M. F. & Stranges, S. Daily chocolate consumption is inversely associated with insulin resistance and liver enzymes in the Observation of Cardiovascular Risk Factors in Luxembourg study. *Br. J. Nutr.* **115**, 1661–1668 (24771BC).
193. Park, E. *et al.* A dose-response evaluation of freeze-dried strawberries independent of fiber content on metabolic indices in abdominally obese individuals with insulin resistance in a randomized, single-blinded, diet-controlled crossover trial. *Mol. Nutr. Food Res.* **60**, 1099–1109 (24759BC).
194. Buil-Cosiales, P. *et al.* Association between dietary fibre intake and fruit, vegetable or whole-grain consumption and the risk of CVD: results from the PREvencion con DIeta MEDiterranea (PREDIMED) trial. *Br. J. Nutr.* **116**, 534–546 (24482BC).
195. Yoon, H., Jeon, D. J., Park, C. E., You, H. S. & Moon, A. E. Relationship between homeostasis model assessment of insulin resistance and beta cell function and serum 25-hydroxyvitamin D in non-diabetic Korean adults. *J. Clin. Biochem. Nutr.* **59**, 139–144 (24478BC).
196. Mamluk, L. *et al.* Fruit and vegetable intake and risk of incident of type 2 diabetes: results from the consortium on health and ageing network of cohorts in Europe and the United States (CHANCES). *Eur. J. Clin. Nutr.* **71**, 83–91 (24271BC).
197. Asghari, G., Ghorbani, Z., Mirmiran, P. & Azizi, F. Nut consumption is associated with lower incidence of type 2 diabetes: The Tehran Lipid and Glucose Study. *Diabetes Metab.* **43**, 18–24 (23972BC).
198. Liu, S. *et al.* A prospective study of whole-grain intake and risk of type 2 diabetes mellitus in US women. *Am. J. Public Health* **90**, 1409–1415.
199. Bahadoran, Z. *et al.* Vitamin C intake modify the impact of dietary nitrite on the incidence of type 2 diabetes: A 6-year follow-up in Tehran Lipid and Glucose Study. *Nitric oxide Biol. Chem.* **62**, 24–31 (23886BC).
200. Setayeshgar, S. *et al.* Dietary intake and prospective changes in cardiometabolic risk factors in children and youth. *Appl. Physiol. Nutr. Metab.* **42**, 39–45 (23875BC).
201. Ribeiro, C., Dourado, G. & Cesar, T. Orange juice allied to a reduced-calorie diet results in weight loss and ameliorates obesity-related biomarkers: A randomized controlled trial. *Nutrition* **38**, 13–19 (14981BC).
202. Alperet, D. J., Butler, L. M., Koh, W.-P., Yuan, J.-M. & van Dam, R. M. Influence of temperate, subtropical, and tropical fruit consumption on risk of type 2 diabetes in an Asian population. *Am. J. Clin. Nutr.* **105**, 736–745 (14880BC).
203. Koloverou, E. *et al.* Dietary Patterns and 10-year (2002-2012) Incidence of Type 2 Diabetes: Results from the ATTICA Cohort Study. *Rev. Diabet. Stud.* **13**, 246–256 (14878BC).
204. Taghizadeh, M. *et al.* The effects of omega-3 fatty acids and vitamin E co-supplementation on clinical and metabolic status in patients with Parkinson's disease: A randomized, double-blind, placebo-controlled trial. *Neurochem. Int.* **108**, 183–189 (14766BC).
205. Ebrahimi, F. A. *et al.* The Effects of Omega-3 Fatty Acids and Vitamin E Co-Supplementation on Indices of Insulin Resistance and Hormonal Parameters in Patients with Polycystic Ovary Syndrome: A Randomized, Double-Blind, Placebo-Controlled Trial. *Exp. Clin. Endocrinol. Diabetes* **125**, 353–359 (14675BC).
206. den Braver, N. R. *et al.* Determinants of lifestyle behavior change to prevent type 2 diabetes in high-risk individuals. *Int. J. Behav. Nutr. Phys. Act.* **14**, 78 (14476BC).
207. Polyzos, S. A., Kountouras, J., Mantzoros, C. S., Polymerou, V. & Katsinelos, P. Effects of combined low-dose spironolactone plus vitamin E vs vitamin E monotherapy on insulin resistance, non-invasive indices of steatosis and fibrosis, and adipokine levels in non-alcoholic fatty liver disease: a randomized controlled trial. *Diabetes. Obes. Metab.* **19**, 1805–1809 (14378BC).
208. de Mello, V. D. *et al.* Fasting serum hippuric acid is elevated after bilberry (Vaccinium myrtillus) consumption and associates with improvement of fasting glucose levels and insulin secretion in persons at high risk of developing type 2 diabetes. *Mol. Nutr. Food Res.* **61**, (14370BC).
209. Montonen, J. *et al.* Food consumption and the incidence of type II diabetes mellitus. *Eur. J. Clin. Nutr.* **59**, 441–448.
210. Gudjinu, H. Y. & Sarfo, B. Risk factors for type 2 diabetes mellitus among out-patients in Ho, the Volta regional capital of Ghana: a case-control study. *BMC Res. Notes* **10**, 324 (14362BC).
211. Hruby, A. *et al.* Magnesium Intake, Quality of Carbohydrates, and Risk of Type 2 Diabetes: Results From Three U.S. Cohorts. *Diabetes Care* **40**, 1695–1702 (14084BC).
212. Mancini, F. R. *et al.* Dietary antioxidant capacity and risk of type 2 diabetes in the large prospective E3N-EPIC cohort. *Diabetologia* **61**, 308–316 (13979BC).
213. Chen, G.-C., Koh, W.-P., Yuan, J.-M., Qin, L.-Q. & van Dam, R. M. Green leafy and cruciferous vegetable consumption and risk of type 2 diabetes: results from the Singapore Chinese Health Study and meta-analysis. *Br. J. Nutr.* **119**, 1057–1067 (4869BC).
214. Penczynski, K. J. *et al.* Flavonoid intake from fruit and vegetables during adolescence is prospectively associated with a favourable risk factor profile for type 2 diabetes in early adulthood. *Eur. J. Nutr.* **58**, 1159–1172 (4867BC).

215. McKay, D. L., Eliasziw, M., Chen, C. Y. O. & Blumberg, J. B. A Pecan-Rich Diet Improves Cardiometabolic Risk Factors in Overweight and Obese Adults: A Randomized Controlled Trial. *Nutrients* **10**, (4777BC).
216. Mercier, R., Perron, J., Weisnagel, S. J. & Robitaille, J. Associations between fruit and vegetables intake and abnormal glucose tolerance among women with prior gestational diabetes mellitus. *Eur. J. Nutr.* **58**, 689–696 (4766BC).
217. Mancini, F. R. *et al.* Micronutrient dietary patterns associated with type 2 diabetes mellitus among women of the E3N-EPIC (Etude Epidemiologique aupres de femmes de l'Education Nationale) cohort study. *J. Diabetes* **10**, 665–674 (4765BC).
218. Hagele, F. A. *et al.* High orange juice consumption with or in-between three meals a day differently affects energy balance in healthy subjects. *Nutr. Diabetes* **8**, 19 (4663BC).
219. Arab, L. *et al.* Association between walnut consumption and diabetes risk in NHANES. *Diabetes. Metab. Res. Rev.* **34**, e3031 (4377BC).
220. Juntunen, K. S., Laaksonen, D. E., Poutanen, K. S., Niskanen, L. K. & Mykkanen, H. M. High-fiber rye bread and insulin secretion and sensitivity in healthy postmenopausal women. *Am. J. Clin. Nutr.* **77**, 385–391.
221. Kivimaki, M. *et al.* Neighbourhood socioeconomic disadvantage, risk factors, and diabetes from childhood to middle age in the Young Finns Study: a cohort study. *Lancet. Public Heal.* **3**, e365–e373 (4370BC).
222. Adamska-Patruno, E. *et al.* A Synergistic Formulation of Plant Extracts Decreases Postprandial Glucose and Insulin Peaks: Results from Two Randomized, Controlled, Cross-Over Studies Using Real-World Meals. *Nutrients* **10**, (4363BC).
223. Dhillon, J. *et al.* Glucoregulatory and Cardiometabolic Profiles of Almond vs. Cracker Snacking for 8 Weeks in Young Adults: A Randomized Controlled Trial. *Nutrients* **10**, (4363BC).
224. Njike, V. Y. *et al.* The Resulting Variation in Nutrient Intake With the Inclusion of Walnuts in the Diets of Adults at Risk for Type 2 Diabetes: A Randomized, Controlled, Crossover Trial. *Am. J. Health Promot.* **33**, 430–438 (4287BC).
225. Rajaobelina, K. *et al.* Population attributable fractions of the main type 2 diabetes mellitus risk factors in women: Findings from the French E3N cohort. *J. Diabetes* **11**, 242–253 (4165BC).
226. Damiot, A. *et al.* A nutrient cocktail prevents lipid metabolism alterations induced by 20 days of daily steps reduction and fructose overfeeding: result from a randomized study. *J. Appl. Physiol.* **126**, 88–101 (4084BC).
227. Jamilian, M., Sabzevar, N. K. & Asemi, Z. The Effect of Magnesium and Vitamin E Co-Supplementation on Glycemic Control and Markers of Cardio-Metabolic Risk in Women with Polycystic Ovary Syndrome: A Randomized, Double-Blind, Placebo-Controlled Trial. *Horm. Metab. Res.* **51**, 100–105 (4084BC).
228. Lima, A. C. D. *et al.* Effect of Daily Consumption of Orange Juice on the Levels of Blood Glucose, Lipids, and Gut Microbiota Metabolites: Controlled Clinical Trials. *J. Med. Food* **22**, 202–210 (5026).
229. Cai, J. *et al.* Interaction between dietary patterns and TCF7L2 polymorphisms on type 2 diabetes mellitus among Uyghur adults in Xinjiang Province, China. *Diabetes. Metab. Syndr. Obes.* **12**, 239–255 (5126).
230. Sugihiro, T., Yoneda, M., Ohno, H., Oki, K. & Hattori, N. Associations of nutrient intakes with obesity and diabetes mellitus in the longitudinal medical surveys of Japanese Americans. *J. Diabetes Investig.* **10**, 1229–1236 (5131).
231. Davy, B. M. *et al.* High-fiber oat cereal compared with wheat cereal consumption favorably alters LDL-cholesterol subclass and particle numbers in middle-aged and older men. *Am. J. Clin. Nutr.* **76**, 351–358.
232. Bugianesi, E. *et al.* A randomized controlled trial of metformin versus vitamin E or prescriptive diet in nonalcoholic fatty liver disease. *Am. J. Gastroenterol.* **100**, 1082–1090.
233. Ghaderi, A. *et al.* Clinical and metabolic response to vitamin D plus probiotic in schizophrenia patients. *BMC Psychiatry* **19**, 77 (5133).
234. Dow, C. *et al.* Strong adherence to dietary and lifestyle recommendations is associated with decreased type 2 diabetes risk in the AusDiab cohort study. *Prev. Med. (Baltim).* **123**, 208–216 (5219).
235. Yamamoto, J. *et al.* Association Between Okinawan Vegetables Consumption and Risk of Type 2 Diabetes in Japanese Communities: The JPHC Study. *J. Epidemiol.* **30**, 227–235 (5423).
236. Farajbakhsh, A. *et al.* Sesame oil and vitamin E co-administration may improve cardiometabolic risk factors in patients with metabolic syndrome: a randomized clinical trial. *Eur. J. Clin. Nutr.* **73**, 1403–1411 (5426).
237. Vizzari, G. *et al.* Circulating Salicylic Acid and Metabolic Profile after 1-Year Nutritional-Behavioral Intervention in Children with Obesity. *Nutrients* **11**, (5428).
238. Cicero, A. F. G., Fogacci, F., Bove, M., Giovannini, M. & Borghi, C. Three-arm, placebo-controlled, randomized clinical trial evaluating the metabolic effect of a combined nutraceutical containing a bergamot standardized flavonoid extract in dyslipidemic overweight subjects. *Phytother. Res.* **33**, 2094–2101 (5533).
239. Konig, A. *et al.* Guava (Psidium guajava) Fruit Extract Prepared by Supercritical CO2 Extraction Inhibits Intestinal Glucose Resorption in a Double-Blind, Randomized Clinical Study. *Nutrients* **11**, (5615).
240. Gomes-Neto, A. W. *et al.* Fruit and Vegetable Intake and Risk of Posttransplantation Diabetes in Renal Transplant Recipients. *Diabetes Care* **42**, 1645–1652 (5623).
241. Advani, K. *et al.* Efficacy of combination therapy of inositols, antioxidants and vitamins in obese and non-obese women with polycystic ovary syndrome: an observational study. *J. Obstet. Gynaecol.* **40**, 96–101 (5636).
242. Lee, K. W., Woo, H. D., Cho, M. J., Park, J. K. & Kim, S. S. Identification of Dietary Patterns Associated with Incidence of Hyperglycemia in Middle-Aged and Older Korean Adults. *Nutrients* **11**, (5716).
243. Imamura, F. *et al.* Estimated Substitution of Tea or Coffee for Sugar-Sweetened Beverages Was Associated with Lower Type 2 Diabetes Incidence in Case-Cohort Analysis across 8 European Countries in the EPIC-InterAct Study. *J. Nutr.* **149**, 1985–1993.
244. Abdulai, T. *et al.* Prevalence of impaired fasting glucose, type 2 diabetes and associated risk factors in undiagnosed Chinese rural population: the Henan Rural Cohort Study. *BMJ Open* **9**, e029628 (5717).

245. Alae-Carew, C. *et al.* Analysis of dietary patterns and cross-sectional and longitudinal associations with hypertension, high BMI and type 2 diabetes in Peru. *Public Health Nutr.* **23**, 1009–1019 (5740).
246. Papandreou, D. *et al.* Consumption of Raw Orange, 100% Fresh Orange Juice, and Nectar- Sweetened Orange Juice-Effects on Blood Glucose and Insulin Levels on Healthy Subjects. *Nutrients* **11**, (5822).
247. Palacios, O. M. *et al.* Effects of Consuming Almonds on Insulin Sensitivity and Other Cardiometabolic Health Markers in Adults With Prediabetes. *J. Am. Coll. Nutr.* **39**, 397–406 (5828).
248. Kabisch, S. *et al.* Fasting Glucose State Determines Metabolic Response to Supplementation with Insoluble Cereal Fibre: A Secondary Analysis of the Optimal Fibre Trial (OptiFiT). *Nutrients* **11**, (5918).
249. Federico, A. *et al.* Evaluation of the Effect Derived from Silybin with Vitamin D and Vitamin E Administration on Clinical, Metabolic, Endothelial Dysfunction, Oxidative Stress Parameters, and Serological Worsening Markers in Nonalcoholic Fatty Liver Disease Patients. *Oxid. Med. Cell. Longev.* **2019**, 8742075 (5927).
250. Alfawaz, H. *et al.* Improvements in Glycemic, Micronutrient, and Mineral Indices in Arab Adults with Pre-Diabetes Post-Lifestyle Modification Program. *Nutrients* **11**, (6027).
251. Rayner, J., D'Arcy, E., Ross, L. J., Hodge, A. & Schoenaker, D. A. J. M. Carbohydrate restriction in midlife is associated with higher risk of type 2 diabetes among Australian women: A cohort study. *Nutr. Metab. Cardiovasc. Dis.* **30**, 400–409 (6028).
252. Alkutbe, R., Redfern, K., Jarvis, M. & Rees, G. Nutrient Extraction Lowers Postprandial Glucose Response of Fruit in Adults with Obesity as well as Healthy Weight Adults. *Nutrients* **12**, (15226).
253. Ahmed, A., Lager, A., Fredlund, P. & Elinder, L. S. Consumption of fruit and vegetables and the risk of type 2 diabetes: a 4-year longitudinal study among Swedish adults. *J. Nutr. Sci.* **9**, e14 (15314).
254. Mirhashemi, S. M. *et al.* Metabolic Response to Omega-3 Fatty Acids and Vitamin E Co-Supplementation in Patients with Fibrocystic Breast Disease: A Randomized, Double-Blind, Placebo-Controlled Trial. *Arch. Iran. Med.* **20**, 466–473.
255. Wu, C., Liu, P. & Yuan, Z. Fruit and vegetable intake is inversely associated with type 2 diabetes in Chinese women: results from the China Health and Nutrition Survey. *Int. J. Food Sci. Nutr.* **72**, 208–218 (15529).
256. Tabrizi, F. P. F., Farhangi, M. A., Vaezi, M. & Hemmati, S. The effects of spinach-derived thylakoid supplementation in combination with calorie restriction on anthropometric parameters and metabolic profiles in obese women with polycystic ovary syndrome: a randomized, double-blind, placebo-controlled clinical tri. *Nutr. J.* **19**, 82 (15723).
257. Fuglsang-Nielsen, R. *et al.* Effects of whey protein and dietary fiber intake on insulin sensitivity, body composition, energy expenditure, blood pressure, and appetite in subjects with abdominal obesity. *Eur. J. Clin. Nutr.* **75**, 611–619 (15830).
258. DiBella, M. *et al.* Choline Intake as Supplement or as a Component of Eggs Increases Plasma Choline and Reduces Interleukin-6 without Modifying Plasma Cholesterol in Participants with Metabolic Syndrome. *Nutrients* **12**, (15925).
259. Goodman, D. *et al.* Dietary intake and cardiometabolic risk factors among Venezuelan adults: a nationally representative analysis. *BMC Nutr.* **6**, 61 (15928).
260. Jiang, Z. *et al.* Dietary fruit and vegetable intake, gut microbiota, and type 2 diabetes: results from two large human cohort studies. *BMC Med.* **18**, 371 (16115).
261. Agbaria, N. *et al.* Two-Phase Evaluation of a Community-Based Lifestyle Intervention for Palestinian Women in East Jerusalem: A Quasi-Experimental Study Followed by Dissemination. *Int. J. Environ. Res. Public Health* **17**, (16121).
262. Ye, Y. *et al.* A diet rich in fruit and whole grains is associated with a low risk of type 2 diabetes mellitus: findings from a case-control study in South China. *Public Health Nutr.* **25**, 1492–1503 (16127).
263. Larrosa, S. *et al.* Fibre Intake Is Associated with Cardiovascular Health in European Children. *Nutrients* **13**, (16135).
264. Song, S. & Song, Y. Dietary Fiber and Its Source Are Associated with Cardiovascular Risk Factors in Korean Adults. *Nutrients* **13**, (25018).
265. Kochar, J., Djousse, L. & Gaziano, J. M. Breakfast cereals and risk of type 2 diabetes in the Physicians' Health Study I. *Obesity (Silver Spring).* **15**, 3039–3044.
266. Poulos, J. E., Kalogerinis, P. T., Milanov, V., Kalogerinis, C. T. & Poulos, E. J. The Effects of Vitamin E, Silymarin and Carnitine on the Metabolic Abnormalities Associated with Nonalcoholic Liver Disease. *J. Diet. Suppl.* **19**, 287–302 (25037).
267. Teymoori, F. *et al.* A nutrient pattern characterized by vitamin A, C, B6, potassium, and fructose is associated with reduced risk of insulin-related disorders: A prospective study among participants of Tehran lipid and glucose study. *Diabetol. Metab. Syndr.* **13**, 12 (25038).
268. Scheffers, F. R. *et al.* Substitution of pure fruit juice for fruit and sugar-sweetened beverages and cardiometabolic risk in European Prospective Investigation into Cancer and Nutrition (EPIC)-NL: a prospective cohort study. *Public Health Nutr.* **25**, 1504–1514 (25213).
269. Kedarisetty, C. K. *et al.* Efficacy of combining pentoxiphylline and vitamin E versus vitamin E alone in non-alcoholic steatohepatitis- A randomized pilot study. *Indian J. Gastroenterol.* **40**, 41–49 (25239).
270. Galie, S. *et al.* Effects of Mediterranean Diet on plasma metabolites and their relationship with insulin resistance and gut microbiota composition in a crossover randomized clinical trial. *Clin. Nutr.* **40**, 3798–3806 (25339).
271. Madan, J. *et al.* Effect of Almond Consumption on Metabolic Risk Factors-Glucose Metabolism, Hyperinsulinemia, Selected Markers of Inflammation: A Randomized Controlled Trial in Adolescents and Young Adults. *Front. Nutr.* **8**, 668622 (25536).
272. Munoz-Perez, D. M. *et al.* Alternative Foods in Cardio-Healthy Dietary Models That Improve Postprandial Lipemia and Insulinemia in Obese People. *Nutrients* **13**, (25541).

273. Dallio, M. *et al.* PNPLA3, TM6SF2, and MBOAT7 Influence on Nutraceutical Therapy Response for Non-alcoholic Fatty Liver Disease: A Randomized Controlled Trial. *Front. Med.* **8**, 734847 (25920).
274. Ueno, S. *et al.* Association between Dietary Habits and Type 2 Diabetes Mellitus in Yangon, Myanmar: A Case-Control Study. *Int. J. Environ. Res. Public Health* **18**, (25933).
275. Cyunczyk, M. *et al.* Dietary Total Antioxidant Capacity Is Inversely Associated with Prediabetes and Insulin Resistance in Bialystok PLUS Population. *Antioxidants (Basel, Switzerland)* **11**, (30495BC).
276. Czernichow, S. *et al.* Antioxidant supplementation does not affect fasting plasma glucose in the Supplementation with Antioxidant Vitamins and Minerals (SU.VI.MAX) study in France: association with dietary intake and plasma concentrations. *Am. J. Clin. Nutr.* **84**, 395–399.
277. Wang, H. *et al.* Association of gut microbiota with glycaemic traits and incident type 2 diabetes, and modulation by habitual diet: a population-based longitudinal cohort study in Chinese adults. *Diabetologia* **65**, 1145–1156 (30293BC).
278. Barouti, A. A., Tynelius, P., Lager, A. & Bjorklund, A. Fruit and vegetable intake and risk of prediabetes and type 2 diabetes: results from a 20-year long prospective cohort study in Swedish men and women. *Eur. J. Nutr.* (30206BC) doi:10.1007/s00394-022-02871-6.
279. Zhang, X., Xiao, D., Guzman, G., Edirisinghe, I. & Burton-Freeman, B. Avocado consumption for 12 weeks and cardio-metabolic risk factors: a randomized controlled trial in adults with overweight or obesity and insulin resistance. *J. Nutr.* (30010BC) doi:10.1093/jn/nxac126.
280. Alami, F., Alizadeh, M. & Shateri, K. The effect of a fruit-rich diet on liver biomarkers, insulin resistance, and lipid profile in patients with non-alcoholic fatty liver disease: a randomized clinical trial. *Scand. J. Gastroenterol.* 1–12 (30008BC) doi:10.1080/00365521.2022.2071109.
281. Salmeron, J. *et al.* Dietary fiber, glycemic load, and risk of NIDDM in men. *Diabetes Care* **20**, 545–550.
282. Jenkins, D. J. A. *et al.* Effect of almonds on insulin secretion and insulin resistance in nondiabetic hyperlipidemic subjects: a randomized controlled crossover trial. *Metabolism.* **57**, 882–887.
283. Fidelix, M., Milenkovic, D., Sivieri, K. & Cesar, T. Microbiota modulation and effects on metabolic biomarkers by orange juice: a controlled clinical trial. *Food Funct.* **11**, 1599–1610.
284. Meyer, K. A. *et al.* Carbohydrates, dietary fiber, and incident type 2 diabetes in older women. *Am. J. Clin. Nutr.* **71**, 921–930.
285. Simpson, E. J., Mendis, B. & Macdonald, I. A. Orange juice consumption and its effect on blood lipid profile and indices of the metabolic syndrome; a randomised, controlled trial in an at-risk population. *Food Funct.* **7**, 1884–1891.
286. Jiang, R. *et al.* Nut and peanut butter consumption and risk of type 2 diabetes in women. *JAMA* **288**, 2554–2560.
287. Azadbakht, L. *et al.* Soy inclusion in the diet improves features of the metabolic syndrome: a randomized crossover study in postmenopausal women. *Am. J. Clin. Nutr.* **85**, 735–741.
288. Dikariyanto, V. *et al.* Snacking on whole almonds for 6 weeks improves endothelial function and lowers LDL cholesterol but does not affect liver fat and other cardiometabolic risk factors in healthy adults: the ATTIS study, a randomized controlled trial. *Am. J. Clin. Nutr.* **111**, 1178–1189.
289. Cai, J. *et al.* Association of Dietary Patterns with Type 2 Diabetes Mellitus among Middle-Aged Adults in Uygur Population of Xinjiang Region. *J. Nutr. Sci. Vitaminol. (Tokyo).* **65**, 362–374.
290. Doostvandi, T. *et al.* The association of dietary patterns and the incidence of insulin resistance after a 3-year follow-up: Tehran Lipid and Glucose Study. *Asia Pac. J. Clin. Nutr.* **26**, 531–538.
291. Shai, I. *et al.* Ethnicity, obesity, and risk of type 2 diabetes in women: a 20-year follow-up study. *Diabetes Care* **29**, 1585–1590.
292. Khan, N. A. *et al.* Avocado Consumption, Abdominal Adiposity, and Oral Glucose Tolerance Among Persons with Overweight and Obesity. *J. Nutr.* **151**, 2513–2521.
293. Salmeron, J. *et al.* Dietary fiber, glycemic load, and risk of non-insulin-dependent diabetes mellitus in women. *JAMA* **277**, 472–477.
294. Halton, T. L., Liu, S., Manson, J. E. & Hu, F. B. Low-carbohydrate-diet score and risk of type 2 diabetes in women. *Am. J. Clin. Nutr.* **87**, 339–346.
295. Meyer, K. A., Kushi, L. H., Jacobs Jr., D. R. & Folsom, A. R. Dietary fat and incidence of type 2 diabetes in older Iowa women. *Diabetes Care* **24**, 1528–1535.
296. Huang, Y., Park, E., Edirisinghe, I. & Burton-Freeman, B. M. Maximizing the health effects of strawberry anthocyanins: understanding the influence of the consumption timing variable. *Food Funct.* **7**, 4745–4752.
297. Ebrahimi-Mameghani, M. *et al.* Conjugated linoleic acid improves glycemic response, lipid profile, and oxidative stress in obese patients with non-alcoholic fatty liver disease: a randomized controlled clinical trial. *Croat. Med. J.* **57**, 331–342.
298. McMorrow, A. M. *et al.* Personalized Cardio-Metabolic Responses to an Anti-Inflammatory Nutrition Intervention in Obese Adolescents: A Randomized Controlled Crossover Trial. *Mol. Nutr. Food Res.* **62**, e1701008.
299. Wien, M. *et al.* Almond consumption and cardiovascular risk factors in adults with prediabetes. *J. Am. Coll. Nutr.* **29**, 189–197.
300. Montonen, J. *et al.* Dietary patterns and the incidence of type 2 diabetes. *Am. J. Epidemiol.* **161**, 219–227.
301. Aller, R. *et al.* Role of the PNPLA3 polymorphism rs738409 on silymarin + vitamin E response in subjects with non-alcoholic fatty liver disease. *Rev. Esp. Enferm. Dig.* **110**, 634–640.
302. Saboori, S. *et al.* Various Effects of Omega 3 and Omega 3 Plus Vitamin E Supplementations on Serum Glucose Level and Insulin Resistance in Patients with Coronary Artery Disease. *Iran. J. Public Health* **45**, 1465–1472.
303. Weickert, M. O. *et al.* Cereal fiber improves whole-body insulin sensitivity in overweight and obese women. *Diabetes Care* **29**, 775–780.

304. Fung, T. T., Schulze, M., Manson, J. E., Willett, W. C. & Hu, F. B. Dietary patterns, meat intake, and the risk of type 2 diabetes in women. *Arch. Intern. Med.* **164**, 2235–2240.
305. Hu, F. B. *et al.* Diet, lifestyle, and the risk of type 2 diabetes mellitus in women. *N. Engl. J. Med.* **345**, 790–797.
306. Brighenti, F. *et al.* Total antioxidant capacity of the diet is inversely and independently related to plasma concentration of high-sensitivity C-reactive protein in adult Italian subjects. *Br. J. Nutr.* **93**, 619–625.
307. Montonen, J., Knekt, P., Jarvinen, R., Aromaa, A. & Reunanen, A. Whole-grain and fiber intake and the incidence of type 2 diabetes. *Am. J. Clin. Nutr.* **77**, 622–629.
308. Vincent, H. K. *et al.* Effects of antioxidant supplementation on insulin sensitivity, endothelial adhesion molecules, and oxidative stress in normal-weight and overweight young adults. *Metabolism.* **58**, 254–262.
309. Liu, S. *et al.* A Prospective Study of Fruit and Vegetable Intake and the Risk of Type 2 Diabetes in Women. *Diabetes Care* **27**, 2993–2996 (2004).
310. Heidemann, C. *et al.* A dietary pattern protective against type 2 diabetes in the European Prospective Investigation into Cancer and Nutrition (EPIC)—Potsdam Study cohort. *Diabetologia* **48**, 1126–1134 (2005).
311. NOBILI, V. *et al.* Effect of vitamin E on aminotransferase levels and insulin resistance in children with non-alcoholic fatty liver disease. *Aliment. Pharmacol. Ther.* **24**, 1553–1561 (2006).
312. Mercanligil, S. M. *et al.* Effects of hazelnut-enriched diet on plasma cholesterol and lipoprotein profiles in hypercholesterolemic adult men. *Eur. J. Clin. Nutr.* **61**, 212-220 (2007).
313. Krishnan, S. *et al.* Glycemic Index, Glycemic Load, and Cereal Fiber Intake and Risk of Type 2 Diabetes in US Black Women. *Arch. Intern. Med.* **167**, 2304 (2007).
314. Azadbakht, L., Kimiagar, M., Mehrabi, Y. & Zadeh, A. E. Soy inclusion in the diet improves features of the metabolic syndrome: a randomized cross-over study in postmenopausal women. *Iran. J. diabetes lipid Disord.* **7**, E11 (2007).
315. Nobili, V. *et al.* Lifestyle intervention and antioxidant therapy in children with nonalcoholic fatty liver disease: A randomized, controlled trial. *Hepatology* **48**, 119–128 (2008).
316. Djoussé, L., Michael Gaziano, J., Buring, J. E. & Lee, I. M. Egg consumption and risk of type 2 diabetes in men and women. *Diabetes Care* **32**, 295–300 (2009).
317. Stull, A. J., Lastor, K. C., Johnson, W. D., Champagne, C. M. & Cefalu, W. T. Dietary supplementation with blueberry powder enhances insulin sensitivity in insulin resistant humans. (2010).
318. Salas-Salvadó, J. *et al.* Reduction in the incidence of type 2 diabetes with the Mediterranean diet: results of the PREDIMED-Reus nutrition intervention randomized trial. *Diabetes Care* **34**, 14-19 (2011).
319. Esposito, K. *et al.* Effect of a mediterranean-style diet on endothelial dysfunction and markers of vascular inflammation in the metabolic syndrome: a randomized trial. *JAMA* **292**, 1440–1446.
320. Wu, F. Clinical study on prevention efficacy of Jianpi Huatan Fang in treating non-alcoholic fatty liver disease in children. *Zhongguo Zhong yao za zhi [China J. Chinese Mater. medica]* **37**, 2465-2468 (2012).
321. Erratum: Fruit consumption and risk of type 2 diabetes: Results from three prospective longitudinal cohort studies (BMJ (Online)). *BMJ* **347**, (2013).
322. Kurotani, K. *et al.* Vegetable and fruit intake and risk of type 2 diabetes: Japan Public Health Center-based Prospective Study. *Br. J. Nutr.* **109**, 709–717 (2013).
323. Hata, A. *et al.* Magnesium intake decreases Type 2 diabetes risk through the improvement of insulin resistance and inflammation: The Hisayama Study. *Diabet. Med.* **30**, 1487–1494 (2013).
324. Alves, N. E. G., Enes, B. N., Martino, H. S. D., Alfenas, R. D. C. G. & Ribeiro, S. M. R. Meal replacement based on Human Ration modulates metabolic risk factors during body weight loss: A randomized controlled trial. *Eur. J. Nutr.* **53**, 939–950 (2014).
325. InterAct, C. Adherence to predefined dietary patterns and incident type 2 diabetes in European populations: EPIC-InterAct Study. *Diabetologia* **57**, 321–333 (2014).
326. Bekkouche, L., Bouchenak, M., Malaisse, W. J. & Yahia, D. A. The mediterranean diet adoption improves metabolic, oxidative, and inflammatory abnormalities in algerian metabolic syndrome patients. *Horm. Metab. Res.* **46**, 274–282 (2014).
327. Salas-Salvado, J. *et al.* Prevention of diabetes with Mediterranean diets: a subgroup analysis of a randomized trial. *Ann. Intern. Med.* **160**, 1–10 (2014).
328. Semkoff, J. *et al.* The effect of mango supplementation on clinical parameters of pre-diabetic individuals. *FASEB J.* **29**, (2015).
329. McMorrow, A. M. *et al.* Anti-inflammatory dietary intervention selectively improves insulin sensitivity in metabolically unhealthy overweight adolescents. *FASEB J.* **29**, (2015).
330. Villegas, R. *et al.* Vegetable but not fruit consumption reduces the risk of type 2 diabetes in Chinese women. *J. Nutr.* **138**, 574–580.
331. Krishnamma, M., Prasanth Vardhan, S., Naidu, J. N. & Prasad Naidu, M. Study of homeostasis model assessment of insulin resistance, dyslipidemia, antioxidant vitamins status, serum calcium, phosphate and prostate specific antigen in prostate cancer. *Int. J. Pharma Bio Sci.* **6**, B759–B768 (2015).
332. Gonzalez-Anton, C. *et al.* An enriched, cereal-based bread affects appetite ratings and glycemic, insulinemic, and gastrointestinal hormone responses in healthy adults in a randomized, controlled trial. *J. Nutr.* **145**, 231–238 (2015).
333. Gonciulea, A. R. & Sellmeyer, D. The effect of dietary protein source on serum lipids. *Endocr. Rev.* **36**, (2015).
334. Stoupaki, M. *et al.* Beneficial effects of raisin consumption in patients with non-alcoholic fatty liver disease (NAFLD). *Ann. Nutr. Metab. Conf. 12th Eur. Nutr. Conf. FENS 2015. Berlin Ger. Conf. start 20151020. Conf. end 20151023. Conf. Publ.* **67**, 466 (2015).
335. InterAct, C. Dietary fibre and incidence of type 2 diabetes in eight European countries: the EPIC-InterAct Study and a meta-analysis of prospective studies. *Diabetologia* **58**, 1394–1408 (2015).

336. Delgado-Cruzata, L. *et al.* Dietary modifications, weight loss, and changes in metabolic markers affect global DNA methylation in hispanic, african american, and afro-caribbean breast cancer survivors. *J. Nutr.* **145**, 783–790 (2015).
337. Barbour, J. A., Howe, P. R., Buckley, J. D., Bryan, J. & Coates, A. M. Effect of 12 Weeks High Oleic Peanut Consumption on Cardio-Metabolic Risk Factors and Body Composition. *Nutrients* **7**, 7381-7398 (2015).
338. Sawicki, C. M. *et al.* Phytochemical pharmacokinetics and bioactivity of oat and barley flour: A randomized crossover trial. *Nutrients* **8**, (2016).
339. Lankinen, M. *et al.* A healthy Nordic diet alters the plasma lipidomic profile in adults with features of metabolic syndrome in a multicenter randomized dietary intervention. *J. Nutr.* **146**, 662-672 (2016).
340. Amar, M. J. A. *et al.* Randomized double blind clinical trial on the effect of oral α-cyclodextrin on serum lipids. *Lipids Health Dis.* **15**, (2016).
341. O'Neil, C. E., Keast, D. R., Nicklas, T. A. & Fulgoni 3rd, V. L. Nut consumption is associated with decreased health risk factors for cardiovascular disease and metabolic syndrome in U.S. adults: NHANES 1999-2004. *J. Am. Coll. Nutr.* **30**, 502–510.
342. Shahebrahimi, K. *et al.* A comparison of the therapeutic effects of metformin, pioglitazone and vitamin E in patients with non-alcoholic fatty liver. *J. Babol Univ. Med. Sci.* **19**, 32–38 (2017).
343. Canas, J. A. *et al.* Effects of Mixed Carotenoids on Adipokines and Abdominal Adiposity in Children: A Pilot Study. *J. Clin. Endocrinol. Metab.* **102**, 1983–1990 (2017).
344. Kyrø, C., Tjønneland, A., Overvad, K., Olsen, A. & Landberg, R. Higher Whole-Grain Intake Is Associated with Lower Risk of Type 2 Diabetes among Middle-Aged Men and Women: The Danish Diet, Cancer, and Health Cohort. *J. Nutr.* **148**, 1434–1444 (2018).
345. Mazidi, M., Vatanparast, H., Katsiki, N. & Banach, M. The impact of nuts consumption on glucose/insulin homeostasis and inflammation markers mediated by adiposity factors among American adults. *Oncotarget* **9**, 31173–31186 (2018).
346. Urakaze, M. *et al.* Clinical study of astaxanthin on glucose tolerance in nondiabetic subjects. *Diabetes* **67**, A200-(2018).
347. Bandyopadhyay, S., Sinha, S., Selvam, S., Kurpad, A. V & Kuriyan, R. Effect of a cereal and milk meal with or without fruits and nuts on the postprandial glycemic response in Indian men. *Asia Pac. J. Clin. Nutr.* **27**, 1243-1251 (2018).
348. Ginos, B. N. R. *et al.* Circulating bile acids in healthy adults respond differently to a dietary pattern characterized by whole grains, legumes and fruits and vegetables compared to a diet high in refined grains and added sugars: a randomized, controlled, crossover feeding stud. *Metabolism.* **83**, 197-204 (2018).
349. Godwin, N., Roberts, T., Hooshmand, S., Kern, M. & Hong, M. Y. Mixed nuts may promote satiety while maintaining stable blood glucose and insulin in healthy, obese, and overweight adults in a two-arm randomized controlled trial. *J. Med. Food* **22**, 427-432 (2019).
350. Baer, D. J. & Novotny, J. A. Consumption of cashew nuts does not influence blood lipids or other markers of cardiovascular disease in humans: a randomized controlled trial. *Am. J. Clin. Nutr.* **109**, 269-275 (2019).
351. Ponce, O., Benassi, R. & Cesar, T. Orange juice associated with a balanced diet mitigated risk factors of metabolic syndrome: A randomized controlled trial. *J. Nutr. Intermed. Metab.* **17**, (2019).
352. Stevens, J. *et al.* Dietary fiber intake and glycemic index and incidence of diabetes in African-American and white adults: the ARIC study. *Diabetes Care* **25**, 1715–1721.
353. Mirmiran, P., Bahadoran, Z., Tohidi, M. & Azizi, F. Higher consumption of Allium vegetables may modulate insulin homeostasis: A longitudinal follow-up study. *J. Herb. Med.* **17–18**, (2019).
354. Ha, K., Joung, H. & Song, Y. Inadequate fat or carbohydrate intake was associated with an increased incidence of type 2 diabetes mellitus in Korean adults: A 12-year community-based prospective cohort study. *Diabetes Res. Clin. Pract.* **148**, 254–261 (2019).
355. Unal, S. I. & Pekcan, A. G. Effect of almonds consumption on anthropometric measurements and blood parameters in overweight and obese females in a weight reduction program. *Proc. Nutr. Soc.* **79**, (2020).
356. Alam, S. *et al.* Effect of telmisartan and vitamin E on liver histopathology with non-alcoholic steatohepatitis: a randomized, open-label, noninferiority trial. *JGH open an open access J. Gastroenterol. Hepatol.* **4**, 663-669 (2020).
357. West, H., Considine, R., Mattes, R. & Lafayete, W. The effects of long-term almond consumption on body composition and HBA1C stephanie. *Obesity (Silver Spring).* **28**, 167- (2020).
358. Fechner, E. *et al.* Effects of a whole diet approach on metabolic flexibility, insulin sensitivity and postprandial glucose responses in overweight and obese adults - A randomized controlled trial. *Clin. Nutr.* **39**, 2734-2742 (2020).
359. Brayner, B. *et al.* Dietary Patterns Characterized by Fat Type in Association with Obesity and Type 2 Diabetes: A Longitudinal Study of UK Biobank Participants. *J. Nutr.* **151**, 3570–3578 (2021).
360. Bondonno, N. P. *et al.* Associations Between Fruit Intake and Risk of Diabetes in the AusDiab Cohort. *J. Clin. Endocrinol. Metab.* **106**, e4097–e4108 (2021).
361. Palma, X., Thomas-Valdes, S. & Cruz, G. Acute Consumption of Blueberries and Short-Term Blueberry Supplementation Improve Glucose Management and Insulin Levels in Sedentary Subjects. *Nutrients* **13**, (2021).
362. Rosas, M. *et al.* Effects of fresh mango consumption on cardiometabolic risk factors in overweight and obese adults. *Nutr. Metab. Cardiovasc. Dis.* **32**, 494-503 (2022).
363. Feskens, E. J. *et al.* Dietary factors determining diabetes and impaired glucose tolerance. A 20-year follow-up of the Finnish and Dutch cohorts of the Seven Countries Study. *Diabetes Care* **18**, 1104–1112.
364. Barbagallo, M., Dominguez, L. J., Tagliamonte, M. R., Resnick, L. M. & Paolisso, G. Effects of vitamin E and glutathione on glucose metabolism: role of magnesium. *Hypertens. (Dallas, Tex. 1979)* **34**, 1002–1006.
365. Kct. Vitamin C Supplementation Provides Protection from the Adverse Effects of Perfluorinated Compounds on Insulin Resistance in the Elderly. *http://www.who.int/trialsearch/Trial2.aspx?TrialID=KCT0000749* (2013).

366. Cepeda-Lopez, A. C., Melse-Boonstra, A., Zimmermann, M. B. & Herter-Aeberli, I. In overweight and obese women, dietary iron absorption is reduced and the enhancement of iron absorption by ascorbic acid is one-half that in normal-weight women. *Am. J. Clin. Nutr.* **102**, 1389-1397 (2015).
367. Singh, N. *et al.* Effect of antioxidant supplementation on pancreatic functions and markers of fibrosis in chronic pancreatitis: a randomized controlled trial. *Gastroenterology* **150**, S190- (2016).
368. Lee, C. H. *et al.* Dietary intake of anti-oxidant vitamins A, C, and E is inversely associated with adverse cardiovascular outcomes in Chinese—A 22-years population-based prospective study. *Nutrients* **10**, (2018).
369. Hendarto, A., Alhadar, A. K. & Sjarif, D. R. The Effect of Vitamin E Supplementation on Lipid Profiles and Adiponectin Levels in Obese Adolescents: A Randomized Controlled Trial. *Acta Med. Indones.* **51**, 110–116 (2019).
370. Abdelmonem, A., Hosny, H., Ibrahim, M. & El-Siory, W. Comparative study between conventional fasting versus overnight infusion of lipid or carbohydrate on insulin and free fatty-acids in obese patients undergoing elective on-pump coronary artery bypass grafting. a prospective randomized trial. *Clin. Nutr.* **38**, S59- (2019).
371. Bratlie, M. *et al.* Five salmon dinners per week were not sufficient to prevent the reduction in serum vitamin D in autumn at 60° north latitude: A randomised trial. *Br. J. Nutr.* **123**, 419–427 (2020).
372. Akbaraly, T. N., Fontbonne, A., Favier, A. & Berr, C. Plasma carotenoids and onset of dysglycemia in an elderly population: results of the Epidemiology of Vascular Ageing Study. *Diabetes Care* **31**, 1355–1359 (26388).
373. Suriyaprom, K., Kaewprasert, S., Putpadungwipon, P., Namjuntra, P. & Klongthalay, S. Association of antioxidant status and inflammatory markers with metabolic syndrome in Thais. *J. Health. Popul. Nutr.* **38**, 1 (5015).
374. Liu, M. & Park, S. A Causal Relationship between Vitamin C Intake with Hyperglycemia and Metabolic Syndrome Risk: A Two-Sample Mendelian Randomization Study. *Antioxidants (Basel, Switzerland)* **11**, (30197BC).
375. Paolisso, G. *et al.* Chronic intake of pharmacological doses of vitamin E might be useful in the therapy of elderly patients with coronary heart disease. *Am. J. Clin. Nutr.* **61**, 848–852.
376. Mejean, C. *et al.* Diet quality of North African migrants in France partly explains their lower prevalence of diet-related chronic conditions relative to their native French peers. *J. Nutr.* **137**, 2106–2113.
377. Badiou, S. *et al.* Fenofibrate improves the atherogenic lipid profile and enhances LDL resistance to oxidation in HIV-positive adults. *Atherosclerosis* **172**, 273-279 (2004).
378. Stone, P. H. *et al.* Effect of intensive lipid lowering, with or without antioxidant vitamins, compared with moderate lipid lowering on myocardial ischemia in patients with stable coronary artery disease: the Vascular Basis for the Treatment of Myocardial Ischemia Study. *Circulation* **111**, 1747-1755 (2005).
379. Singh, U. *et al.* High-dose alpha-tocopherol therapy does not affect HDL subfractions in patients with coronary artery disease on statin therapy. *Clin. Chem.* **53**, 525-528 (2007).
380. Chuin, A. *et al.* Effect of antioxidants combined to resistance training on BMD in elderly women: a pilot study. *Osteoporos. Int.* **20**, 1253-1258 (2009).
381. Fallahzadeh, M. K., Akbari, H., Sohrabi Nazari, S. & Sagheb, M. M. Efficacy of vitamins C, E and their combination for treatment of restless legs syndrome in hemodialysis patients; A randomized, double-blind, placebo-controlled trial. *Iran. J. Kidney Dis.* **5**, 22-23 (2011).
382. Sagheb, M. M. *et al.* Efficacy of vitamins C, E, and their combination for treatment of restless legs syndrome in hemodialysis patients: a randomized, double-blind, placebo-controlled trial. *Sleep Med.* **13**, 542-545 (2012).
383. Salonen, J. T. & Korpela, H. Vitamin E and non-insulin dependent diabetes mellitus. *Diabetes und Stoffwechsel* **6**, 34–37 (1997).
384. Deas, G. W. Contribution of diet to the increased incidence of type 2 diabetes mellitus in inner city African-American children. *J. Pediatr. Endocrinol. Metab.* **15**, 503–504 (2002).
385. Patrick Basu, P. *et al.* Abstracts of the Congress 12–15 June 2012 Reykjavik, Iceland. *Scand. J. Gastroenterol.* **47**, S14–S84 (2012).
386. Young, I. S. *et al.* Basic and clinical science posters. *Diabet. Med.* **29**, 30–72 (2012).
387. Basu, P., James Shah, N. & Farhat, S. Effect of vitamin E and alfa lipoic acid (ALA) in non-alcoholic fatty liver disease: a randomise placebo control open label prospective clinical trial: v A I N trial. *Gut* **61**, A204 (2012).
388. Ctri. Nutritional supplement for women with Polycystic Ovary Syndrome or subfertilty. *https://trialsearch.who.int/Trial2.aspx?TrialID=CTRI/2012/08/002943* (2012).
389. Patrick Basu, P. *et al.* Curcumin, anti-oxidant, and pioglitazone therapy with inclusion of vitamin E in non-alcoholic fatty liver disease-a randomized double blind placebo controlled trial (CAPTIVE). *Hepatol. Int.* **7**, S73-S74 (2013).
390. Basu, P. *et al.* Curcumin, anti-oxidant, and pioglitazone therapy with inclusion of vitamin e in non-alcoholic fatty liver disease-a randomized open label placebo controlled clinical prospective trial (Captive). *J. Clin. Exp. Hepatol.* **3**, S26-S27 (2013).
391. Joshi, S. S., Mehta, A. & Joshi, S. Behavioral Medicine, Clinical Nutrition, Education, and Exercise. *Diabetes* **63**, A170–A212 (2014).
392. Connaughton *et al.* Personalised nutrition perspectives-anti-inflammatory nutritional intervention selectively improves insulin sensitivity in overweight and obese adolescents wherein baseline metabotype predicts response. *Obes. Facts* **7**, 10 (2014).
393. Karamali, M., Samimi, M., Bahmani, F., Foroozanfard, F. & Esmaillzadeh, A. The effects of DASH diet on lipid profiles and biomarkers of oxidative stress in overweight and obese women with polycystic ovary syndrome: a randomised clinical trial. *Hum. Reprod.* **29**, i315-i316 (2014).
394. Kedarisetty, C. K. *et al.* A randomized controlled trial to study the efficacy of combination of pentoxiphylline and vitamin e versus vitamin e in patients with non-alcoholic steatohepatitis. *J. Hepatol.* **60**, S344- (2014).
395. Dietary intervention offers additional benefits to statins in high cholesterol. *Pharm. J.* **268**, 200- (2002).
396. Canas, J. *et al.* Effect of a 2-week intense life-style intervention followed by 6-month carotenoid supplementation on fat depots, aditonectin and palmitoleate: a 6-month double blind placebo-controlled pilot study in obese children.

FASEB J. **28**, (2014).
397. Hernandez-Alonso, P. *et al.* 21th European Congress on Obesity (ECO2014), Sofia, Bulgaria, May 28-31, 2014: Abstracts. *Obes. Facts* **7**, 1–188 (2014).
398. Angelino, D. *et al.* Antinflammatory and antidiabetic effects of a whole grain pasta enriched in prebiotics and probiotics. *Ann. Nutr. Metab.* **67**, 420 (2015).
399. Lee, Y. *et al.* Effects of polyphenolic-rich dark chocolate and almonds on cardiovascular risk factors in overweight and obese adults. *FASEB journal. Conf. Exp. Biol. 2016, EB. San diego, CA united states. Conf. start 20160402. Conf. end 20160406. Conf. Publ.* **30**, (2016).
400. Den Braver, N. R. *et al.* Determinants of lifestyle behaviour change to prevent type 2 diabetes in high-risk subjects. *Diabetologia* **59**, S143-S144 (2016).
401. jx7j, R. B. R. Metabolic, inflammatory, genetic and satiety response to consumption of monounsaturated fatty acids and fructose. *https://trialsearch.who.int/Trial2.aspx?TrialID=RBR-66jx7j* (2016).
402. Ebrahimi, F. A. Erratum: The Effects of Omega-3 Fatty Acids and Vitamin E Co-Supplementation on Indices of Insulin Resistance and Hormonal Parameters in Patients with Polycystic Ovary Syndrome: A Randomized, Double-Blind, Placebo-Controlled Trial (Experimental and Clinic. *Exp. Clin. Endocrinol. Diabetes* **125**, E3 (2017).
403. Hagele, F. *et al.* 24th European Congress on Obesity (ECO2017), Porto, Portugal, May 17-20, 2017: Abstracts. *Obes. Facts* **10**, 1–274 (2017).
404. Ctri. Effect of Almonds on Blood Glucose and Cholesterol levels among Overweight Individuals. *https://trialsearch.who.int/Trial2.aspx?TrialID=CTRI/2017/10/010251* (2017).
405. Cicero, A., Fogacci, F., Bove, M., Giovannini, M. & Borghi, C. Three-arm, placebo controlled, randomized clinical trial evaluating the metabolic effect of a bergamot standardized flavonoid extract in dyslipidemic overweight subjects. *Atherosclerosis* **287**, e32-e33 (2019).
406. Federico, A. *et al.* A new silybin-vitamin E-phospholipid complex improves insulin resistance and liver damage in patients with non-alcoholic fatty liver disease: preliminary observations. *Gut* **55**, 901-902 (2006).
407. Kedarisetty, C. K. *et al.* An open label randomized controlled trial to study the efficacy of combination of pentoxiphylline and vitamin E versus vitamin E alone in patients with non-alcoholic steatohepatitis. *J. Gastroenterol. Hepatol.* **34**, 102- (2019).
408. Shidfar, F. *et al.* E-Poster Presentations (Oral) | APDW 2019. *J. Gastroenterol. Hepatol.* **34**, 72–582 (2019).
409. Cesar, T., Fidelix, M., Sivieri, K. & Millenkovic, D. Daily consumption of orange juice modulated intestinal microbiota and improved glucose and lipids metabolism in women. *Proc. Nutr. Soc.* **79**, (2020).
410. Dallio, M. *et al.* Evaluation of the effect derived from silybin with vitamin D and 3 vitamin E administration on clinical, metabolic, endothelial 4 dysfunction, oxidative stress parameters and serological worsening 5 markers in non-alcoholic fatty liver disease patients. *Dig. liver Dis.* **52**, S165- (2020).
411. Dikariyanto, V. *et al.* The effects of whole almond snack consumption on fasting blood lipids and insulin sensitivity: a randomised controlled trial in adults. *Proc. Nutr. Soc.* **79**, E5- (2020).
412. Campos-Borges, C. *et al.* Targeting antioxidant strategies for nonproliferative diabetic retinopathy. *Invest. Ophthalmol. Vis. Sci.* **62**, (2021).
413. fghxtm, R. B. R. Effects of exercise and antioxidant supplementation in individuals with diabetes kidney disease. *https://trialsearch.who.int/Trial2.aspx?TrialID=RBR-10fghxtm* (2021).
414. Salmasi, F. B. *et al.* Comparing the Effects of High-Dose Vitamin E With Those of Placebo on Insulin Resistance in Patients With Schizophrenia Treated With Olanzapine. *J. Clin. Psychopharmacol.* **29**, 182–183 (2009).
415. Hercberg, S., Czernichow, S. & Galan, P. Vitamin C Concentration and Type 2 Diabetes Mellitus. *Arch. Intern. Med.* **169**, 633 (2009).
416. Basu, P. P., Rayapudi, K., Pacana, T., Ramamurthy, S. & Brown Jr, R. A randomized open label clinical trial with oral alfa lipoic acid and vitamin e in non alcoholic fatty liver disease and non alcoholic steatohepatitis. *J. Hepatol.* **50**, S356 (2009).
417. Ctri. To see the effect of antioxidants(combination of vitamins and nutrients that protect our cells) on the functions of the pancreas(endocrine and exocrine)in patients with chronic pancreatitis. *https://trialsearch.who.int/Trial2.aspx?TrialID=CTRI/2011/05/001755* (2011).
418. Basu, P. P., Krishnaswamy, N., Nair, T., Shah, N. J. & Farhat, S. Effect of vitamin E and alfa lipoic acid (ALA) in non alcoholic fatty liver disease: a randomized placebo control open label prospective clinical trial - VAIN trial. *Am. J. Gastroenterol.* **106**, S136-S137 (2011).
419. Canas, J. A., Damaso, L., Altomare, A., Hossain, J. & Balagopal, B. Effect of a fruit and vegetable juice concentrate (FVJC) Vs. medical nutrition therapy alone on metabolic syndrome components in obese children: a 6 month pilot double blind placebo-controlled study. *FASEB J.* **25**, (2011).
420. Antoniades, C. *et al.* Vascular endothelium and inflammatory process, in patients with combined Type 2 diabetes mellitus and coronary atherosclerosis: the effects of vitamin C. *Diabet. Med.* **21**, 552–558.
421. Day, R. & Lal, S. S. Supplementation effects of vitamin C and vitamin E on oxidative stress in post menopausal diabetic women. *J. Appl. Res.* **12**, 108–111 (2012).
422. Ley, S. H., Hanley, A. J., Sermer, M., Zinman, B. & O'Connor, D. L. Lower dietary vitamin E intake during the second trimester is associated with insulin resistance and hyperglycemia later in pregnancy. *Eur. J. Clin. Nutr.* **67**, 1154–1156 (11373).
423. Bonaccio, M. *et al.* Adherence to the traditional Mediterranean diet and mortality in subjects with diabetes. Prospective results from the MOLI-SANI study. *Eur. J. Prev. Cardiol.* **23**, 400–407 (30651).
424. Asemi, Z. *et al.* Effect of the omega-3 fatty acid plus vitamin E supplementation on subjective global assessment score, glucose metabolism, and lipid concentrations in chronic hemodialysis patients. *Mol. Nutr. Food Res.* **60**, 390–398 (31571).

425. Stonehouse, W., Brinkworth, G. D., Thompson, C. H. & Abeywardena, M. Y. Short term effects of palm-tocotrienol and palm-carotenes on vascular function and cardiovascular disease risk: A randomised controlled trial. *Atherosclerosis* **254**, 205–214 (24074BC).
426. Fatima, N. *et al.* Emerging role of Interleukins IL-23/IL-17 axis and biochemical markers in the pathogenesis of Type 2 Diabetes: Association with age and gender in human subjects. *Int. J. Biol. Macromol.* **105**, 1279–1288 (14361BC).
427. Baumgartner, S. *et al.* The effects of vitamin E or lipoic acid supplementation on oxyphytosterols in subjects with elevated oxidative stress: a randomized trial. *Sci. Rep.* **7**, 15288 (13978BC).
428. Kerr, D. *et al.* Farming for life: impact of medical prescriptions for fresh vegetables on cardiometabolic health for adults with or at risk of type 2 diabetes in a predominantly Mexican-American population. *BMJ Nutr. Prev. Heal.* **3**, 239–246 (15917).
429. Chen, Y. *et al.* Fresh fruit consumption, physical activity, and five-year risk of mortality among patients with type 2 diabetes: A prospective follow-up study. *Nutr. Metab. Cardiovasc. Dis.* **32**, 878–888 (26020).
430. Das, D., Sen, S., Bhakta, S. & Sen, K. Preclusion of methemoglobinemia caused by nitrate drugs in diabetics and nondiabetics: Possible role of Vitamin C. *Blood Cells. Mol. Dis.* **94**, 102643 (30519BC).
431. Asemi, Z., Samimi, M., Tabassi, Z., Sabihi, S. S. & Esmaillzadeh, A. A randomized controlled clinical trial investigating the effect of DASH diet on insulin resistance, inflammation, and oxidative stress in gestational diabetes. *Nutrition* **29**, 619–624 (2013).
432. Cercamondi, C. I. *et al.* Iron bioavailability from a lipid-based complementary food fortificant mixed with millet porridge can be optimized by adding phytase and ascorbic acid but not by using a mixture of ferrous sulfate and sodium iron EDTA. *J. Nutr.* **143**, 1233-1239 (2013).
433. Mahmoodi, M. R., Kimiagar, M. & Mehrabi, Y. The effects of omega-3 plus vitamin E and zinc plus vitamin C supplementation on cardiovascular risk markers in postmenopausal women with type 2 diabetes. *Ther. Adv. Endocrinol. Metab.* **5**, 67-76 (2014).
434. Singh, N. *et al.* Antioxidants for Pancreatic Functions in Chronic Pancreatitis: A Double-blind Randomized Placebo-controlled Pilot Study. *J. Clin. Gastroenterol.* **54**, 284–293 (2020).
435. Dawood, M. H. & Al-Yasiri, T. H. Article effects of ascorbic acid on insulin resistance in hyper insulinemic and euglycemic persons. *Int. J. Drug Deliv. Technol.* **11**, 1008–1011 (2021).
436. Shargorodsky, M., Debby, O., Matas, Z. & Zimlichman, R. Effect of long-term treatment with antioxidants (vitamin C, vitamin E, coenzyme Q10 and selenium) on arterial compliance, humoral factors and inflammatory markers in patients with multiple cardiovascular risk factors. *Nutr. Metab. (Lond).* **7**, 55 (18846BC).
437. Brazionis, L., Walker, K. Z., Itsiopoulos, C. & O'Dea, K. Plasma retinol: a novel marker for cardiovascular disease mortality in Australian adults. *Nutr. Metab. Cardiovasc. Dis.* **22**, 914–920 (8426BC).
438. Hegde, S. V, Adhikari, P., M, N. & D'Souza, V. Effect of daily supplementation of fruits on oxidative stress indices and glycaemic status in type 2 diabetes mellitus. *Complement. Ther. Clin. Pract.* **19**, 97–100 (10566).
439. Manning, P. J. *et al.* Effect of High-Dose Vitamin E on Insulin Resistance and Associated Parameters in Overweight Subjects. *Diabetes Care* **27**, 2166–2171 (2004).
440. Ou, Y. *et al.* Associations of serum vitamin C concentrations with risk of all-cause and cause-specific mortality among individuals with and without type 2 diabetes. *Eur. J. Nutr.* **17**, 17 (2023).
441. Kim, J. H. *et al.* The modifying effect of vitamin C on the association between perfluorinated compounds and insulin resistance in the Korean elderly: a double-blind, randomized, placebo-controlled crossover trial. *Eur. J. Nutr.* **55**, 1011–1020 (30953).
442. Martínez-Abundis, E., Pascoe-González, S., González-Ortiz, M., Mora-Martínez, J. M. & Cabrera-Pivaral, C. E. Effect of the oral administration of ascorbic acid on lipid profile and insulin sensitivity in obese people. *Rev. Investig. Clin.* **53**, 505–510 (2001).
443. Aldámiz-Echevarría, L. *et al.* A randomized single-blind trial of the effects of vitamins C and E in familial hypercholesterolemia. *An. Pediatr. (Barc).* **65**, 101-107 (2006).
444. Shidfar, F., Rezai, K. H., Hosseini, S. H. & Haydari, I. The effects of vitamin E on insulin resistance and cardiovascular diseases risk factors in metabolic syndrome. *Iran. J. Endocrinol. Metab.* **10**, 445-454 (2009).
445. Kouchaki, E. *et al.* Retracted: High-dose ω-3 Fatty Acid Plus Vitamin D3 Supplementation Affects Clinical Symptoms and Metabolic Status of Patients with Multiple Sclerosis: A Randomized Controlled Clinical Trial. *J. Nutr.* **148**, 1380–1386 (2018).
